# Impact of Dietary Inclusion with Cocrystal Essential Oil on Growth Performance, Nutrient Digestibility, Intestinal Morphology, and Antioxidant Status in Weaned Piglets

**DOI:** 10.3390/ani16091400

**Published:** 2026-05-03

**Authors:** Yifei Sun, Jun Chen, Qiuting Yin, Pengbo Liang, Jinming You, Tiande Zou

**Affiliations:** Jiangxi Province Key Laboratory of Animal Nutrition and Feed, College of Animal Science and Technology, Jiangxi Agricultural University, Nanchang 330045, China; 13077994121@163.com (Y.S.); junchen@jxau.edu.cn (J.C.); yqt1652900244@163.com (Q.Y.); 22417024@zju.edu.cn (P.L.); youjinm@jxau.edu.cn (J.Y.)

**Keywords:** antioxidant status, cocrystal essential oil, growth performance, intestinal morphology, nutrient digestibility, weaned piglets

## Abstract

This study assessed the impact of cocrystal essential oil (CEO) inclusion on growth performance, nutrient digestibility, intestinal morphology, and antioxidant status in weaned piglets. Ninety-six weaned piglets were assigned to four groups (n = 8, three piglets per pen). The piglets in the four groups were fed basal diets added with 0, 120, 180, or 240 mg/kg of CEO, respectively, over a 28-day trial period. Results showed that during weeks 1–2, piglets in the 240 mg/kg CEO group exhibited a lower diarrhea rate and diarrhea index compared to the control group (*p* < 0.05). In weeks 3–4, the 180 and 240 mg/kg CEO groups demonstrated a reduced diarrhea rate and diarrhea index compared to the control group (*p* < 0.05). Relative to the control group, the apparent total tract digestibility (ATTD) of dry matter was elevated in piglets fed diets added with 120, 180, or 240 mg/kg CEO at both day 14 and day 28 (*p* < 0.05). Additionally, the ATTD of crude protein was elevated in the 120 mg/kg CEO group at day 14 and in the 180 mg/kg CEO group at day 28 (*p* < 0.05). Regarding intestinal morphology, supplementation with 180 or 240 mg/kg CEO increased jejunal villus height (VH) and the villus height to crypt depth (VH/CD) ratio compared with the control group (*p* < 0.05). Furthermore, 240 mg/kg CEO supplementation augmented the ileal VH/CD ratio relative to the control group (*p* < 0.05). For antioxidant status, 180 mg/kg CEO supplementation elevated serum glutathione peroxidase (GSH-Px) activity in piglets relative to the control group (*p* < 0.05). Importantly, no differences were found between the 180 mg/kg and 240 mg/kg CEO groups across all measured parameters (*p* > 0.05). In conclusion, dietary inclusion with 180 mg/kg CEO is recommended for weaned piglets, given its comprehensive benefits in alleviating diarrhea, improving nutrient digestibility, enhancing intestinal morphology, and bolstering antioxidant status.

## Simple Summary

Early weaning is a prevalent management strategy in contemporary pig production, yet early-weaned piglets encounter numerous challenges. Consequently, exploring effective feed additives for early-weaned piglets is urgently needed. The current study indicated that dietary inclusion with 180 mg/kg cocrystal essential oil is recommended for weaned piglets, conferring comprehensive benefits including alleviation of diarrhea, enhanced nutrient digestibility, improved intestinal morphology, and reinforced antioxidant status.

## 1. Introduction

Early weaning is a widely adopted management practice in modern swine production [1]. Weaning is one of the most critical periods for pigs [2], during which early-weaned piglets exhibit underdeveloped gastrointestinal tracts [3] and immature antioxidant systems [4], making them particularly susceptible to multiple challenges. These challenges encompass poor nutrient digestibility, heightened susceptibility to pathogen infection, increased incidence of post-weaning diarrhea, and, consequently, compromised growth performance [5,6]. In-feed antibiotics were once widely used as anti-diarrheal agents and growth promoters for early-weaned piglets [7]. However, due to concerns regarding antibiotic resistance, their use has been banned in many regions, such as the European Union [8] and China [9]. As such, there is an urgent need to seek efficient feed additives for early-weaned piglets.

Of note, essential oils are promising feed additives for alleviating weaning stress in early-weaned piglets [10,11]. For example, Li et al. (2012) observed that inclusion with essential oil (thymol and cinnamaldehyde) improved weight gain and fecal consistency, increased the jejunal villus height-to-crypt depth ratio, enhanced nutrient digestibility of dry matter and crude protein, and elevated serum total antioxidant capacity in weaned piglets [12]. Importantly, these beneficial effects were comparable to those observed in the in-feed antibiotic group [12]. Shao et al. (2023) also found that a four-week dietary inclusion with an essential oil blend (carvacrol, thymol, and cinnamaldehyde) improved growth performance and jejunal and ileal morphology without affecting diarrhea scores and serum antioxidant status of weaned piglets [13]. Nevertheless, the direct incorporation of essential oils into pig diets has limited efficacy owing to their chemical instability, unfavorable palatability, and poor bioavailability within the gastrointestinal tract [14]. Therefore, developing efficient and feasible delivery methods is critical for the practical application of essential oils in pig production [14].

Cocrystal technology offers a promising solution to these limitations [15]. As an advanced approach in pharmaceutical formulation, cocrystal technology can significantly improve critical drug properties, such as solubility, stability, and bioavailability [16]. For instance, meloxicam–aspirin cocrystals exhibited a 12-fold more rapid onset of action and superior oral bioavailability relative to the pure drug in a rat model [17]. Li et al. (2023) demonstrated that addition with 30, 60, or 120 mg/kg cocrystal essential oil (CEO) for 42 days increased average daily gain without affecting feed intake of broilers [18]. Yang et al. (2025) also observed that dietary supplementation with 80 mg/kg of CEO (thymol and carvacrol) for 42 days improved feed intake, serum antioxidant status, and intestinal morphology in broilers [19]. Li et al. (2024) further documented that dietary inclusion with 60 mg/kg CEO (thymol and carvacrol) for 42 days improved meat quality, nutritional value, and antioxidant stability in broiler meat [20]. In pigs, Ma et al. (2026) found that dietary addition with 100 mg/kg of CEO (thymol and carvacrol) over 84 days improved pork quality and antioxidant ability in finishing pigs [21]. However, the potential beneficial effects of CEO in weaned piglets remain insufficiently explored. Therefore, ninety-six newly weaned piglets were used to investigate the effects of dietary inclusion with CEO on growth performance, diarrhea conditions, nutrient digestibility, intestinal morphology, and antioxidant status in weaned piglets.

## 2. Materials and Methods

### 2.1. Study Design

Ninety-six DLY male piglets, newly weaned at 21 days of age (5.80 ± 0.01 kg), were allocated to 4 groups based on body weight, with 8 replicates per group and 3 piglets per replicate. The piglets in the 4 groups were fed basal diets supplemented with 0, 120, 180, or 240 mg/kg cocrystal essential oil (CEO) for 28 days, respectively. The supplemental dosage levels were selected based on a previous study of CEO in finishing pigs (100 mg/kg) [21], while taking into account the high stress susceptibility and physiological vulnerability of weaned piglets. Accordingly, a graded dosage regimen of 120, 180, and 240 mg/kg was adopted. The CEO product is a mixture in equal parts of two cocrystals, thymol–L-proline (1:1 molar ratio) and carvacrol–L-proline (1:1 molar ratio), formulated with calcium phosphate as a carrier. The CEO product is supplied by Cocrystal Health Co., Ltd. (Jiashan, China). Both thymol and carvacrol are extracted from oregano oil, with a combined effective content of 25% in the final CEO product. Throughout the 28-day feeding trial, piglets were free access to fresh water and their respective experimental diets. All diets were formulated to meet NRC (2012) [22] nutrient requirements for piglets. Details of the basal diet composition are provided in Table 1.

### 2.2. Data Recording and Sample Collection

#### 2.2.1. Growth Performance

On days 14 and 28, all piglets were weighed to measure average daily gain (ADG) during weeks 1–2 and 3–4. Feed consumption was recorded per pen to determine average daily feed intake (ADFI) for the corresponding periods, and feed conversion ratio (FCR) was subsequently calculated based on feed intake and weight gain.

#### 2.2.2. Diarrhea Assessment

Fecal consistency was scored daily on a per-pen basis based on the method described by Yu et al. (2020) [23], using the following scoring system: 0 = well-formed feces or pellets (normal), 1 = soft feces (slight diarrhea), 2 = unformed feces (moderate diarrhea), and 3 = watery feces (severe diarrhea). Diarrhea was defined as a fecal score ≥ 2. The diarrhea rate and diarrhea index were calculated using the following formulas: diarrhea rate = [cumulative (diarrheic pigs per pen × duration of diarrhea)/(total pig count ×  observation days)  ×  100%; diarrhea index  =  cumulative of diarrhea scores per pen/(pigs per pen × observation days).

#### 2.2.3. Fecal Sample Collection

Feces were collected from all piglets on days 12–14 and 26–28 of the experiment and preserved with 10 mL of 10% sulfuric acid per 100 g of feces. Experimental diets were also sampled from each group on the same days. The feces collected over the 3-day period were weighed, pooled, and then subsampled, oven-dried, and ground for determination of dry matter, crude protein, crude fat, and crude ash.

#### 2.2.4. Blood Sample Collection

At the end of the feeding trial, one piglet per pen (with body weight close to the pen average) was selected for blood collection via anterior vena cava puncture. Approximately 10 mL of blood was collected from each piglet. The blood samples were placed at a 45° angle for 30 min at room temperature to allow clotting, then centrifuged at 4000 rpm for 15 min at 4 °C. The resulting serum was transferred to Eppendorf tubes and stored at −80 °C for antioxidant parameter analysis.

#### 2.2.5. Intestinal Tissue Collection

After blood sampling, piglets were humanely euthanized by intravenous injection of sodium pentobarbital. The abdominal cavity was opened, and the intestinal tract was carefully removed. Three-centimeter segments were sampled from the mid-duodenum, mid-jejunum, and mid-ileum, fixed in 4% paraformaldehyde in 15 mL centrifuge tubes, and stored at room temperature. The fixative was replaced with fresh solution after 24 h, and the samples were maintained at room temperature for subsequent intestinal morphological measurement.

### 2.3. Laboratory Analysis

#### 2.3.1. Nutrient Digestibility Analysis

The contents of dry matter, crude protein, crude fat, and crude ash in feed and feces were determined based on the National Standards of the People’s Republic of China: GB/T 6435-2006 [24], GB/T 6432-1994 [25], GB/T 6433-2006 [26], and GB/T 6438-2007 [27], respectively. These methods were also referenced by Wu et al. (2025) [28]. Acid-insoluble ash was used as an endogenous indicator to calculate apparent total tract digestibility (ATTD) [29], using the following formula: ATTD = [1 − (acid-insoluble ash content in the diets × nutrient content in the feces)/(acid-insoluble ash content in the feces × nutrient content in the diets)] × 100%.

#### 2.3.2. Intestinal Morphology Measurement

After fixation, intestinal samples were dehydrated, cleared, and embedded in paraffin for sectioning. The sections were stained with hematoxylin and eosin (H&E) and examined under a microscope (DM300, Leica, Wetzlar, Germany) to observe intestinal morphology. Five representative fields per section were photographed. Villus height (VH) and crypt depth (CD) were measured using Image-Pro Plus 6.0 software, and the villus height to crypt depth (VH/CD) ratio was calculated.

#### 2.3.3. Serum Antioxidant Parameter Analysis

Serum antioxidant parameters were measured with commercial kits (Nanjing Jiancheng Bioengineering Institute, Nanjing, China) based on the manufacturer’s instructions. The parameters included malondialdehyde (MDA) level, total antioxidant capacity (T-AOC), catalase (CAT) activity, total superoxide dismutase (T-SOD) activity, and glutathione peroxidase (GSH-Px) activity.

### 2.4. Statistical Analysis

Statistical analyses were conducted using SPSS 25.0 (Chicago, IL, USA). Data were analyzed by one-way ANOVA with Duncan’s multiple range test for post hoc comparisons. Polynomial contrasts were employed to evaluate the linear dose–response effects of CEO supplementation. Statistical significance was set at *p* < 0.05, and 0.05 ≤ *p* < 0.10 was considered a trend toward significance.

## 3. Results

### 3.1. Growth Performance

**Figure 1:**
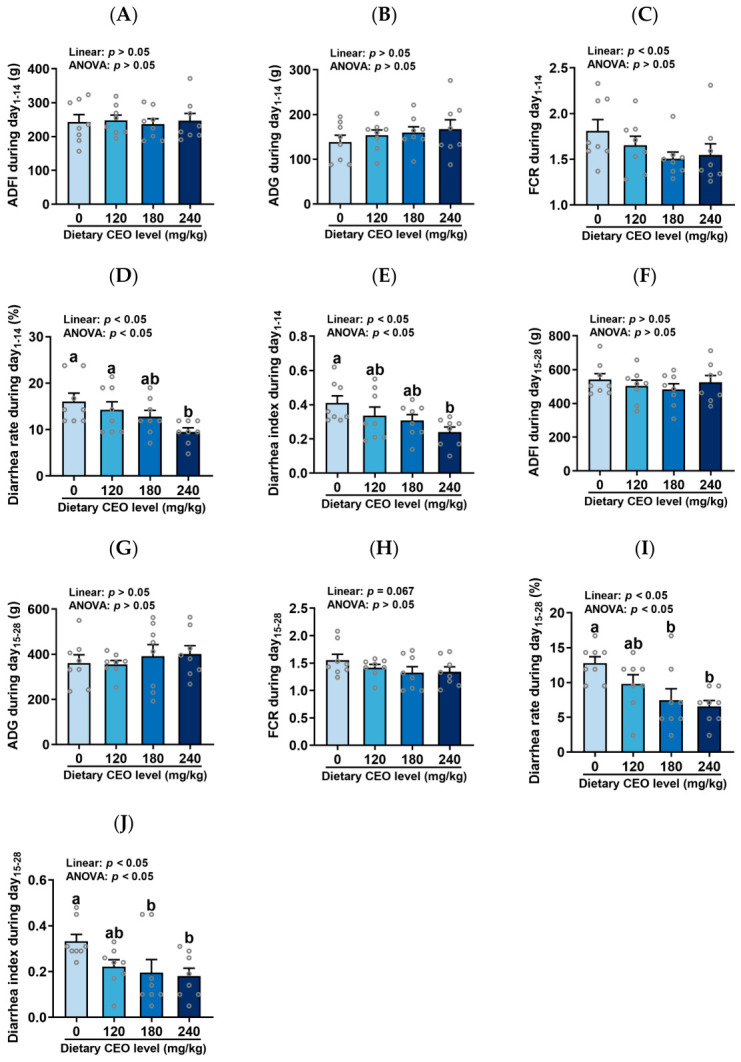
Growth performance of weaned piglets fed diets supplemented with cocrystal essential oil (CEO) (n = 8, mean ± SEM). (**A**–**E**) ADFI, ADG, FCR, diarrhea rate, and diarrhea index during weeks 1–2. (**F**–**J**) ADFI, ADG, FCR, diarrhea rate, and diarrhea index during weeks 3–4. Bars with distinct letters signify significant differences (*p* < 0.05).

The growth performance of weaned piglets fed diets supplemented with cocrystal essential oil (CEO) is shown in Figure 1. Neither ADFI nor ADG was affected by dietary CEO supplementation (*p* > 0.05). However, FCR during weeks 1–2 decreased linearly with increasing dietary CEO levels (*p* < 0.05), while FCR during weeks 3–4 showed a tendency for linear decrease (*p* = 0.067). In addition, dietary supplementation with CEO linearly reduced both the diarrhea rate and diarrhea index during weeks 1–2 and weeks 3–4 (*p* < 0.05). During weeks 1–2, the 240 mg/kg CEO group exhibited a lower diarrhea rate and diarrhea index compared to the control group (without CEO supplementation). During weeks 3–4, both the 180 and 240 mg/kg CEO groups demonstrated a reduced diarrhea rate and diarrhea index relative to the control group (*p* < 0.05). Growth performance parameters did not differ statistically between the 180 and 240 mg/kg CEO groups (*p* > 0.05).

### 3.2. Apparent Total Tract Digestibility (ATTD)

**Figure 2:**
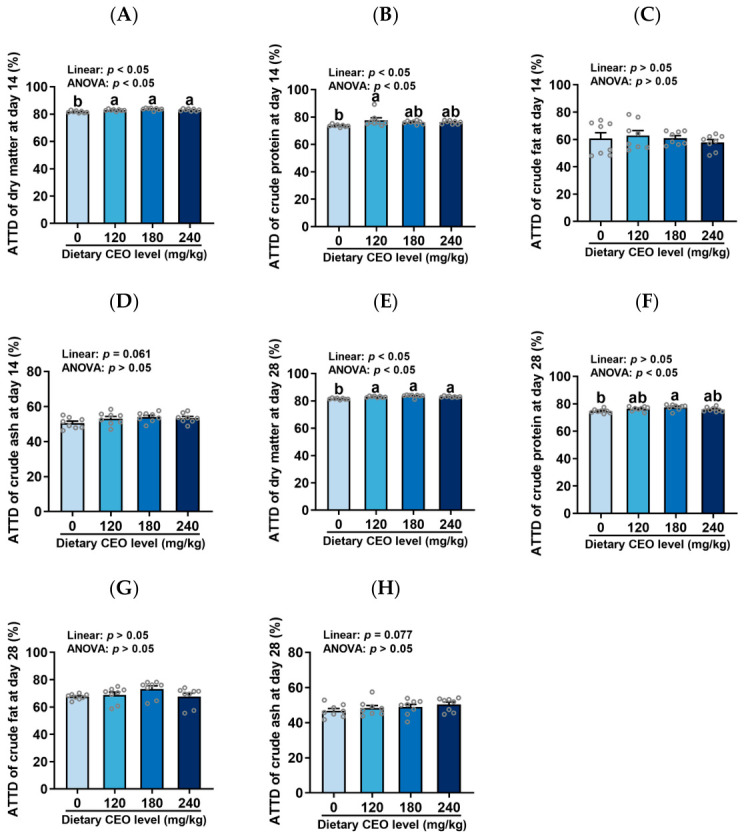
Apparent total tract digestibility (ATTD) of weaned piglets fed CEO-supplemented diets (n = 8, mean ± SEM). (**A**–**D**) ATTD at day 14. (**E**–**H**) ATTD at day 28. Bars with distinct letters signify significant differences (*p* < 0.05).

As shown in Figure 2, CEO supplementation linearly increased the ATTD of dry matter and crude protein (*p* < 0.05) and tended to linearly increase the ATTD of crude fat at day 14 of the experiment (*p* = 0.061). At day 28 of the study, CEO supplementation also linearly increased the ATTD of dry matter (*p* < 0.05) and tended to linearly increase the ATTD of crude ash (*p* = 0.077). Relative to the control group, the ATTD of dry matter was elevated in piglets fed diets supplemented with 120, 180, and 240 mg/kg CEO at both day 14 and day 28 (*p* < 0.05). In addition, the ATTD of crude protein was higher in the 120 mg/kg CEO group at day 14, as well as in the 180 mg/kg CEO group at day 28 (*p* < 0.05). No statistical differences in ATTD were found between the 180 and 240 mg/kg CEO groups (*p* > 0.05).

### 3.3. Intestinal Morphology

**Figure 3:**
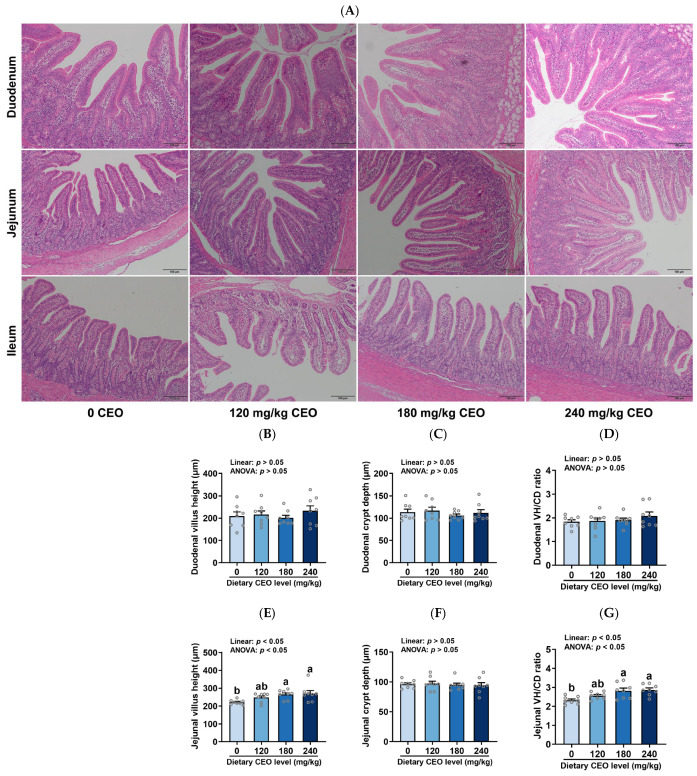

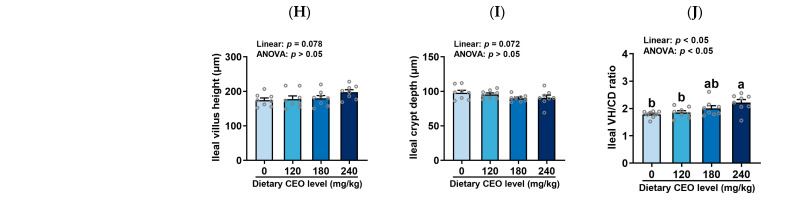
Intestinal morphology of weaned piglets fed CEO-supplemented diets (n = 8, mean ± SEM). (**A**) H&E images (scale bar = 100 μm). (**B**–**D**) Duodenal morphological parameters. (**E**–**G**) Jejunal morphological parameters. (**H**–**J**) Ileal morphological parameters. Bars with distinct letters signify significant differences (*p* < 0.05).

The intestinal morphology of weaned piglets fed CEO-supplemented diets is presented in Figure 3. Duodenal morphology was unaffected by dietary treatments (*p* > 0.05). However, dietary CEO supplementation linearly increased jejunal villus height (VH) and the villus height/crypt depth (VH/CD) ratio (*p* < 0.05). For the ileum, CEO supplementation also linearly increased the VH/CD ratio (*p* < 0.05) and tended to linearly increase villus height (*p* = 0.078) while decreasing crypt depth (*p* = 0.072). Compared with the control group, supplementation with 180 and 240 mg/kg CEO increased jejunal villus height and the VH/CD ratio (*p* < 0.05). Additionally, 240 mg/kg CEO supplementation elevated the ileal VH/CD ratio relative to the control group (*p* < 0.05). Intestinal morphology parameters were not significantly different between the 180 and 240 mg/kg CEO groups (*p* > 0.05).

### 3.4. Antioxidant Status

**Figure 4:**
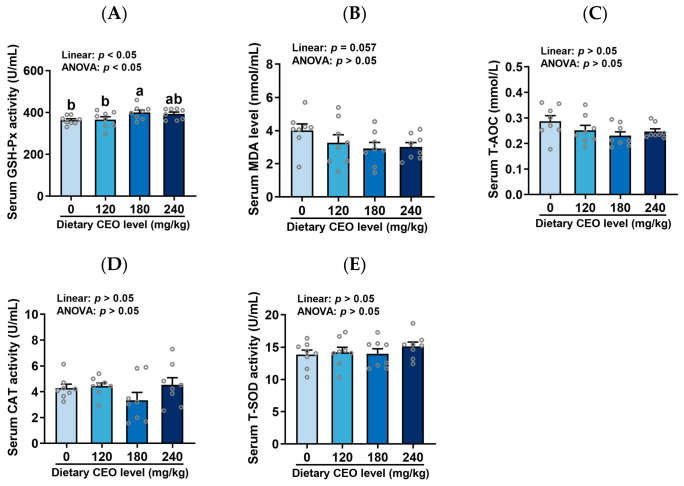
Serum antioxidant status of weaned piglets fed CEO-supplemented diets (n = 8, mean ± SEM). (**A**–**E**) GSH-Px activity, MDA level, T-AOC, CAT activity, and T-SOD activity. Bars with distinct letters signify significant differences (*p* < 0.05).

Figure 4 shows the serum antioxidant status of weaned piglets fed CEO-supplemented diets. Serum T-AOC, CAT activity, and T-SOD activity were unaffected by dietary treatments (*p* > 0.05). However, CEO supplementation linearly increased serum GSH-Px activity (*p* < 0.05) and tended to linearly decrease serum MDA level (*p* = 0.057). Relative to the control group, 180 mg/kg CEO supplementation elevated serum GSH-Px activity in piglets (*p* < 0.05). Additionally, serum antioxidant indices did not differ statistically between the 180 and 240 mg/kg CEO groups (*p* > 0.05).

## 4. Discussion

Early-weaned piglets have immature gastrointestinal and antioxidant functions [30], and face multiple challenges, such as the transition from liquid sow milk to solid feed, environmental changes, and increased pathogen infection risks [2]. These weaning stress factors contribute to low nutrient digestibility, impaired intestinal morphology, oxidative stress, and diarrhea, thereby compromising the health and growth performance of weaned piglets [5,6]. Therefore, the present study aimed to evaluate the impacts of dietary inclusion with different doses of CEO on growth performance, diarrhea incidence, nutrient digestibility, intestinal morphology, and antioxidant status in weaned piglets. A previous study had reported that essential oils may decrease feed intake due to their pungent odor, which compromises feed palatability and acceptability to animals [31]. However, in our study, dietary addition with CEO did not influence feed intake in piglets. This discrepancy may be attributed to the fact that the cocrystallization process effectively reduced the irritating smell of the essential oils. To date, no studies have reported the effects of CEO in weaned piglets. Nevertheless, relevant research has been conducted in broilers. Yang et al. (2025) found that dietary addition with 80 mg/kg CEO increased ADFI, along with improved ADG and reduced FCR in broilers [19]. Additionally, in finishing pigs, Ma et al. (2026) reported that dietary addition with 100 mg/kg CEO increased ADG and reduced FCR without affecting ADFI [21]. Regarding FCR, our results were consistent with these findings. FCR during weeks 1–2 decreased linearly with increasing dietary CEO levels, while FCR during weeks 3–4 showed a tendency for linear decrease. More importantly, dietary supplementation with CEO linearly reduced both the diarrhea rate and diarrhea index during weeks 1–2 and weeks 3–4. Therefore, dietary addition with CEO has potential benefits for improving the growth performance of weaned piglets.

The improvement in feed conversion efficiency of weaned piglets by CEO may be associated with its effects on nutrient digestibility. Therefore, we subsequently determined the total tract nutrient digestibility (ATTD) in piglets. In this study, we observed that dietary CEO inclusion linearly elevated the ATTD of dry matter and crude protein and tended to linearly increase the ATTD of crude fat on day 14 of the experiment. On day 28 of the study, CEO supplementation also linearly increased the ATTD of dry matter and tended to linearly increase the ATTD of crude ash. Compared with the control group, the ATTD of dry matter was elevated in piglets fed diets supplemented with 120, 180, and 240 mg/kg CEO on both day 14 and day 28. Additionally, the ATTD of crude protein was higher in the 120 mg/kg CEO group on day 14, as well as in the 180 mg/kg CEO group on day 28. Similarly, Huang et al. (2010) reported that dietary inclusion with a blend of essential oils (main bioactive components: cinnamaldehyde, carvacrol, thymol, and eugenol) at a dosage of 1000 mg/kg improved the digestibility of dry matter and nitrogen in weaned piglets [32]. Chen et al. (2023) also observed that dietary addition with 250, 500, and 1000 mg/kg *Litsea cubeba* essential oil linearly increased the digestibility of dry matter and crude protein in *Taoyuan* black pigs [33]. The above studies support our findings that dietary CEO supplementation improved nutrient digestibility in piglets. Notably, although Huang et al. (2010) [32] and Chen et al. (2023) [33] also reported enhanced nutrient digestibility in pigs, their supplementation dosages were much higher than those employed in our experiment. This may be attributed to the cocrystal technology processing of essential oils in our trial, which can enhance their stability and bioavailability [16]. However, no statistical differences for the ATTD of piglets were observed between the 180 and 240 mg/kg groups. Therefore, our results suggest that 180 mg/kg CEO can be recommended for weaned piglets in terms of nutrient digestibility.

The small intestine is the primary place for nutrient digestion and absorption in piglets [34]. Intestinal villus height (VH), crypt depth (CD), and the villus height to crypt depth (VH/CD) ratio are commonly used criteria for assessing intestinal health and function, including nutrient digestibility [30]. Therefore, we evaluated the small intestinal morphology of piglets in this study. In our study, dietary CEO inclusion linearly increased jejunal villus height and the VH/CD ratio. For the ileum, CEO supplementation also linearly increased the VH/CD ratio and tended to linearly increase villus height while decreasing crypt depth. These findings are consistent with those of Yang et al. (2025), who reported that dietary addition with 30, 60, and 120 mg/kg CEO linearly increased jejunal villus height and the VH/CD ratio of broilers [19]. Relative to the control group, supplementation with 180 and 240 mg/kg CEO elevated jejunal villus height and the VH/CD ratio. Additionally, 240 mg/kg CEO supplementation elevated the ileal VH/CD ratio compared to the control group. However, no statistical differences for intestinal morphology parameters were observed between the 180 and 240 mg/kg groups. Of note, although nutrient digestibility and intestinal morphology were improved, no significant changes were observed in ADG or ADFI. However, FCR decreased linearly with increasing dietary CEO supplementation, likely attributable to the improvements in digestibility and intestinal morphology. Therefore, based on small intestinal morphology, 180 mg/kg CEO can be recommended for weaned piglets, which is also consistent with the results of nutrient digestibility.

Essential oils are well known for their antioxidant activity [35,36]. Here, we measured serum antioxidant parameters in piglets. In our study, CEO supplementation linearly increased serum GSH-Px activity and tended to linearly decrease serum MDA level. Compared with the control group, 180 mg/kg CEO supplementation elevated serum GSH-Px activity in piglets. GSH-Px, a selenium-dependent antioxidant enzyme, catalyzes the conversion of reduced glutathione (GSH) to oxidized glutathione (GSSG) while reducing hydrogen peroxide to water, thereby protecting cells from oxidative stress [37]. Malondialdehyde (MDA), a by-product of lipid peroxidation, is commonly used as an indicator of oxidative status in animals [38]. These results indicate that dietary CEO inclusion improved the antioxidant status of weaned piglets. Interestingly, CEO supplementation did not alter T-AOC, CAT activity, or T-SOD activity, yet enhanced GSH-Px activity and reduced MDA level. This selective modulation of antioxidant parameters can be attributed to the specific bioactive constituents of CEO, namely thymol and carvacrol. Consistently, Ma et al. (2026) reported that dietary inclusion with 100 mg/kg CEO decreased MDA level in the muscle of finishing pigs [21]. Similarly, Yang et al. (2026) found that dietary inclusion with 60 mg/kg CEO elevated GSH-Px activity and reduced MDA level in the serum of broilers [19]. Li et al. (2024) observed that dietary inclusion with 40 or 60 mg/kg CEO reduced MDA level in the breast muscle of broilers [20]. Collectively, these findings suggest that CEO inclusion can enhance the antioxidant status of weaned piglets.

Lastly, it should be noted that male piglets were selected as the research subjects for small-scale trials to facilitate fecal collection for ATTD determination. However, future research should still be conducted in large-scale commercial pig farms, including mixed-sex populations. Additionally, another limitation of the present study is that gut microbiota was not measured. Given that essential oils often exert antimicrobial effects, the observed reduction in diarrhea in piglets may also be attributed to the modulatory effects of CEO on gut microbiota, which warrants further investigation in future studies.

## 5. Conclusions

In conclusion, the dietary incorporation of 180 mg/kg CEO is recommended for weaned piglets, considering its multifaceted benefits in mitigating diarrhea, optimizing nutrient digestibility, ameliorating intestinal morphology, and fortifying antioxidant capacity. CEO is a promising feed additive for early-weaned piglets.

## Figures and Tables

**Table 1:** Formulation and nutritional contents of the basal diet.

Ingredient	Content (%)	Nutrient	Content ^2^
Corn	37.30	Metabolizable energy, kcal/kg	3311
Extruded corn	20.00	Crude protein, %	19.07
Extruded soybean	10.00	Lysine, %	1.39
Fermented soybean meal	6.50	Methionine, %	0.48
Whey powder	5.00	Threonine, %	0.79
Wheat flour	5.00	Tryptophan, %	0.24
Fish meal	5.00		
Soy protein concentrate	4.00		
Wheat bran	4.00		
Limestone	0.70		
Dicalcium phosphate	0.50		
Salt	0.30		
Choline chloride	0.12		
L-Lysine hydrochloride	0.50		
DL-Methionine	0.16		
L-Valine	0.14		
L-Threonine	0.10		
L-Tryptophan	0.03		
Premix ^1^	0.65		
Total	100.00		

^1^ The premix provides the following nutritional composition per kilogram of feed: vitamin A, 10,000 IU; vitamin D_3_, 1000 IU; vitamin E, 60 IU; vitamin K_3_, 1.5 mg; vitamin B_1_, 1 mg; vitamin B_2_, 4 mg; vitamin B_6_, 3 mg; vitamin B_12_, 0.3 mg; niacin, 30 mg; pantothenic acid, 5 mg; folic acid, 1.3 mg; biotin, 0.3 mg; Fe, 100 mg; Cu, 6 mg; Mn, 4 mg; Zn, 150 mg; I, 0.14 mg; Se, 0.3 mg. ^2^ Calculated values.

## Data Availability

Data will be made available upon request, and the original intestinal H&E images are provided in the Supplementary Materials.

## Supplementary Materials

The following supporting information can be downloaded at: https://www.mdpi.com/article/10.3390/ani16091400/s1, File S1: Original intestinal H&E images.

## Abbreviations

ADFI	Average daily feed intake
ADG	Average daily gain
ATTD	Apparent total tract digestibility
CAT	Catalase
CEO	Cocrystal essential oil
FCR	Feed conversion ratio
GSH-Px	Glutathione peroxidase
H&E	Hematoxylin and eosin
MDA	Malondialdehyde
T-AOC	Total antioxidant capacity
T-SOD	Total superoxide dismutase
VH/CD	Villus height/crypt depth
