# Supplementary figures and images for: Impact of Dietary Inclusion with Cocrystal Essential Oil on Growth Performance, Nutrient Digestibility, Intestinal Morphology, and Antioxidant Status in Weaned Piglets
Source: Animals (Basel). 2026 May 3;16(9):1400. doi: 10.3390/ani16091400 (PMC13163102; doi:10.3390/ani16091400)

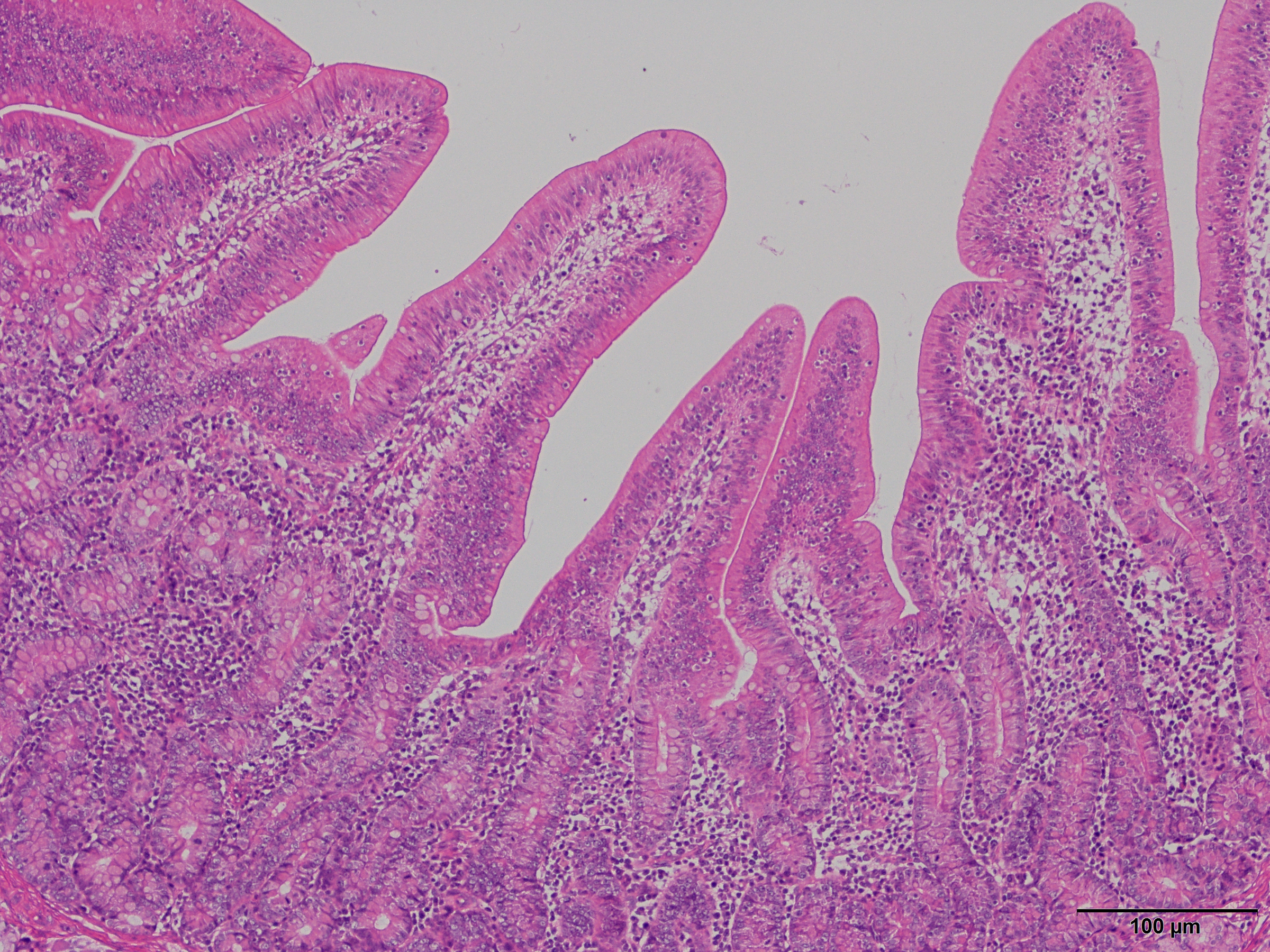

Supplement: Supplementary file 1 [file animals-16-01400-s001.zip › 1. Duodenum/0 CEO group/Duodenum-1-1-Figure 3A.jpg]

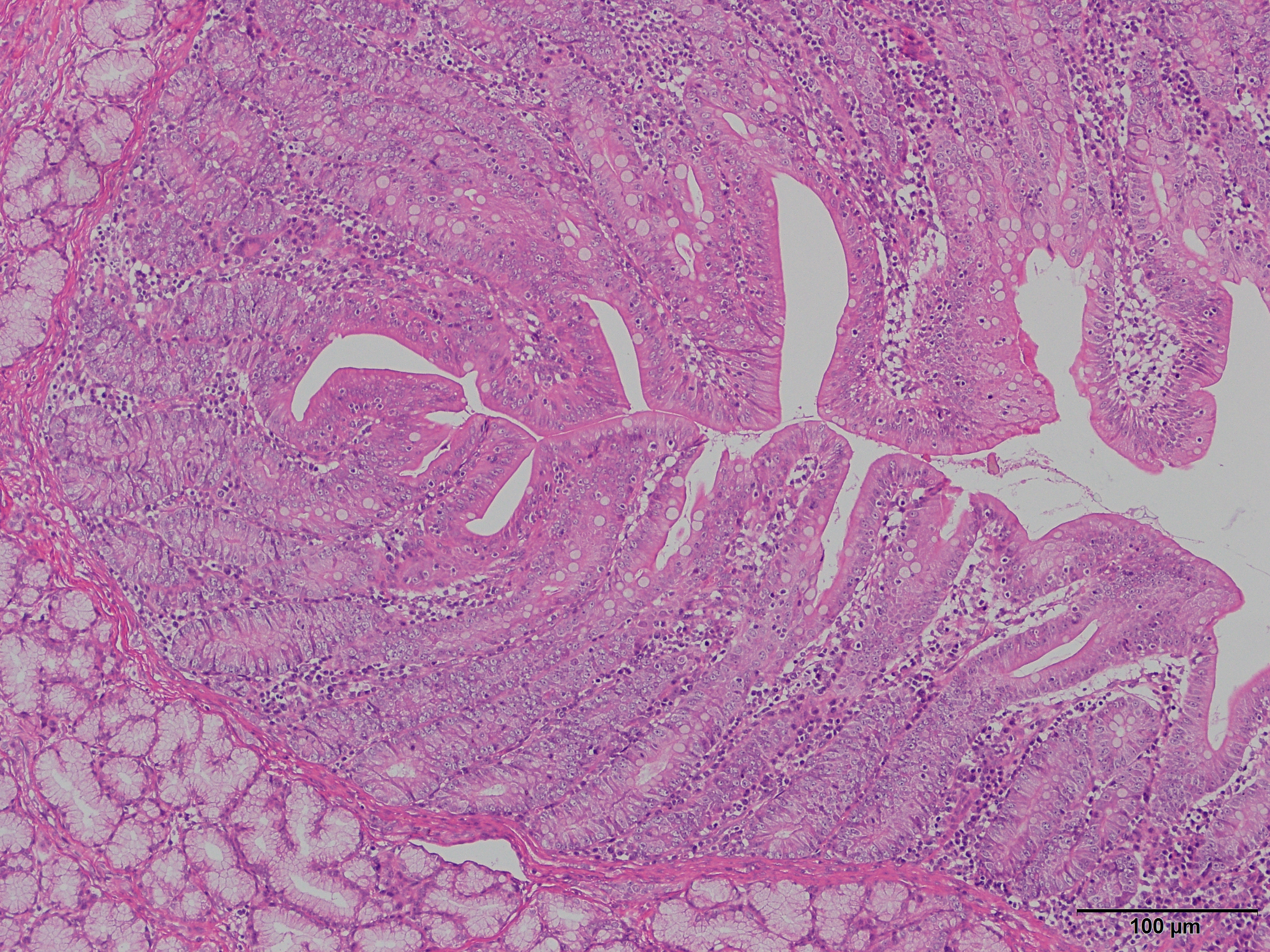

Supplement: Supplementary file 1 [file animals-16-01400-s001.zip › 1. Duodenum/0 CEO group/Duodenum-1-2.jpg]

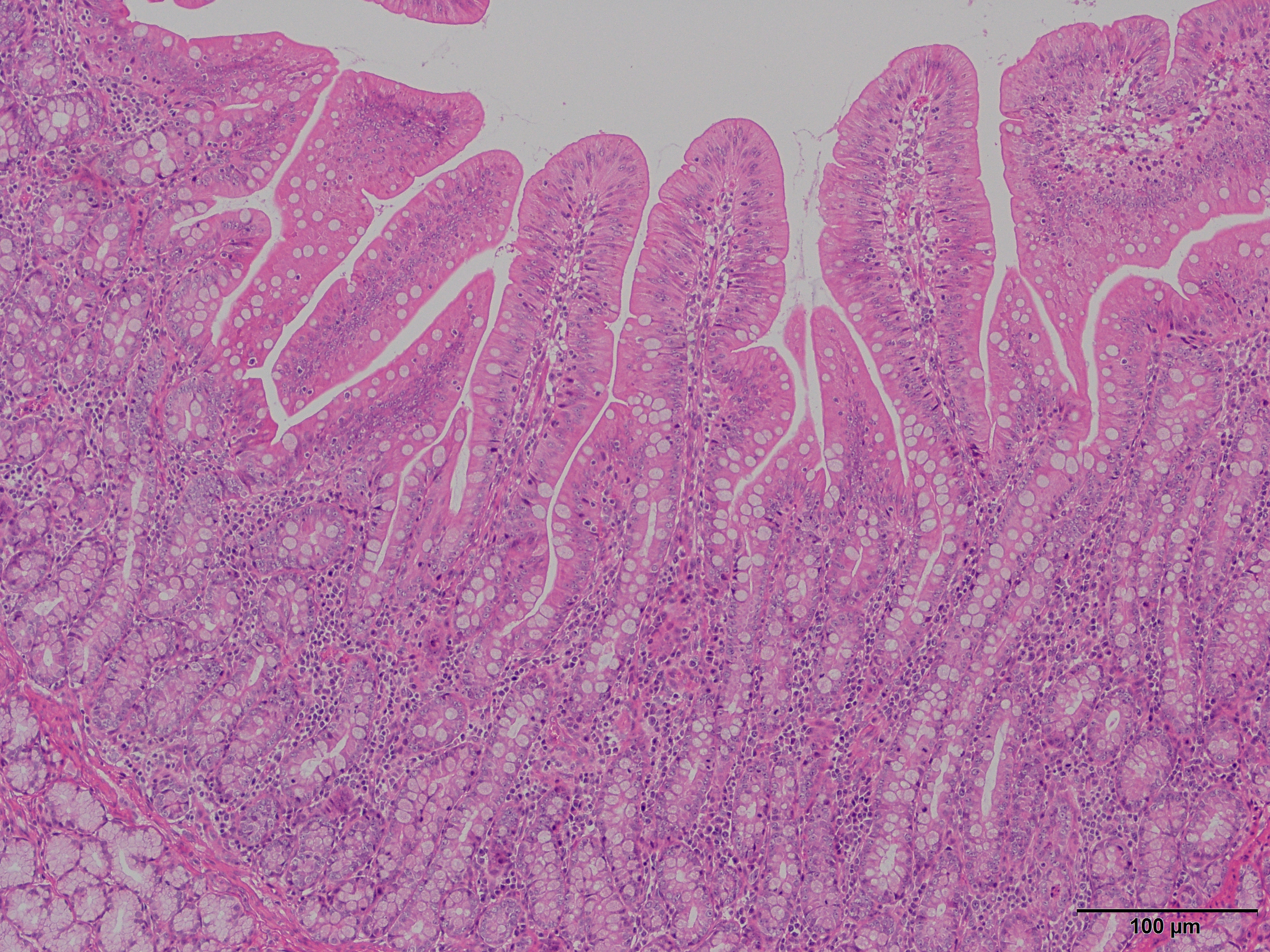

Supplement: Supplementary file 1 [file animals-16-01400-s001.zip › 1. Duodenum/0 CEO group/Duodenum-1-3.jpg]

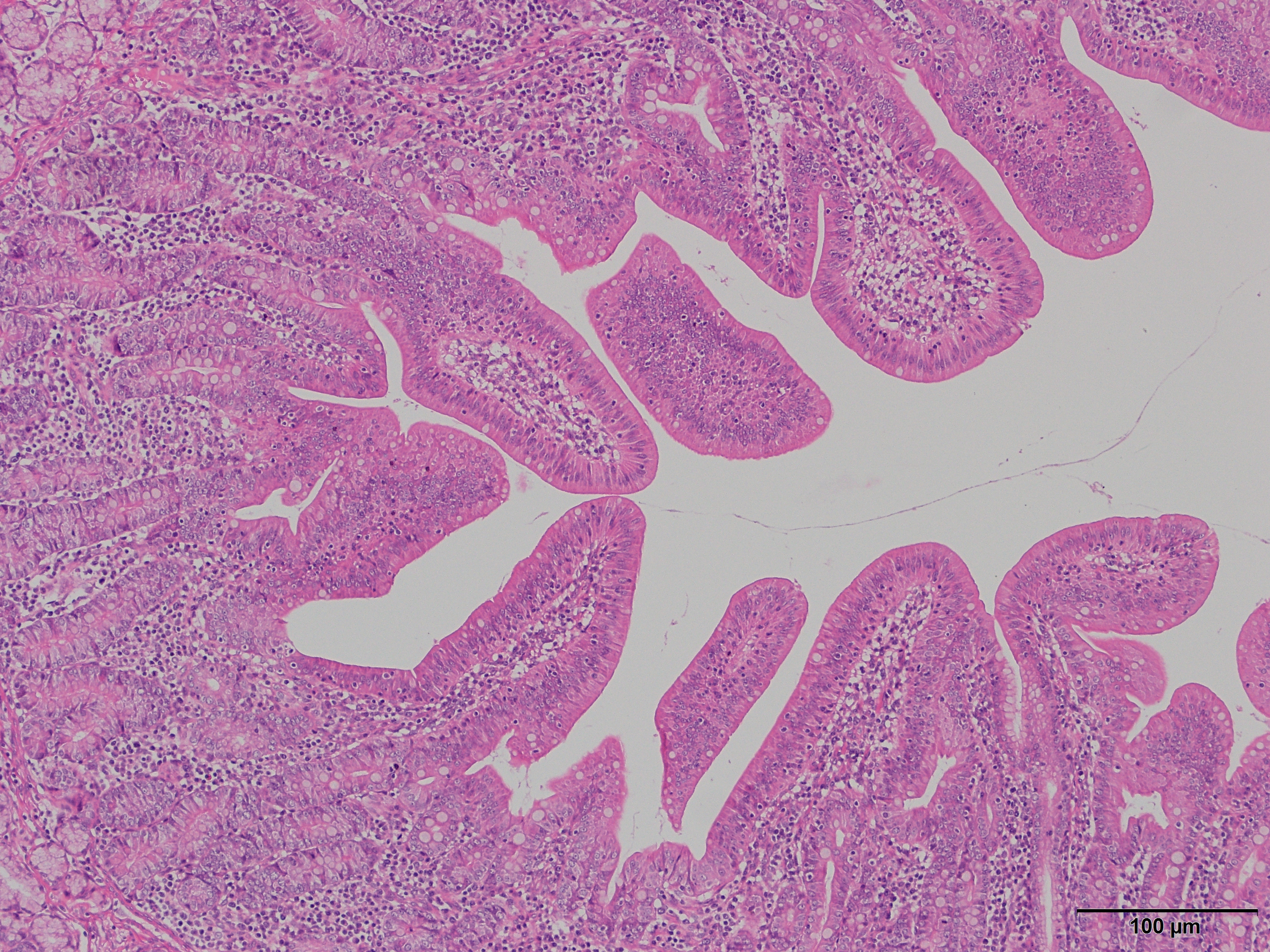

Supplement: Supplementary file 1 [file animals-16-01400-s001.zip › 1. Duodenum/0 CEO group/Duodenum-1-4.jpg]

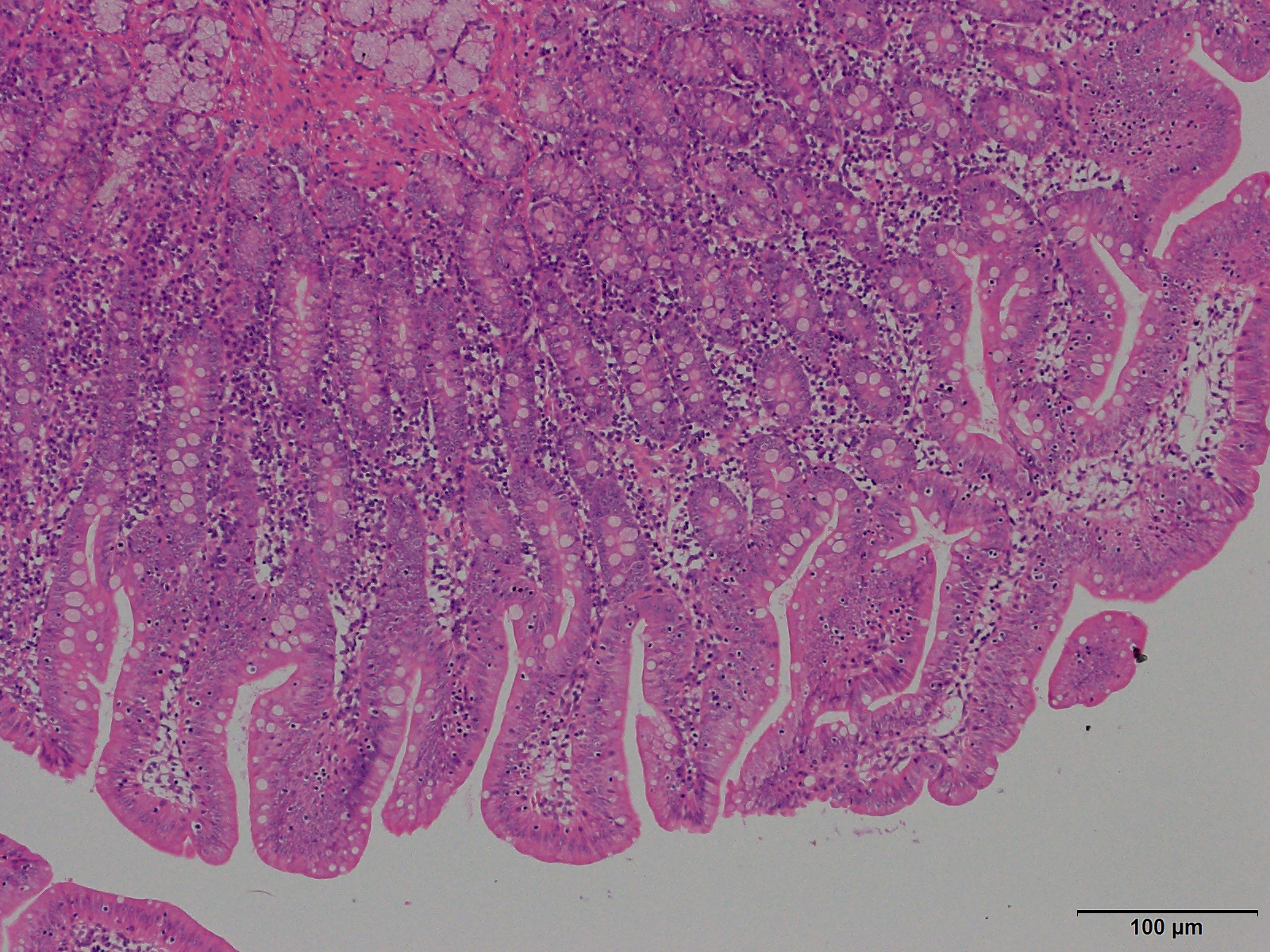

Supplement: Supplementary file 1 [file animals-16-01400-s001.zip › 1. Duodenum/0 CEO group/Duodenum-1-5.jpg]

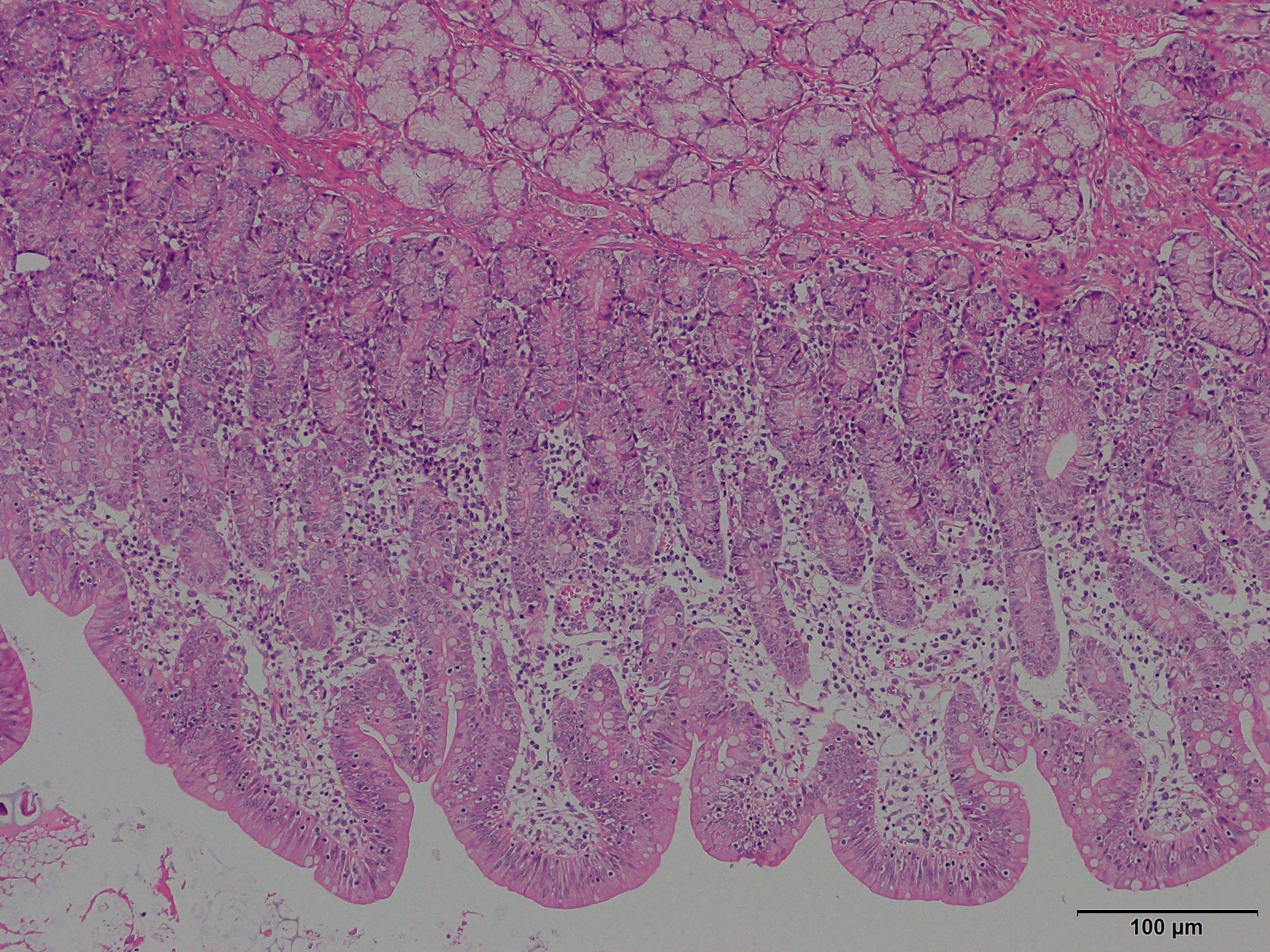

Supplement: Supplementary file 1 [file animals-16-01400-s001.zip › 1. Duodenum/0 CEO group/Duodenum-1-6.jpg]

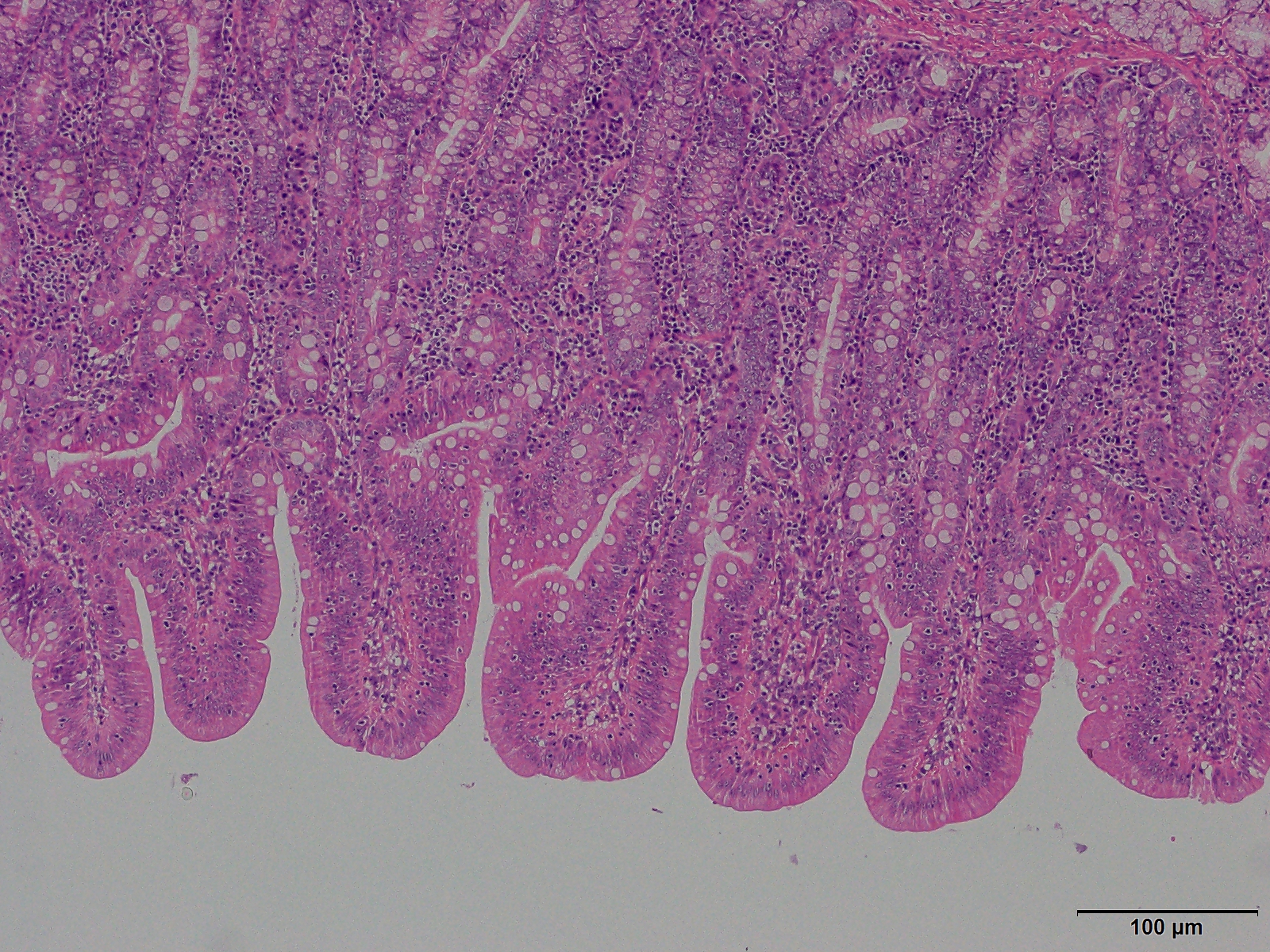

Supplement: Supplementary file 1 [file animals-16-01400-s001.zip › 1. Duodenum/0 CEO group/Duodenum-1-7.jpg]

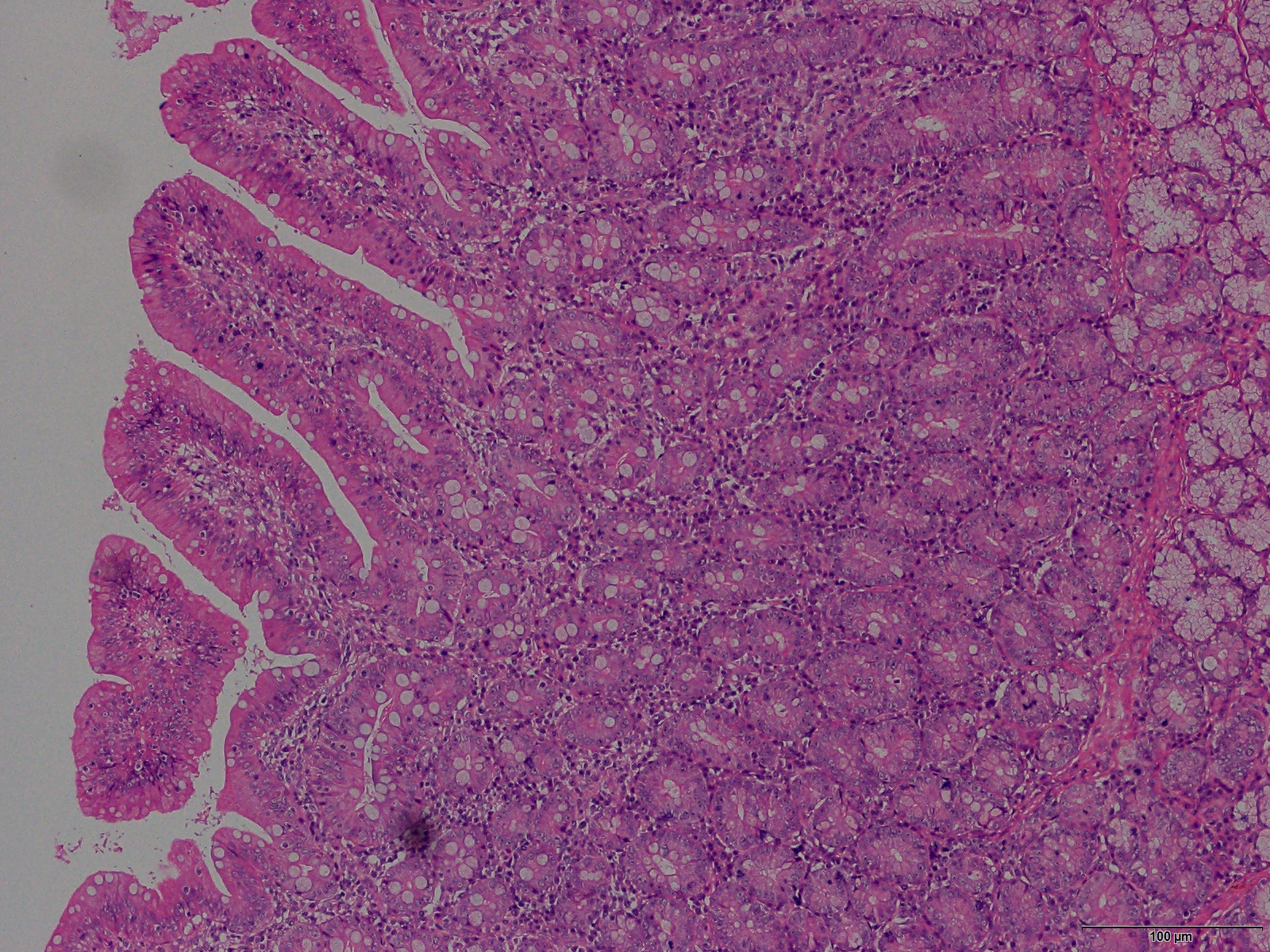

Supplement: Supplementary file 1 [file animals-16-01400-s001.zip › 1. Duodenum/0 CEO group/Duodenum-1-8.jpg]

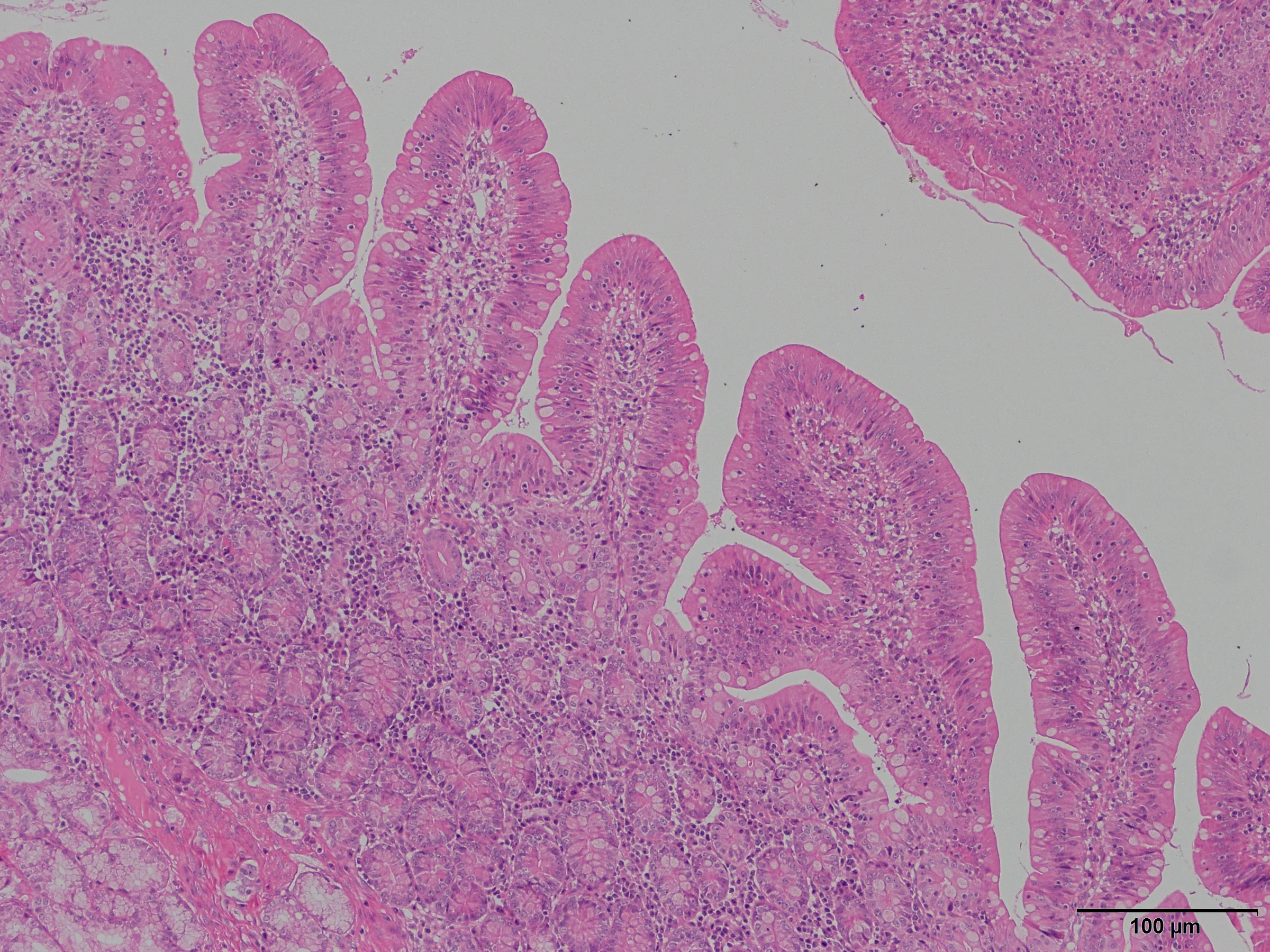

Supplement: Supplementary file 1 [file animals-16-01400-s001.zip › 1. Duodenum/120 mg kg CEO group/Duodenum-2-1.webp]

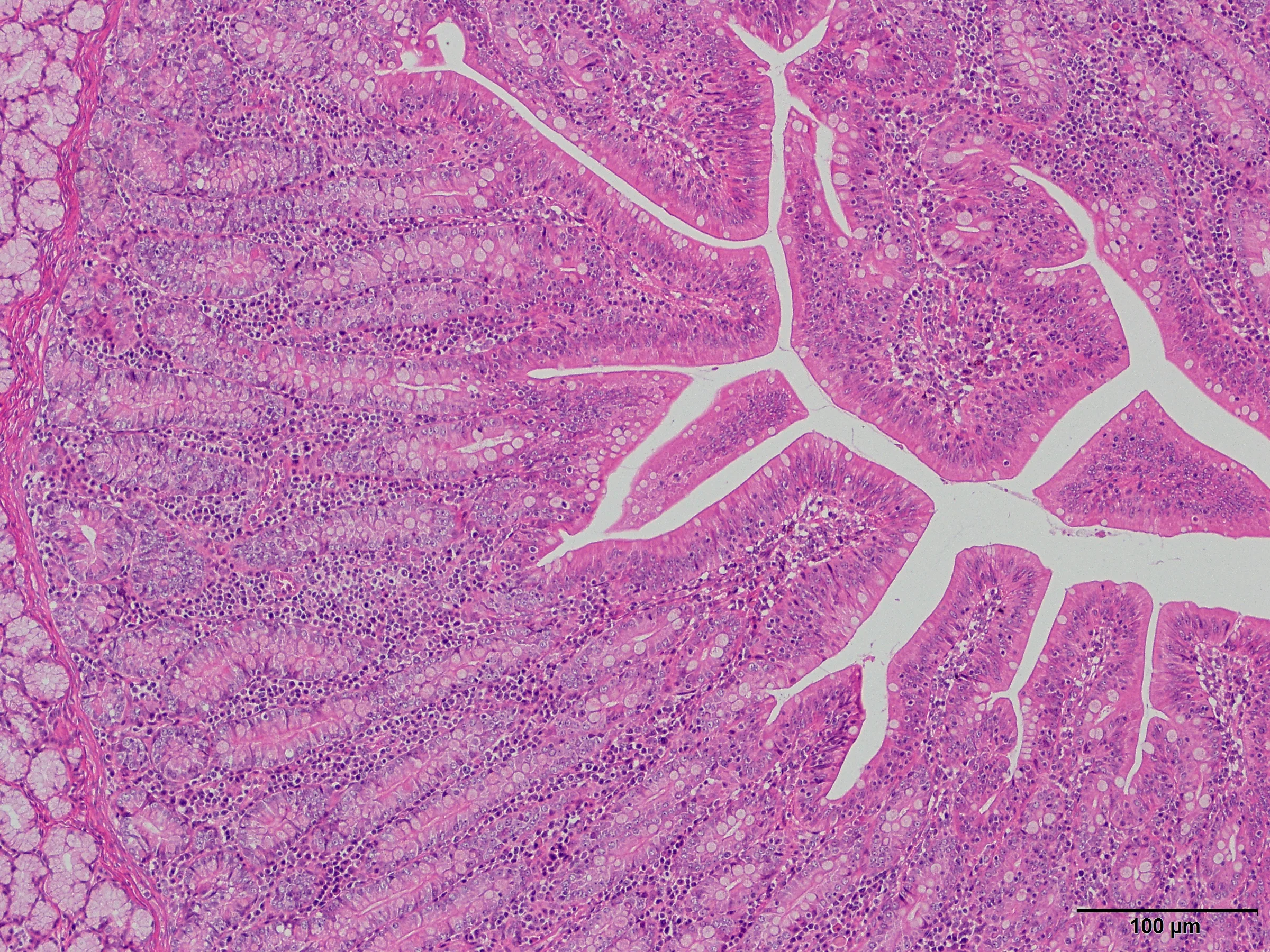

Supplement: Supplementary file 1 [file animals-16-01400-s001.zip › 1. Duodenum/120 mg kg CEO group/Duodenum-2-2.webp]

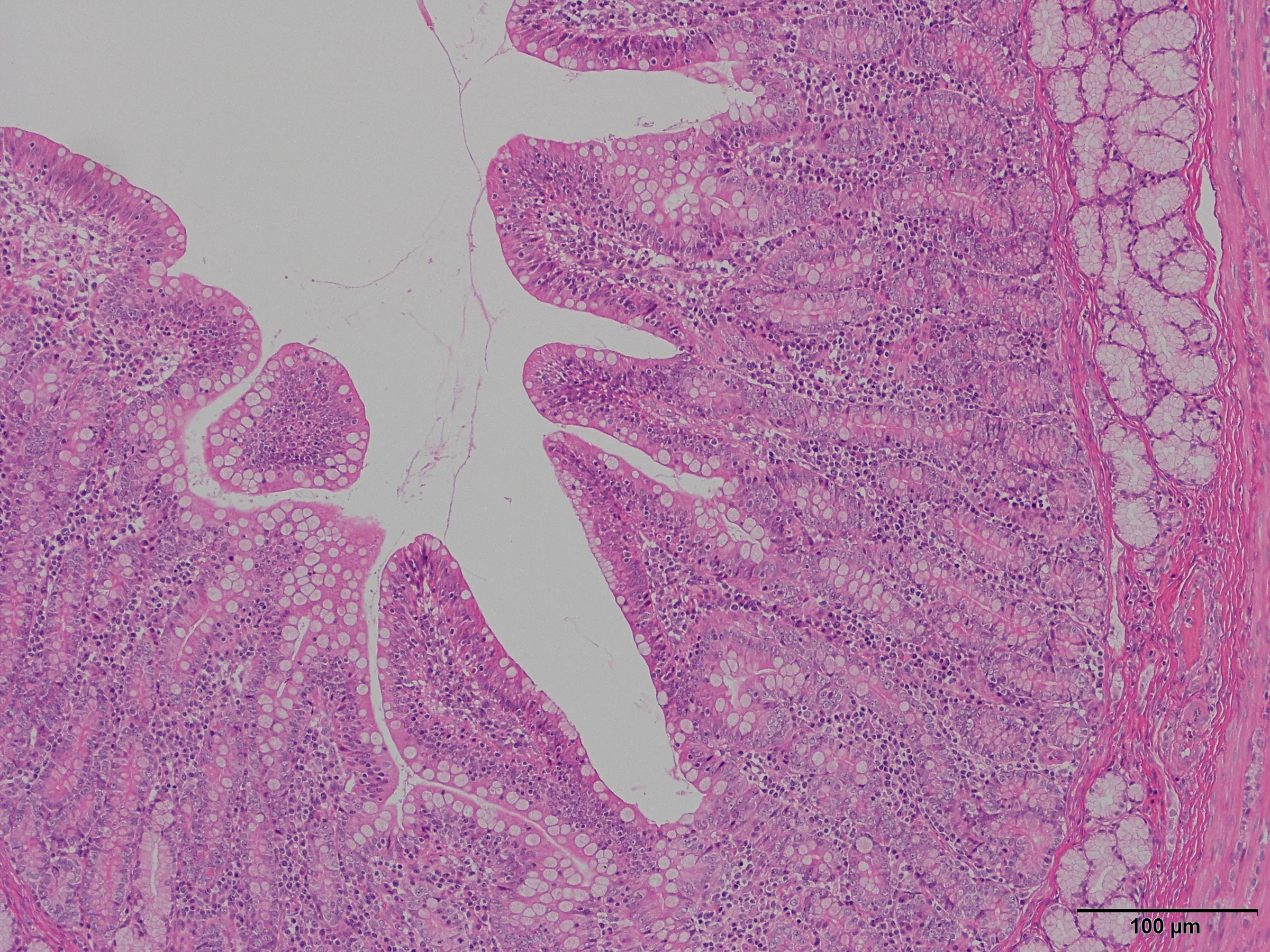

Supplement: Supplementary file 1 [file animals-16-01400-s001.zip › 1. Duodenum/120 mg kg CEO group/Duodenum-2-3.webp]

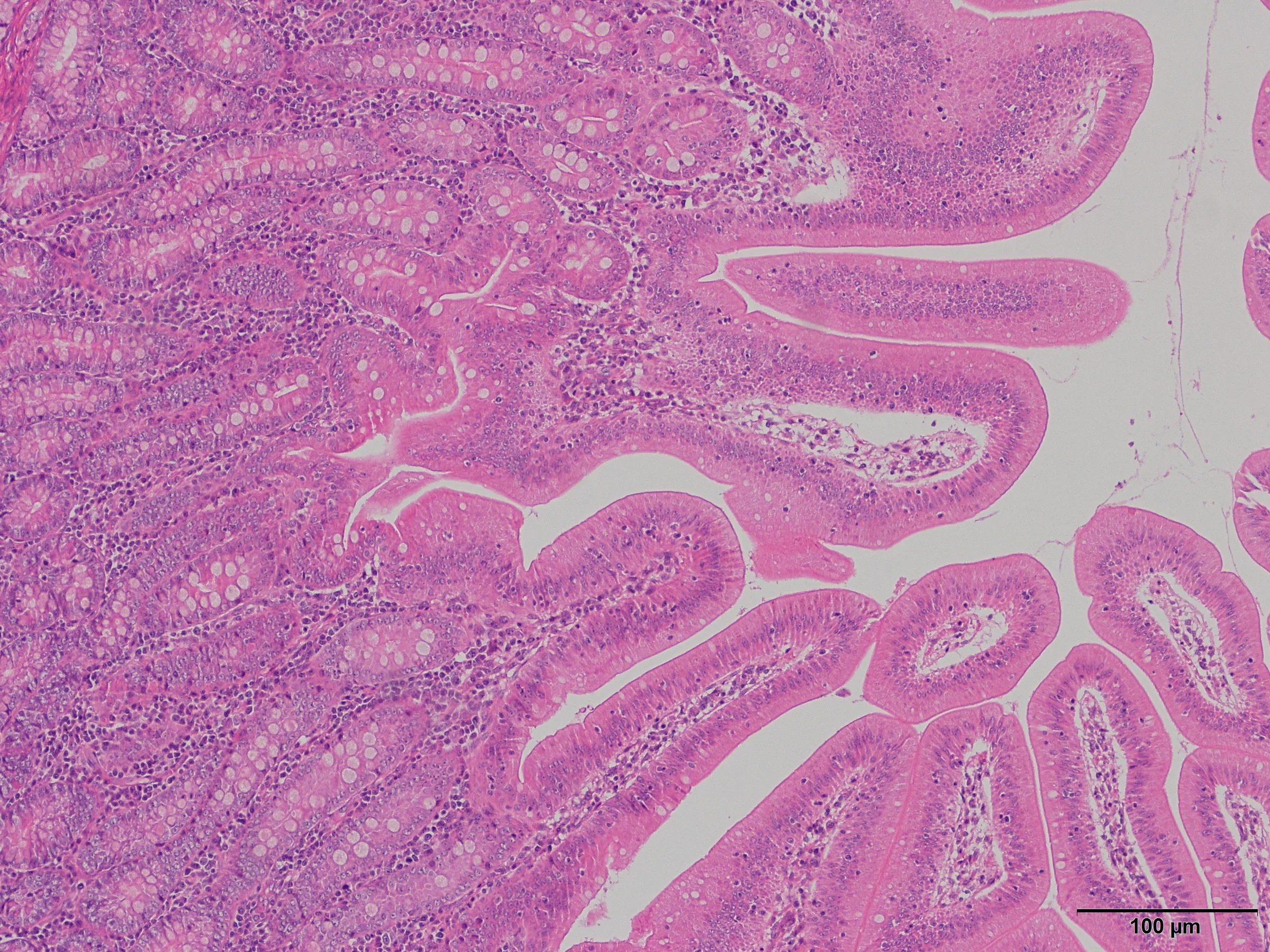

Supplement: Supplementary file 1 [file animals-16-01400-s001.zip › 1. Duodenum/120 mg kg CEO group/Duodenum-2-4.webp]

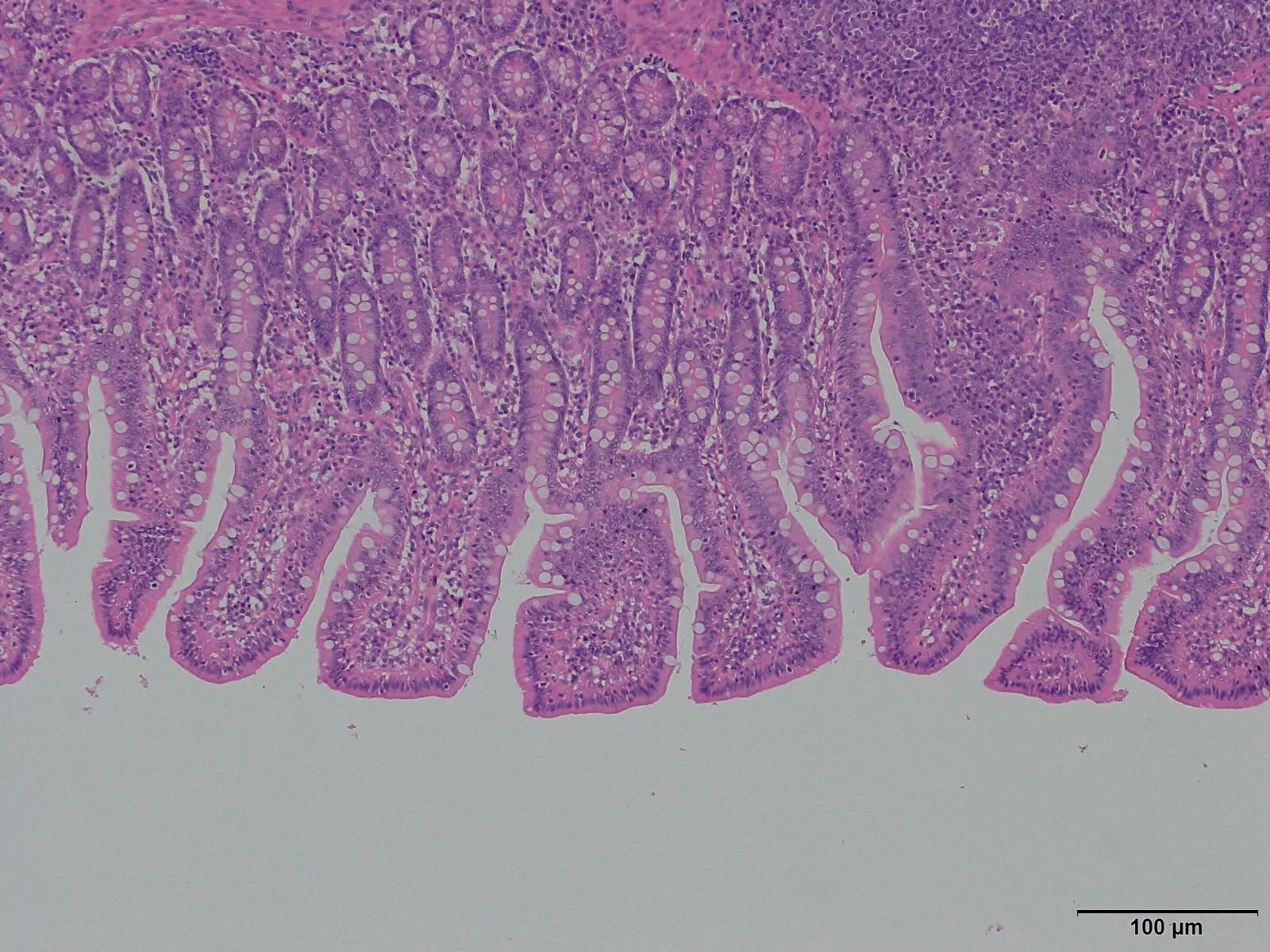

Supplement: Supplementary file 1 [file animals-16-01400-s001.zip › 1. Duodenum/120 mg kg CEO group/Duodenum-2-5.webp]

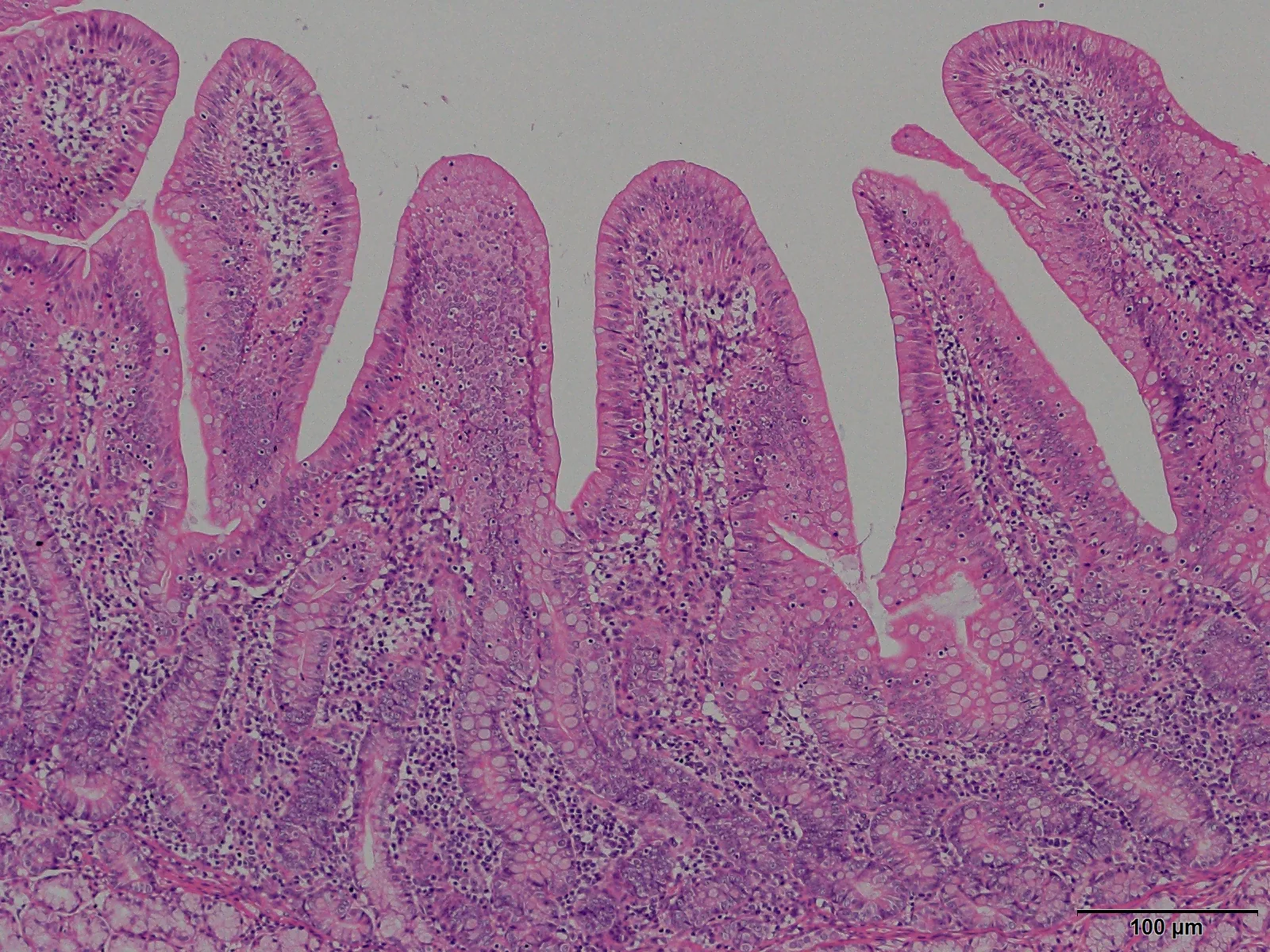

Supplement: Supplementary file 1 [file animals-16-01400-s001.zip › 1. Duodenum/120 mg kg CEO group/Duodenum-2-6.webp]

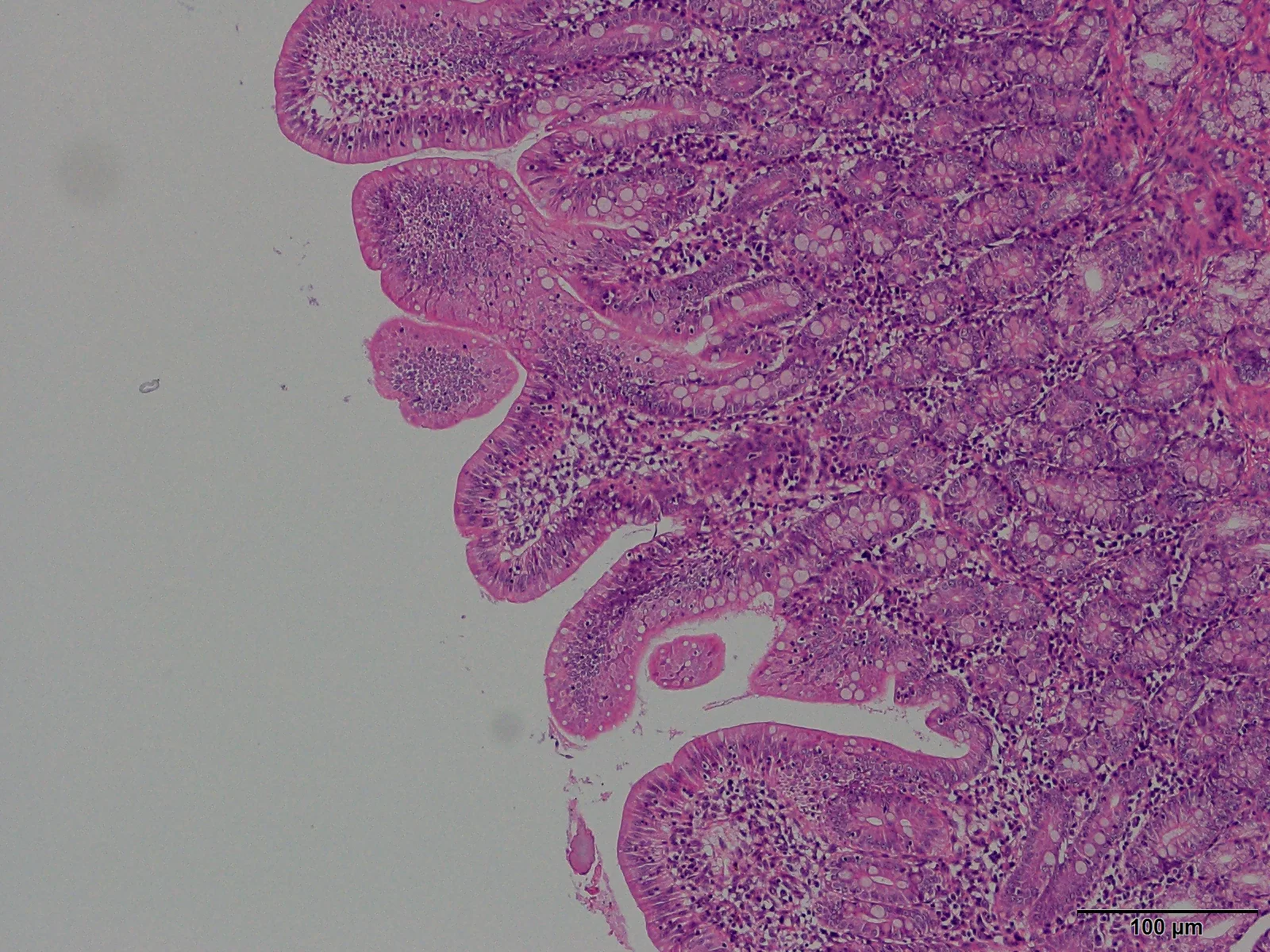

Supplement: Supplementary file 1 [file animals-16-01400-s001.zip › 1. Duodenum/120 mg kg CEO group/Duodenum-2-7.webp]

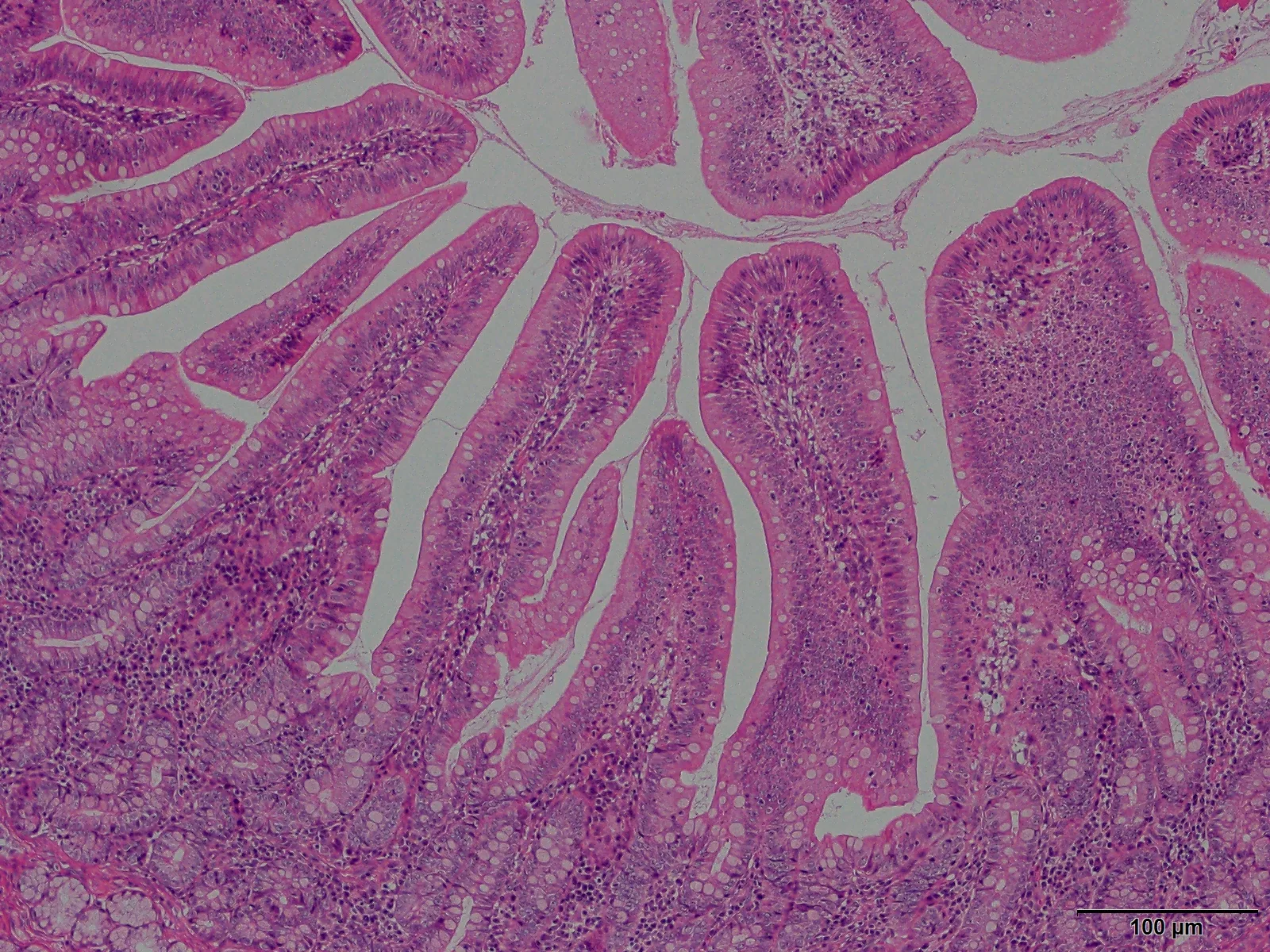

Supplement: Supplementary file 1 [file animals-16-01400-s001.zip › 1. Duodenum/120 mg kg CEO group/Duodenum-2-8-Figure 3A.webp]

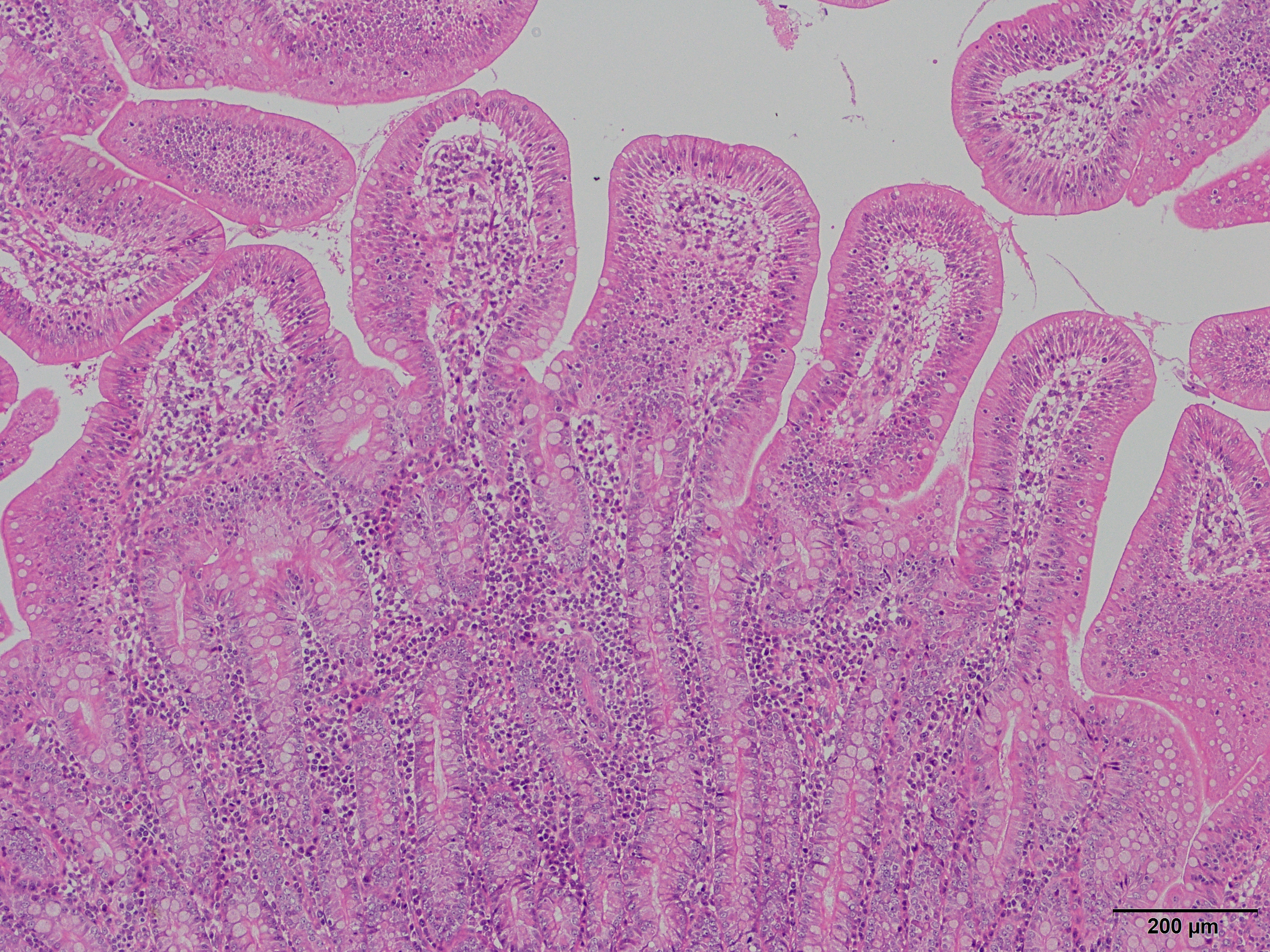

Supplement: Supplementary file 1 [file animals-16-01400-s001.zip › 1. Duodenum/180 mg kg CEO group/Duodenum-3-1.jpg]

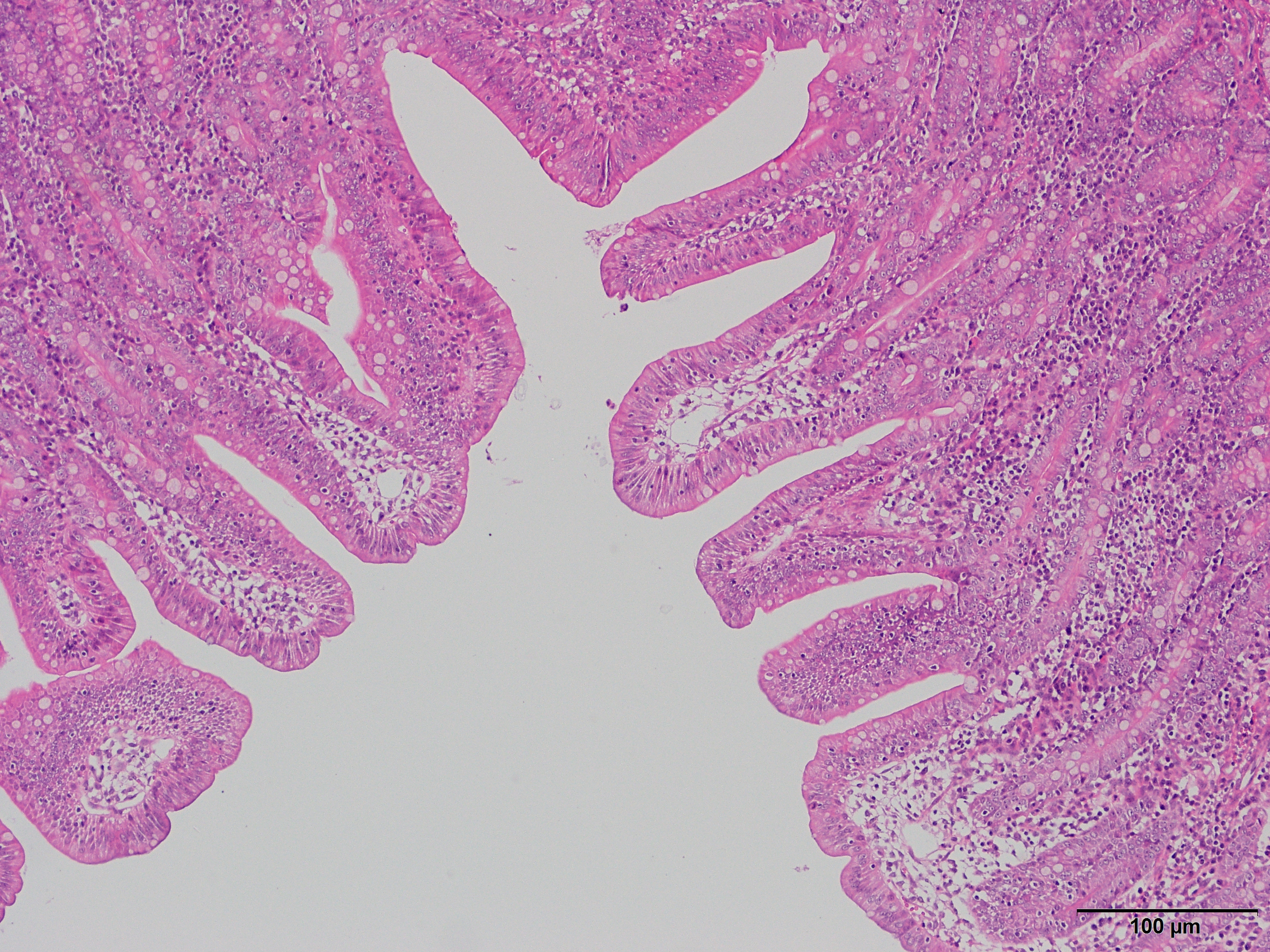

Supplement: Supplementary file 1 [file animals-16-01400-s001.zip › 1. Duodenum/180 mg kg CEO group/Duodenum-3-2.jpg]

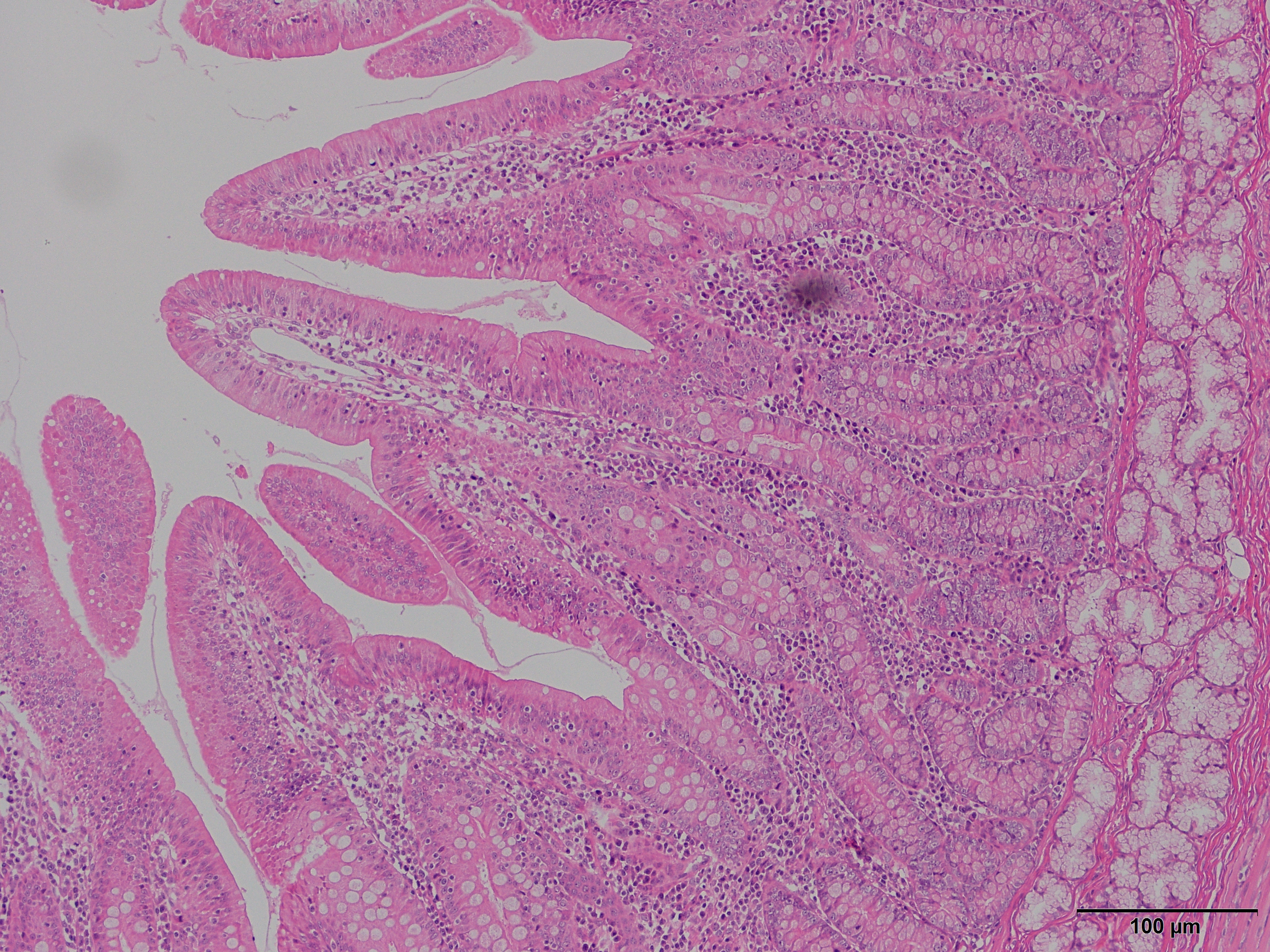

Supplement: Supplementary file 1 [file animals-16-01400-s001.zip › 1. Duodenum/180 mg kg CEO group/Duodenum-3-3-Figure 3A.jpg]

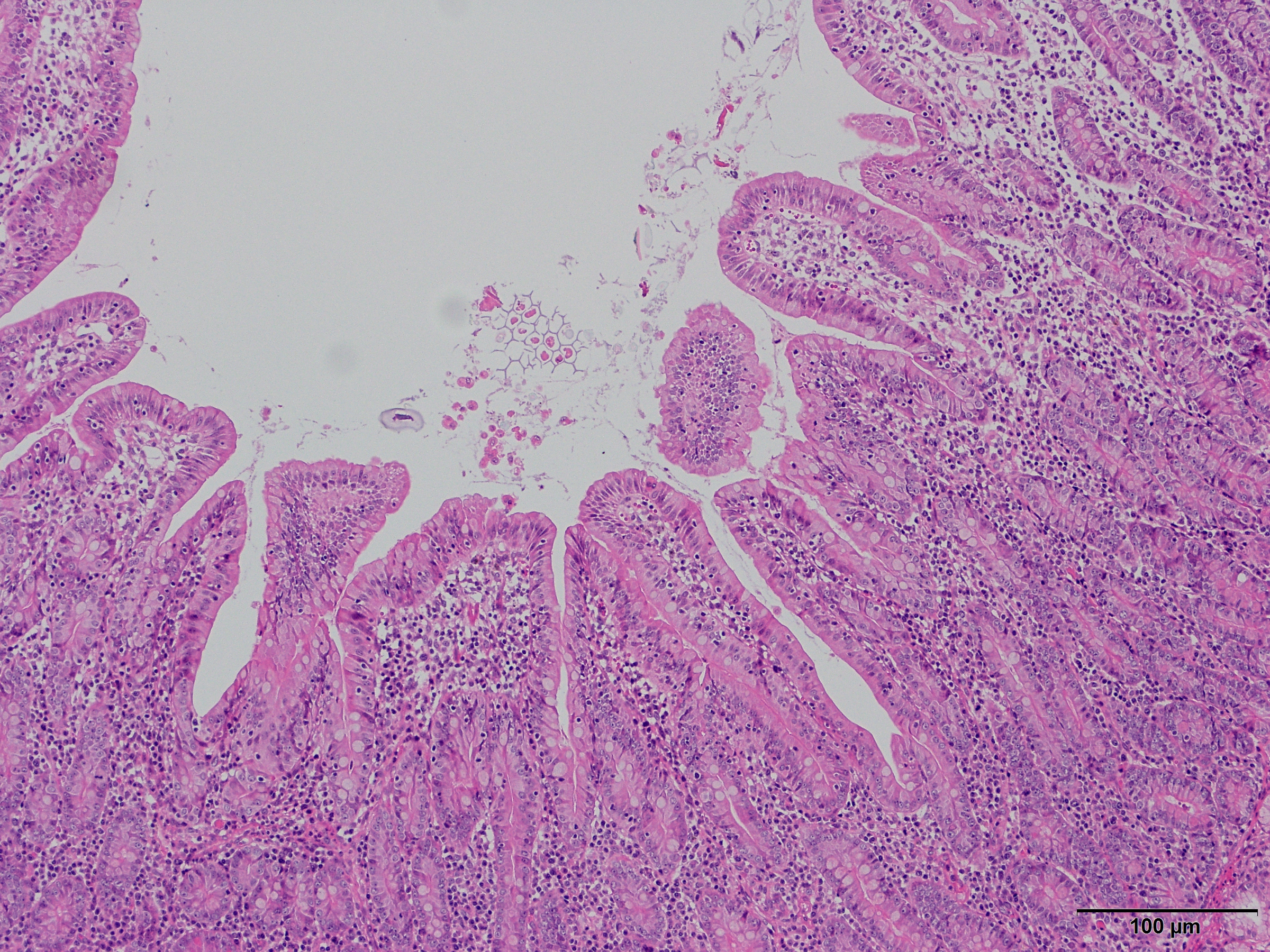

Supplement: Supplementary file 1 [file animals-16-01400-s001.zip › 1. Duodenum/180 mg kg CEO group/Duodenum-3-4.jpg]

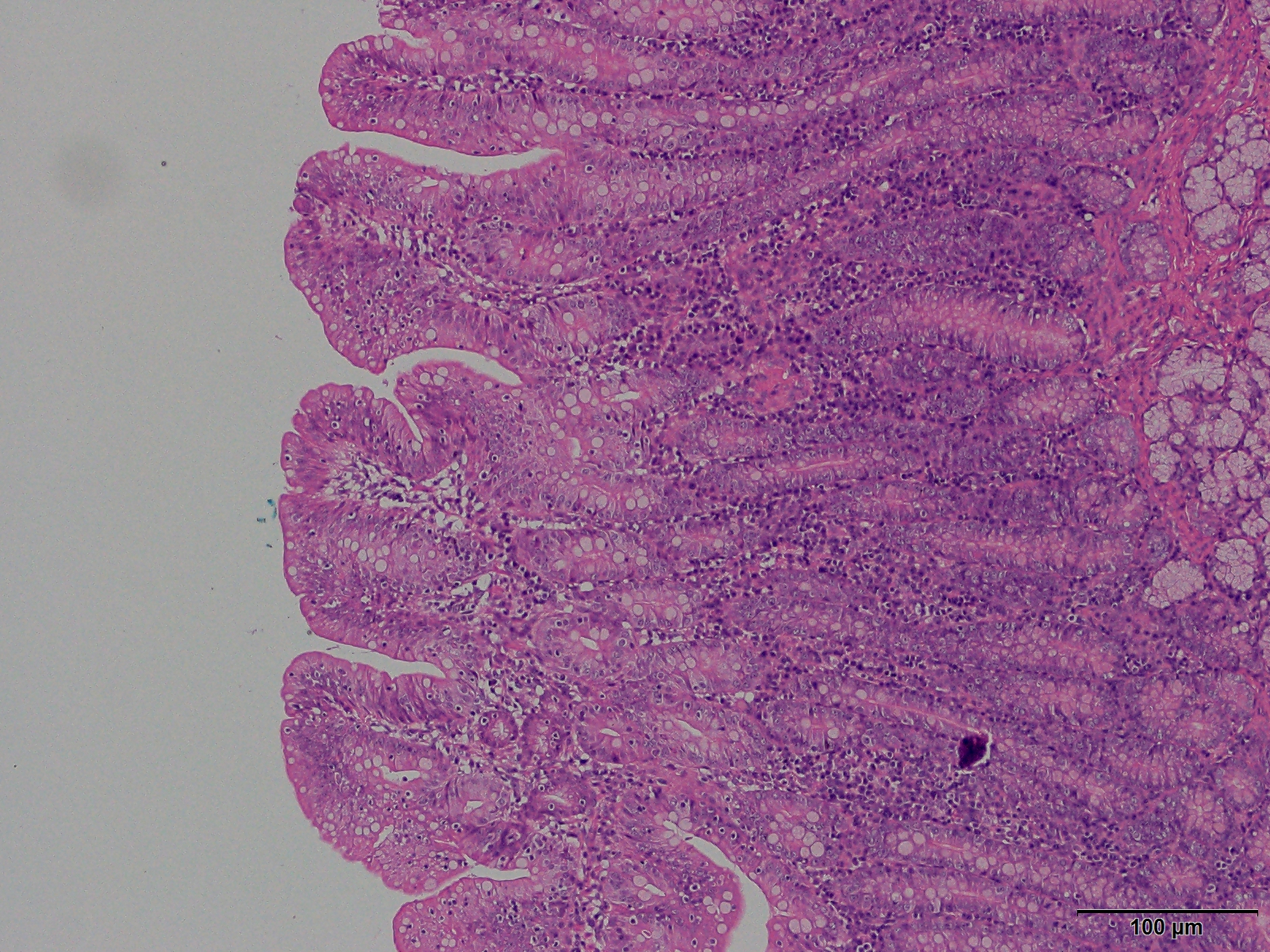

Supplement: Supplementary file 1 [file animals-16-01400-s001.zip › 1. Duodenum/180 mg kg CEO group/Duodenum-3-5.jpg]

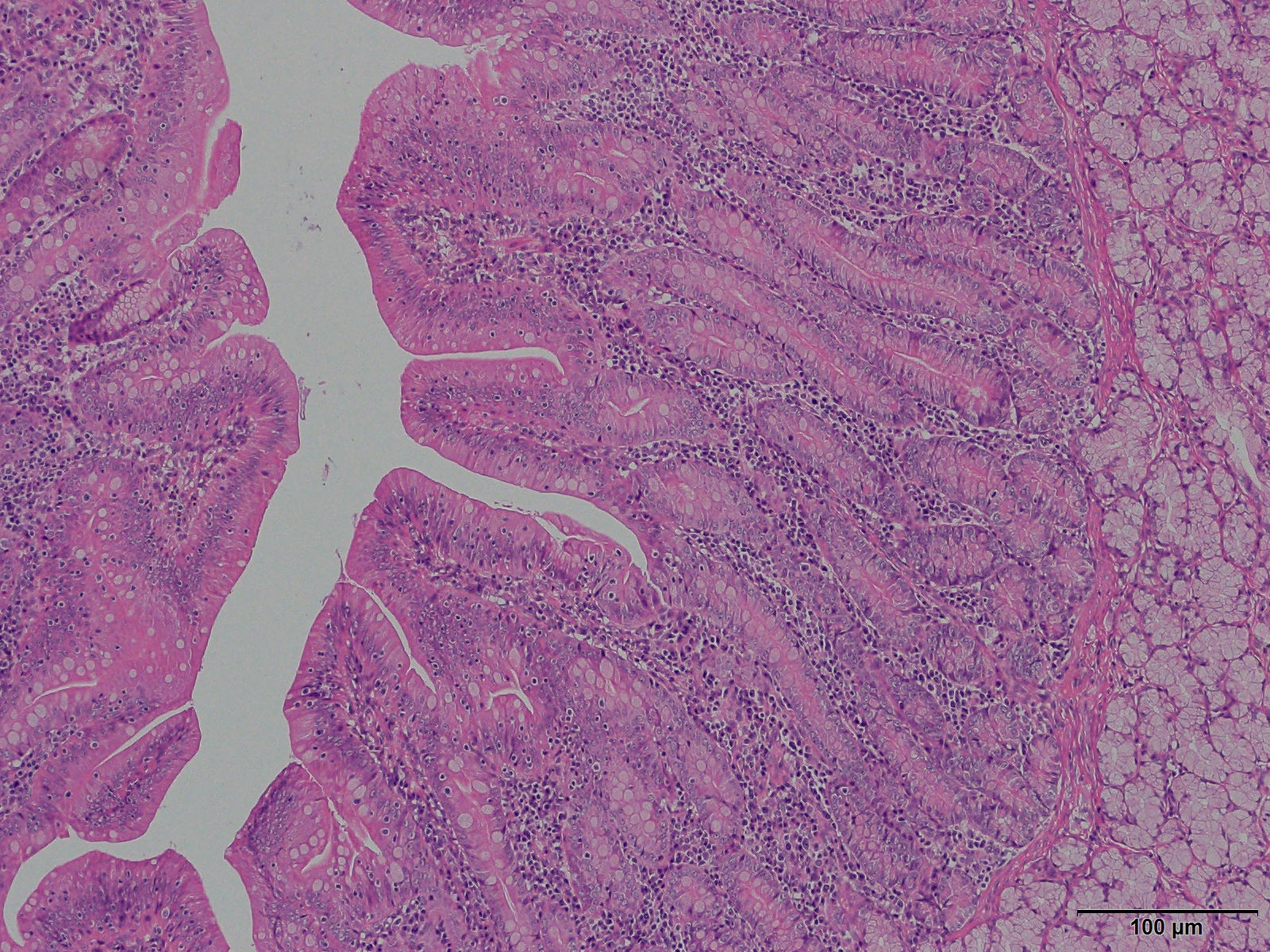

Supplement: Supplementary file 1 [file animals-16-01400-s001.zip › 1. Duodenum/180 mg kg CEO group/Duodenum-3-6.jpg]

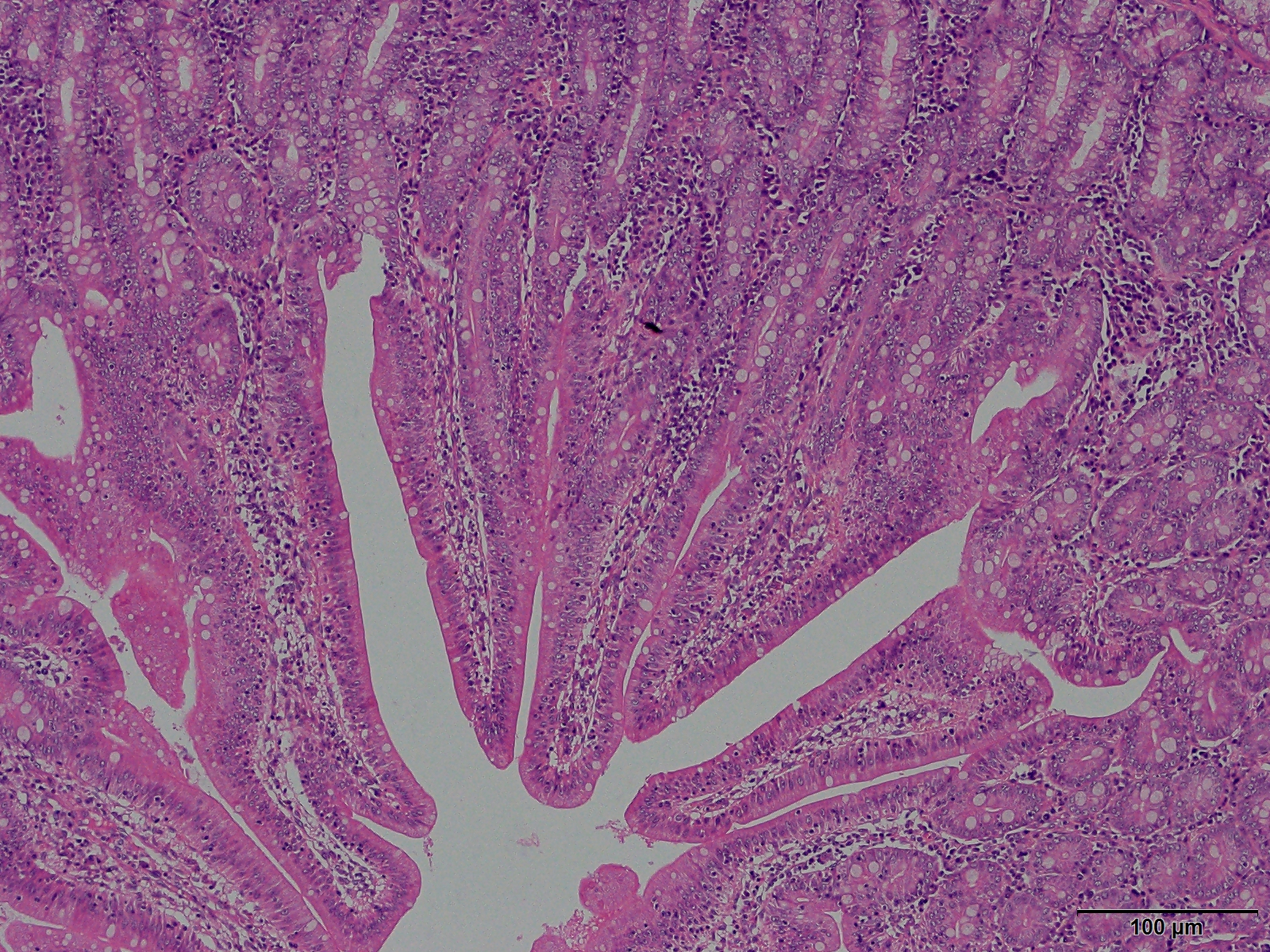

Supplement: Supplementary file 1 [file animals-16-01400-s001.zip › 1. Duodenum/180 mg kg CEO group/Duodenum-3-7.jpg]

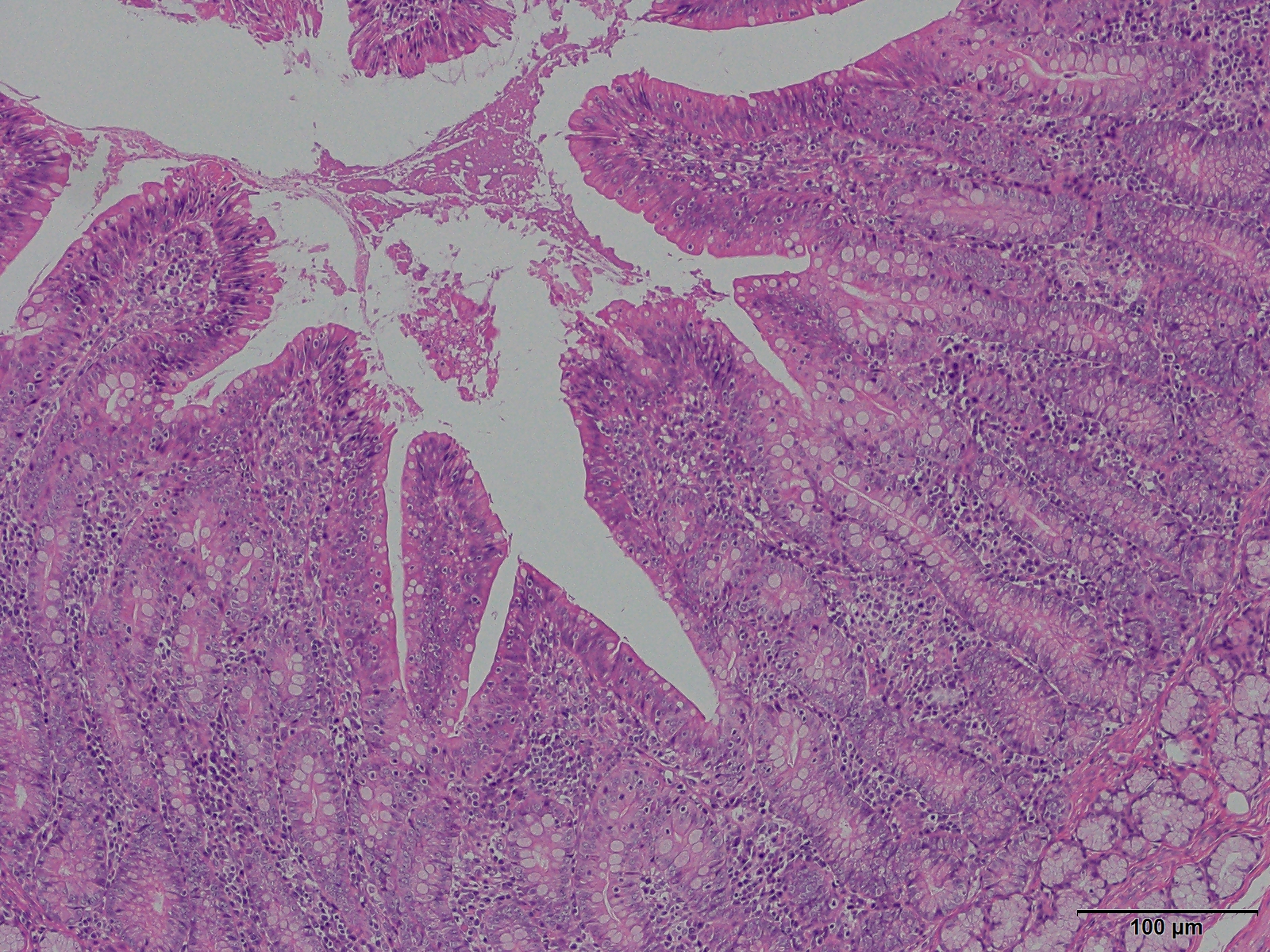

Supplement: Supplementary file 1 [file animals-16-01400-s001.zip › 1. Duodenum/180 mg kg CEO group/Duodenum-3-8.jpg]

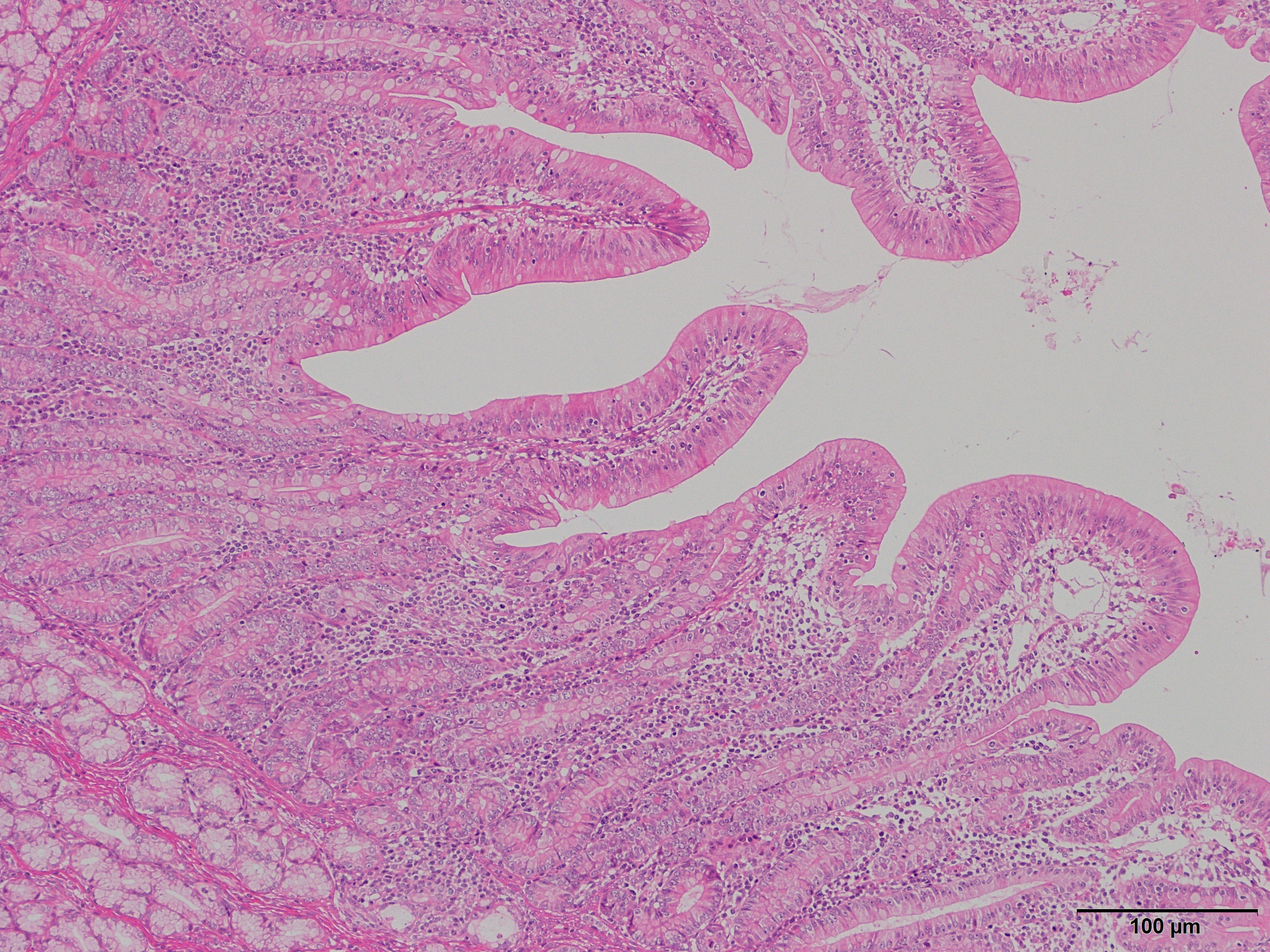

Supplement: Supplementary file 1 [file animals-16-01400-s001.zip › 1. Duodenum/240 mg kg CEO group/Duodenum-4-1.jpg]

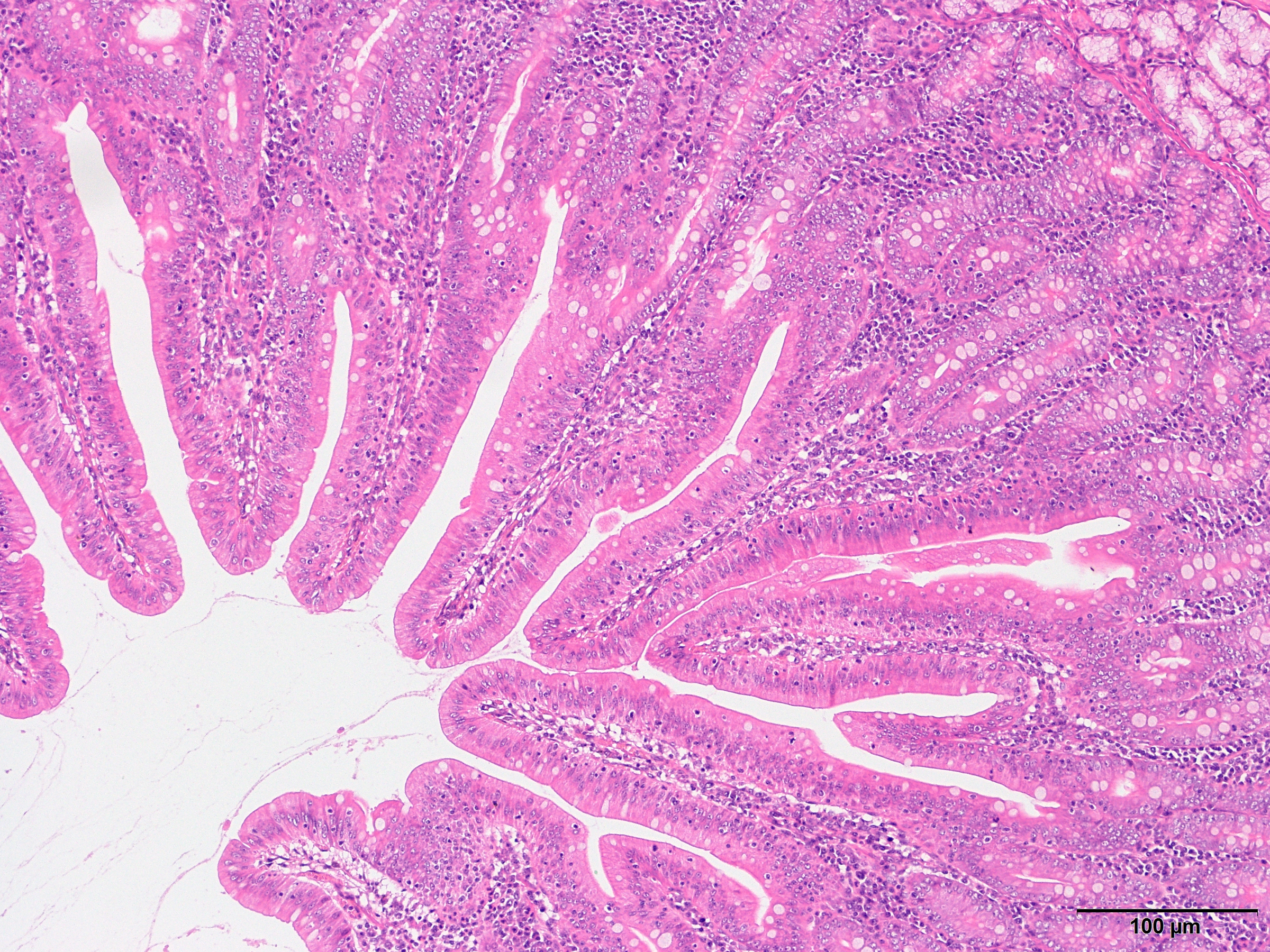

Supplement: Supplementary file 1 [file animals-16-01400-s001.zip › 1. Duodenum/240 mg kg CEO group/Duodenum-4-2-Figure 3A.jpg]

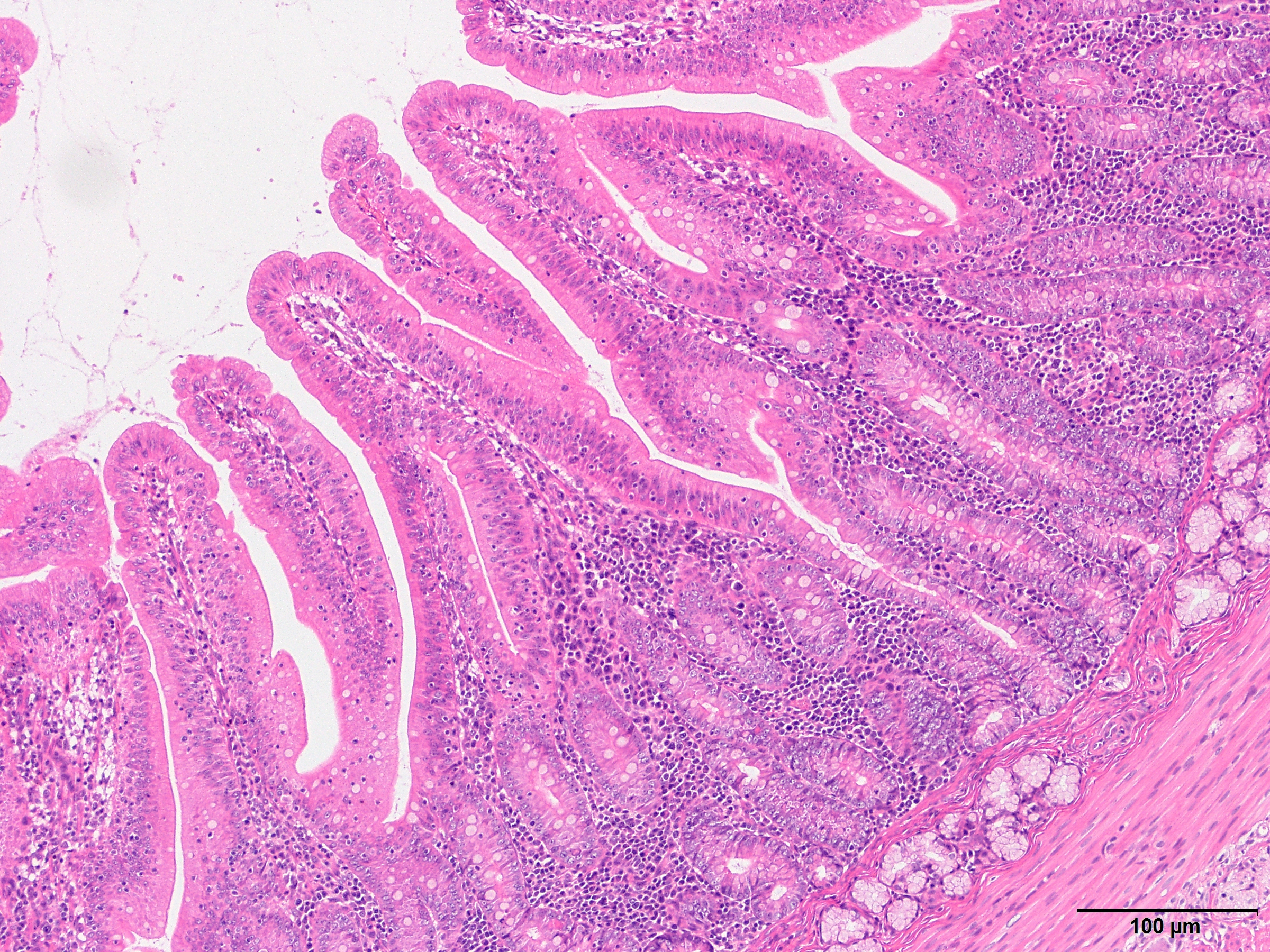

Supplement: Supplementary file 1 [file animals-16-01400-s001.zip › 1. Duodenum/240 mg kg CEO group/Duodenum-4-3.jpg]

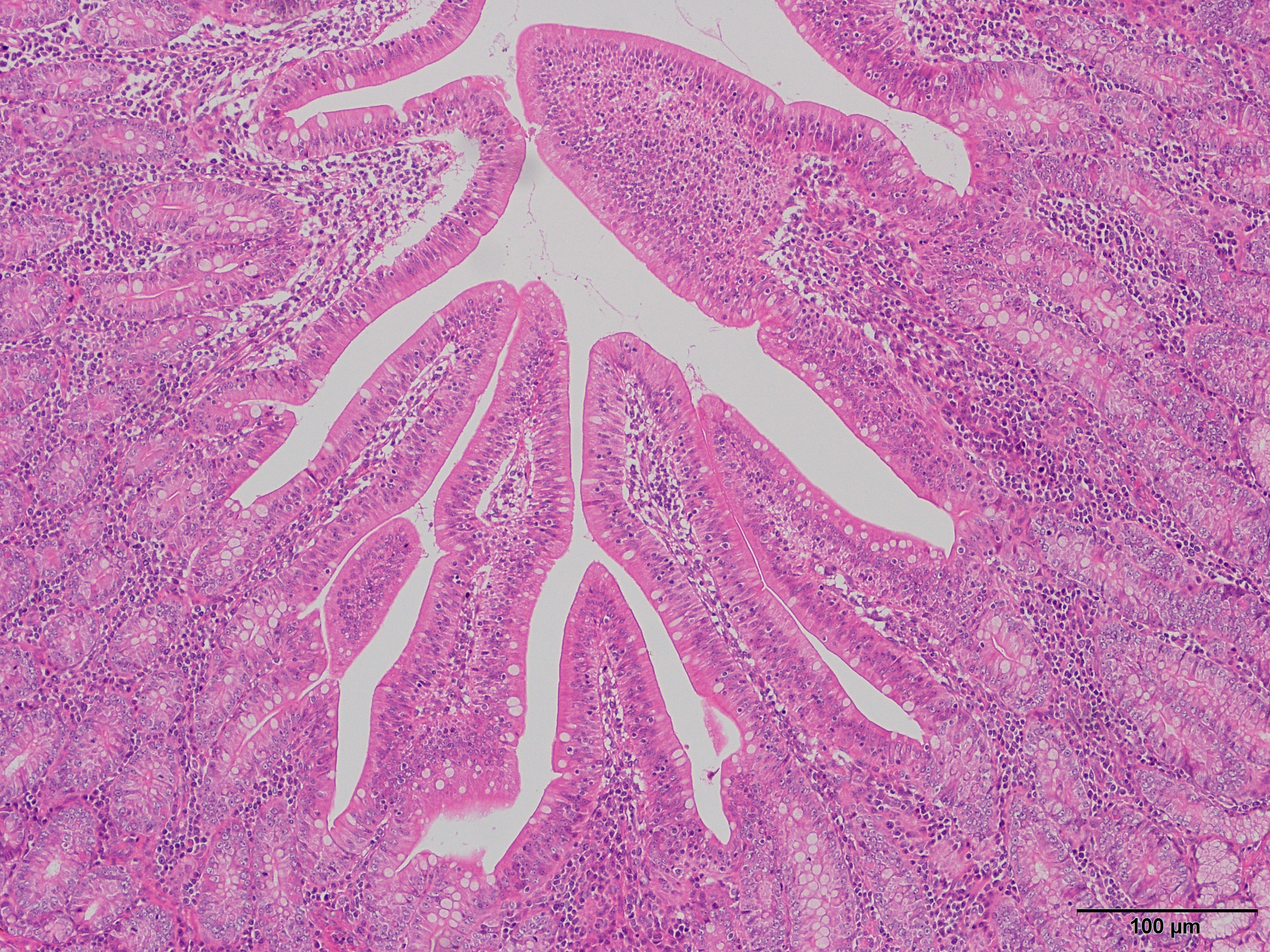

Supplement: Supplementary file 1 [file animals-16-01400-s001.zip › 1. Duodenum/240 mg kg CEO group/Duodenum-4-4.jpg]

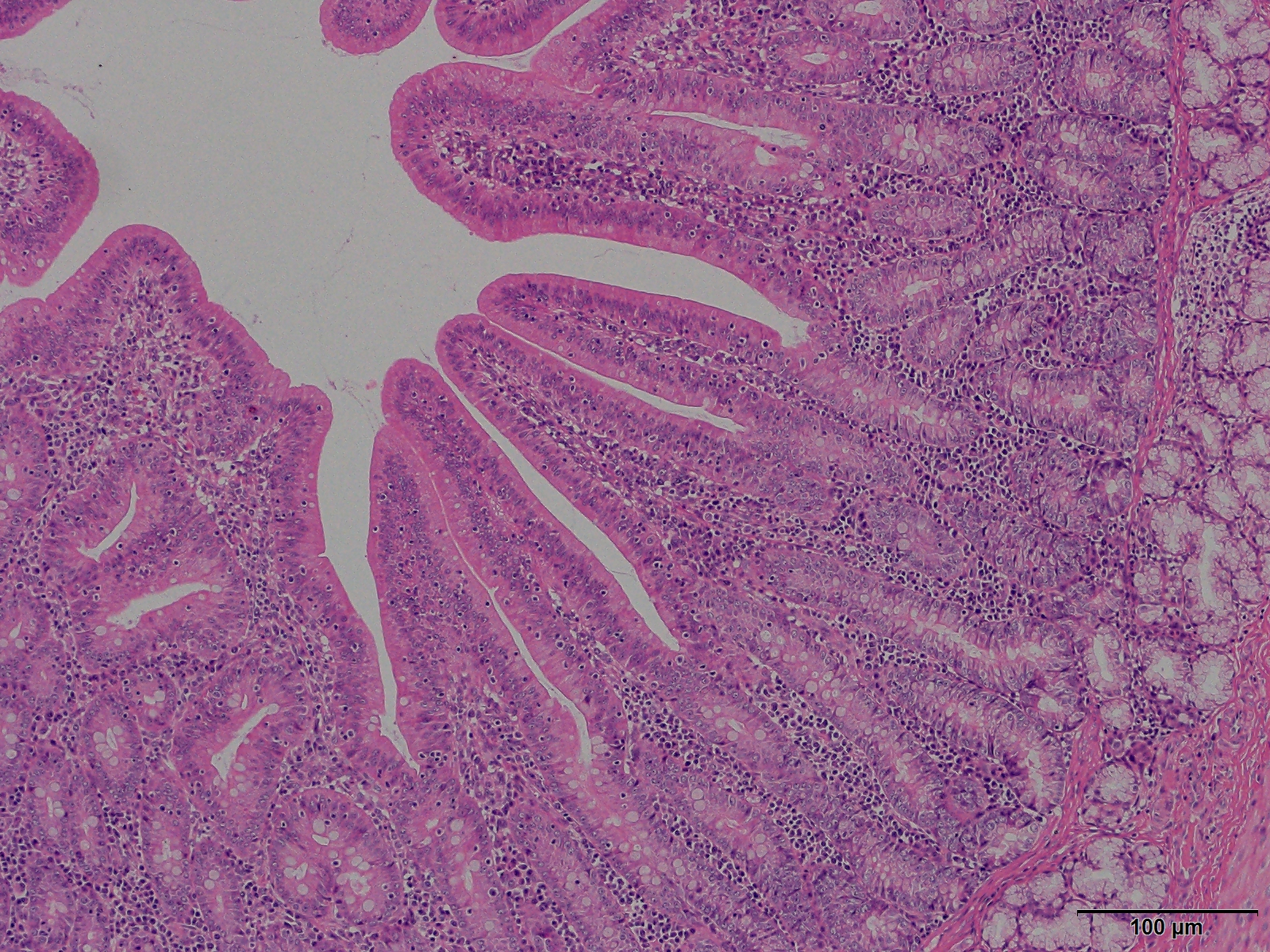

Supplement: Supplementary file 1 [file animals-16-01400-s001.zip › 1. Duodenum/240 mg kg CEO group/Duodenum-4-5.jpg]

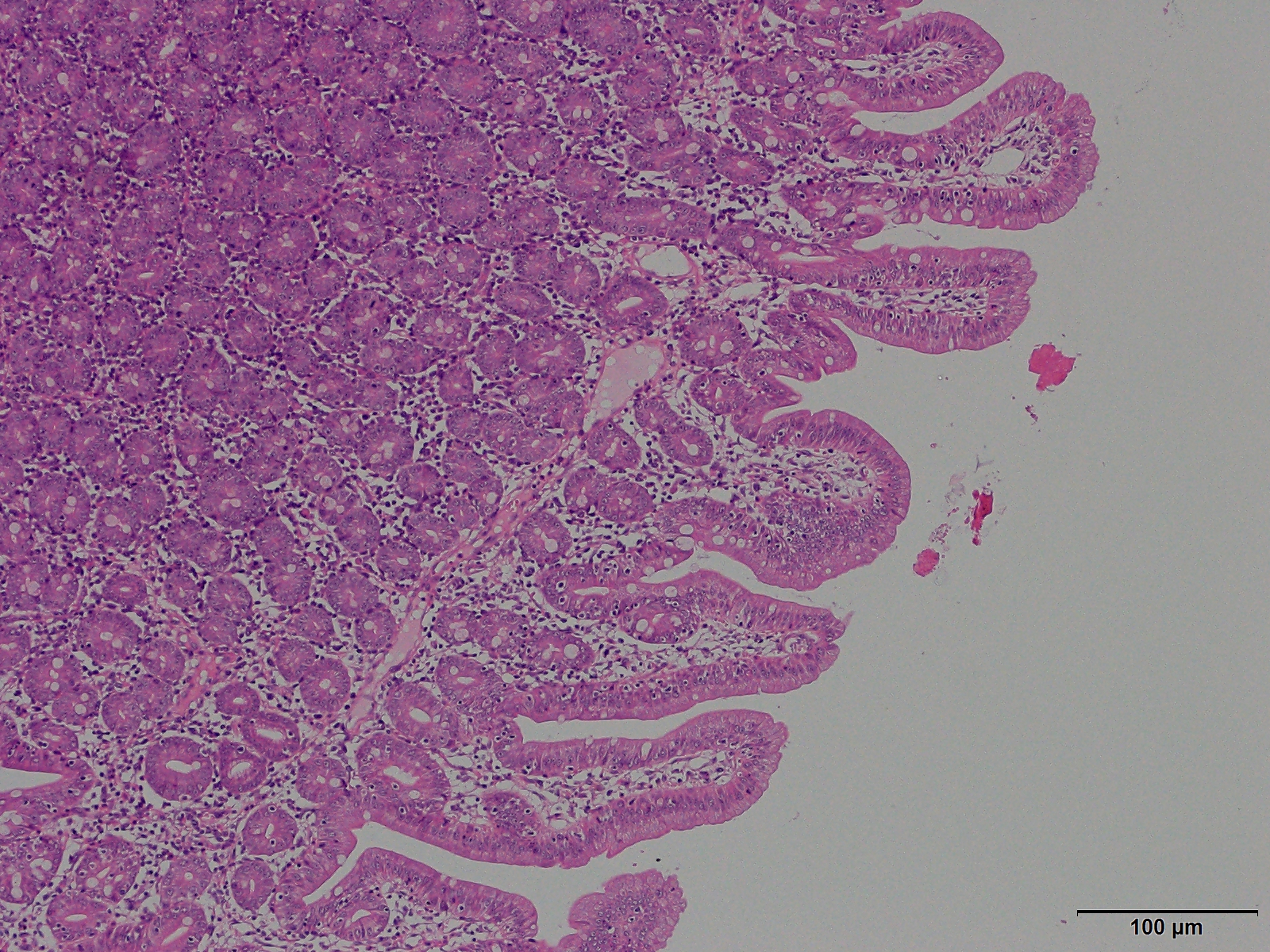

Supplement: Supplementary file 1 [file animals-16-01400-s001.zip › 1. Duodenum/240 mg kg CEO group/Duodenum-4-6.jpg]

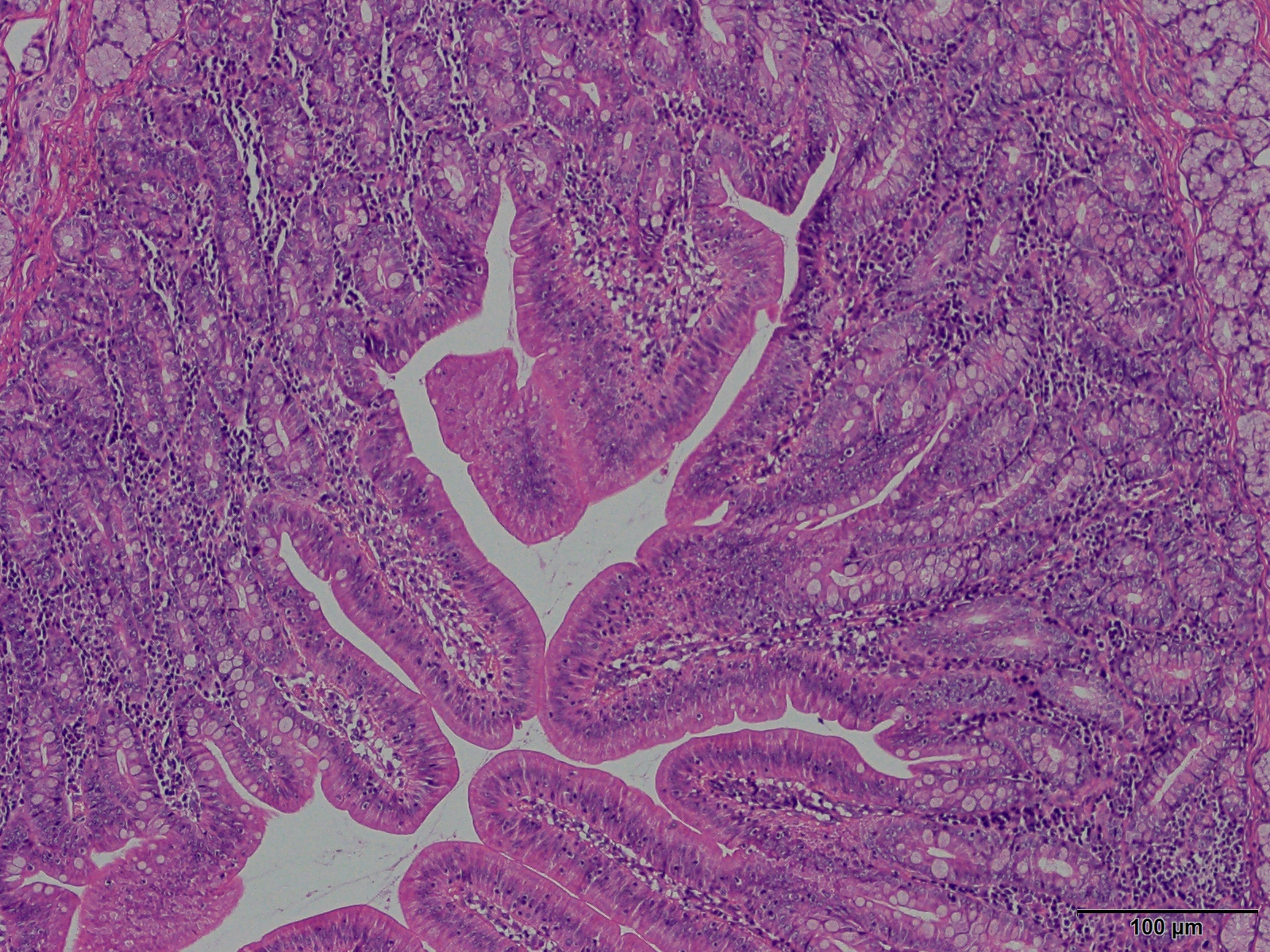

Supplement: Supplementary file 1 [file animals-16-01400-s001.zip › 1. Duodenum/240 mg kg CEO group/Duodenum-4-7.jpg]

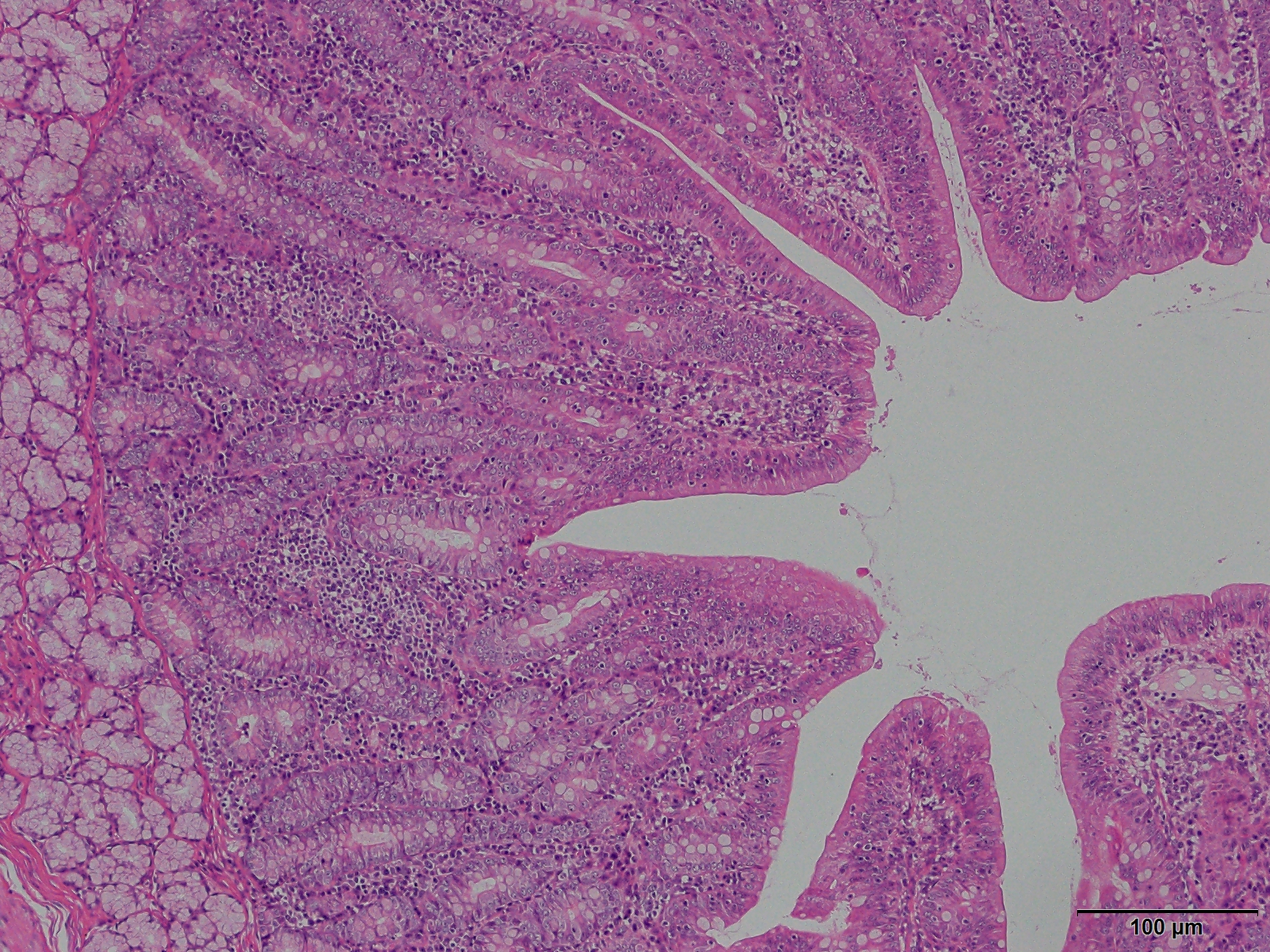

Supplement: Supplementary file 1 [file animals-16-01400-s001.zip › 1. Duodenum/240 mg kg CEO group/Duodenum-4-8.jpg]

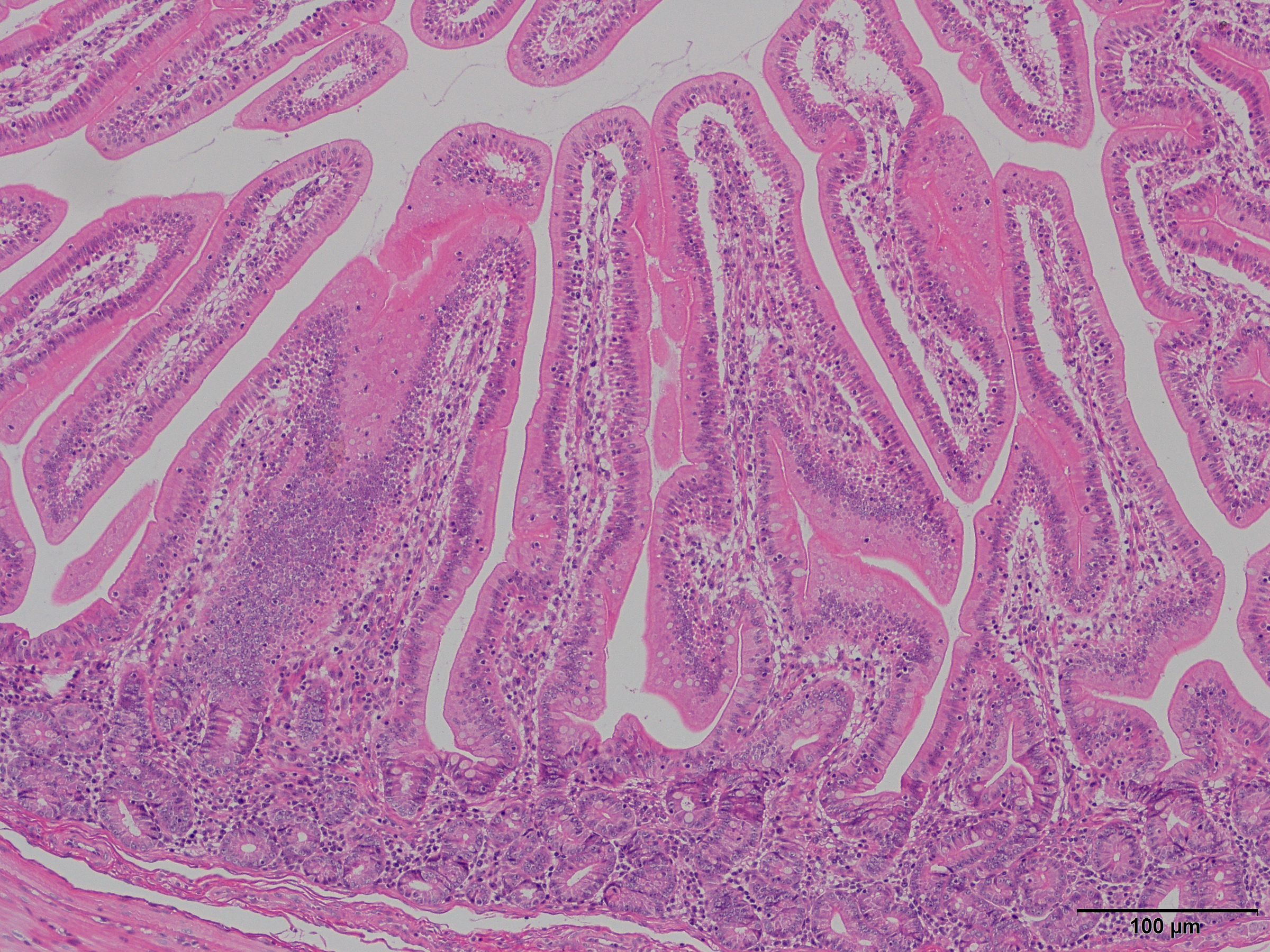

Supplement: Supplementary file 1 [file animals-16-01400-s001.zip › 2. Jejunum/0 CEO group/Jejunum-1-1.jpg]

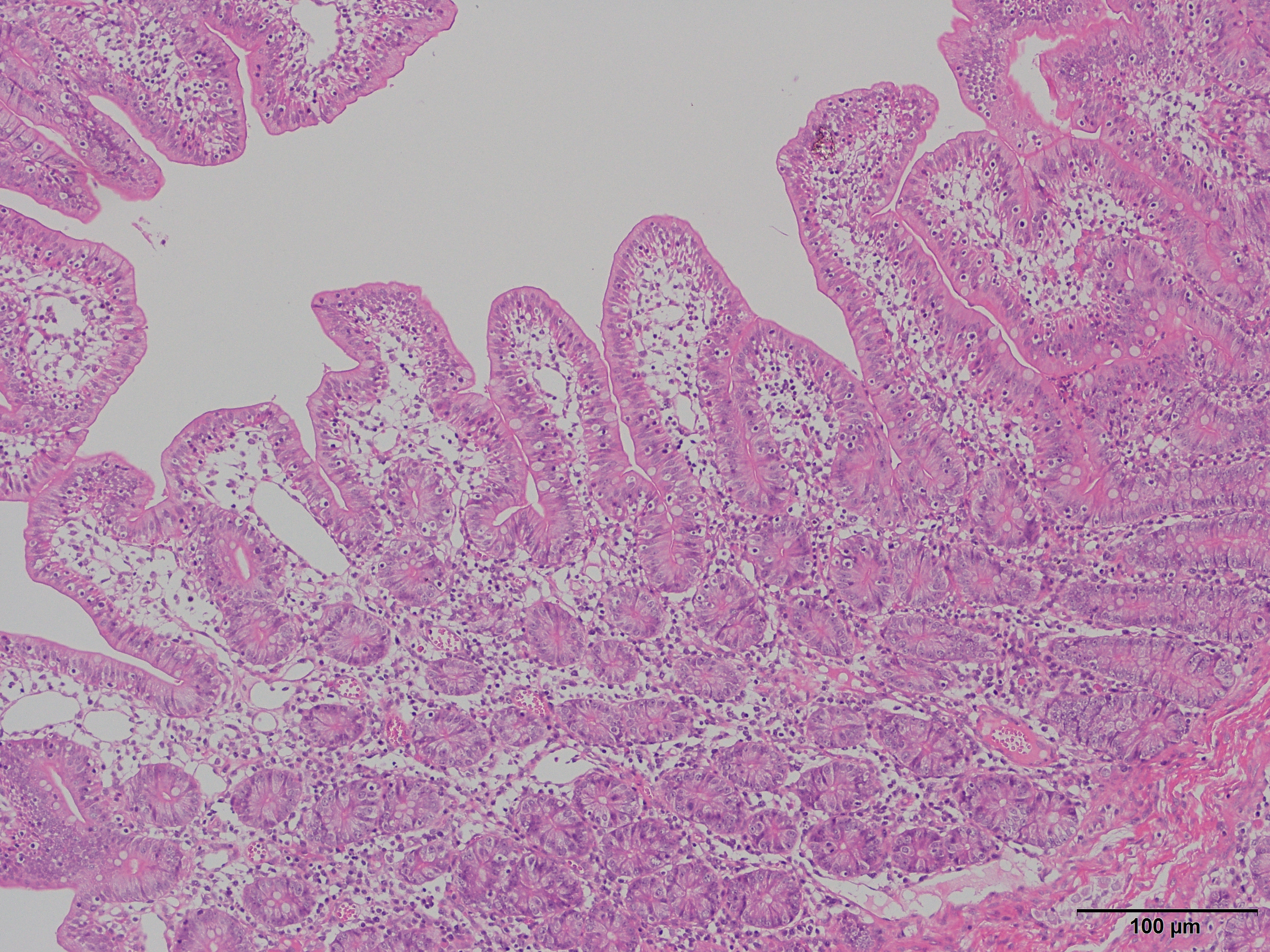

Supplement: Supplementary file 1 [file animals-16-01400-s001.zip › 2. Jejunum/0 CEO group/Jejunum-1-2.jpg]

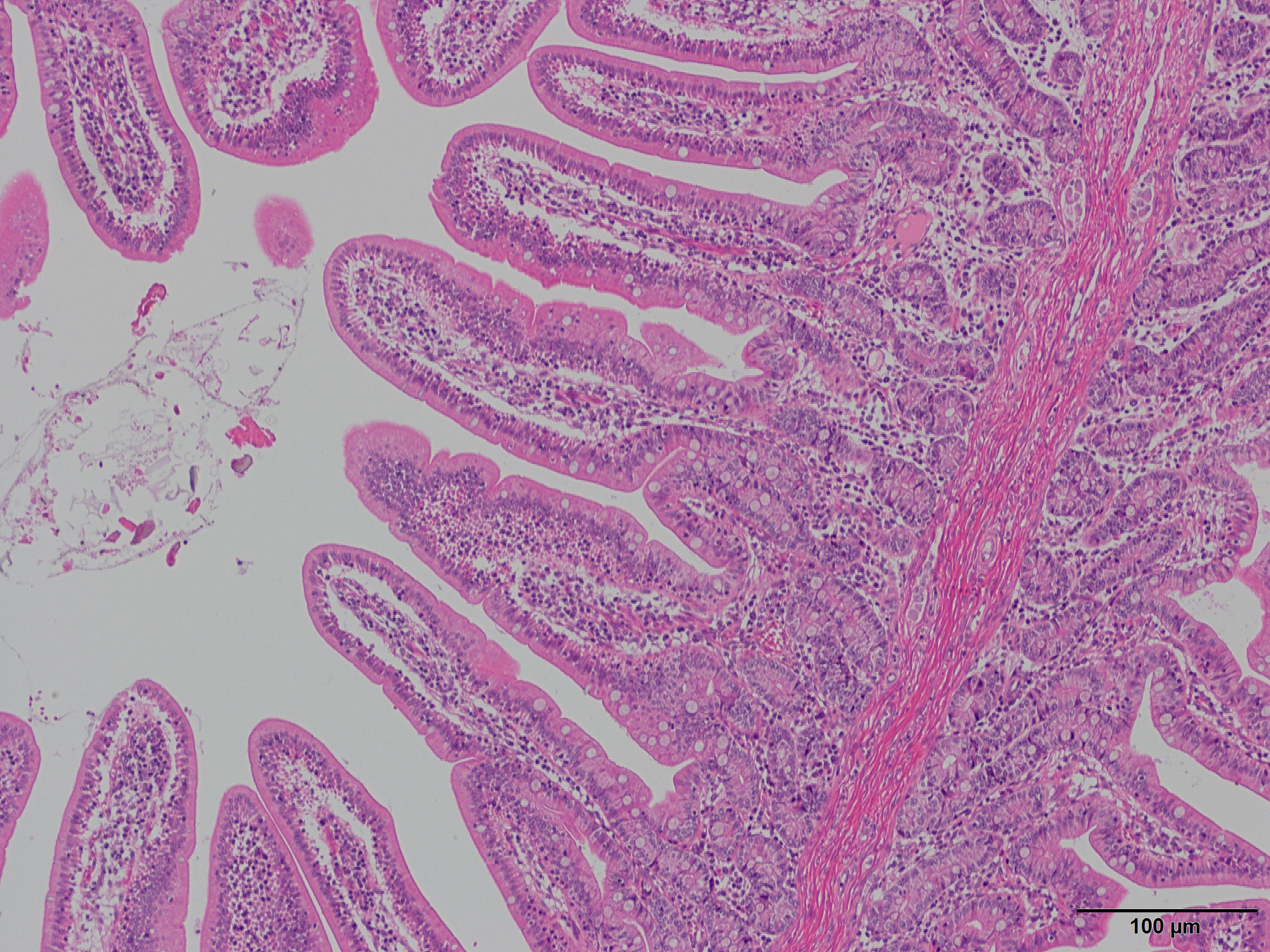

Supplement: Supplementary file 1 [file animals-16-01400-s001.zip › 2. Jejunum/0 CEO group/Jejunum-1-3.jpg]

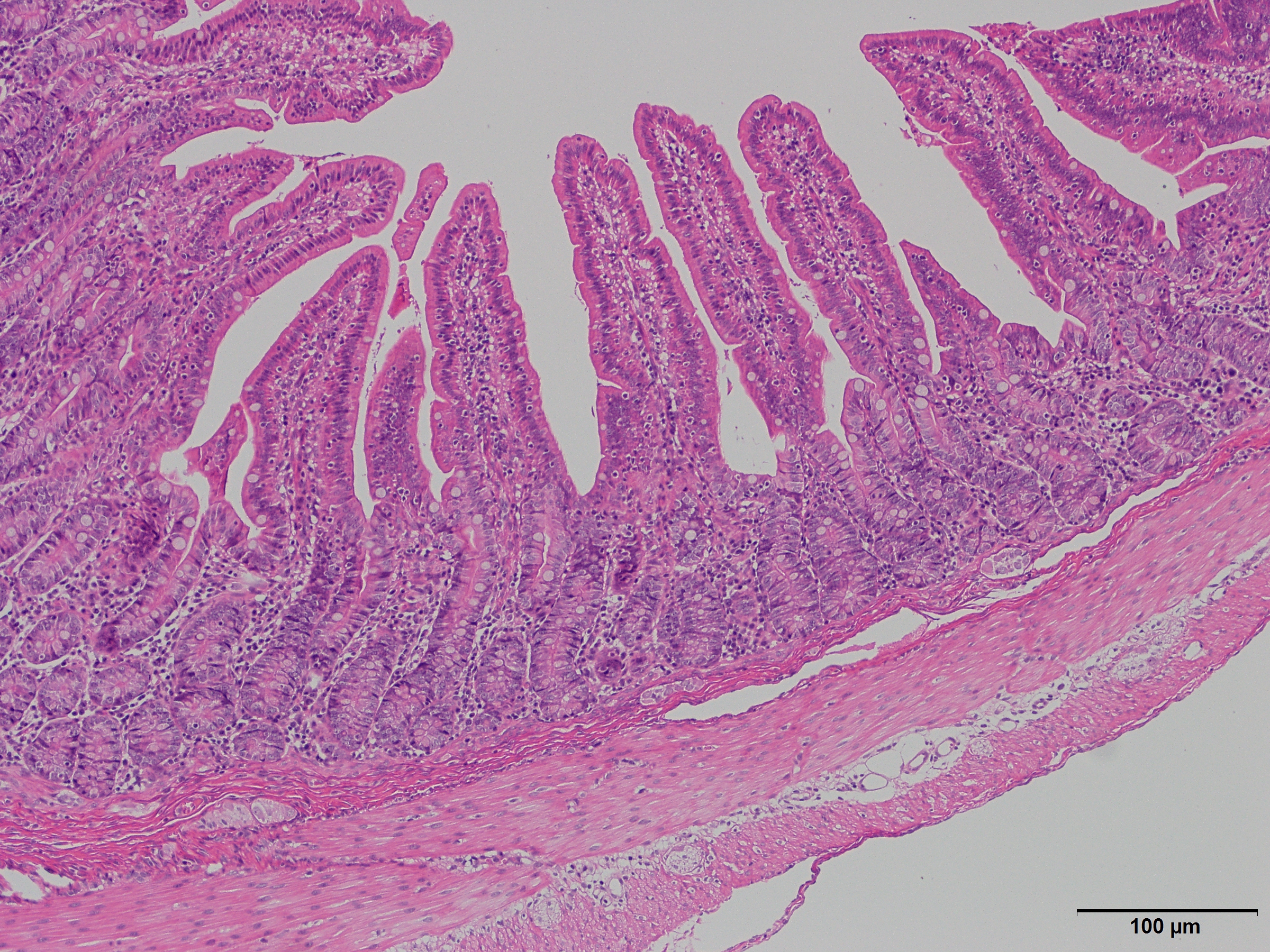

Supplement: Supplementary file 1 [file animals-16-01400-s001.zip › 2. Jejunum/0 CEO group/Jejunum-1-4-Figure 3A.jpg]

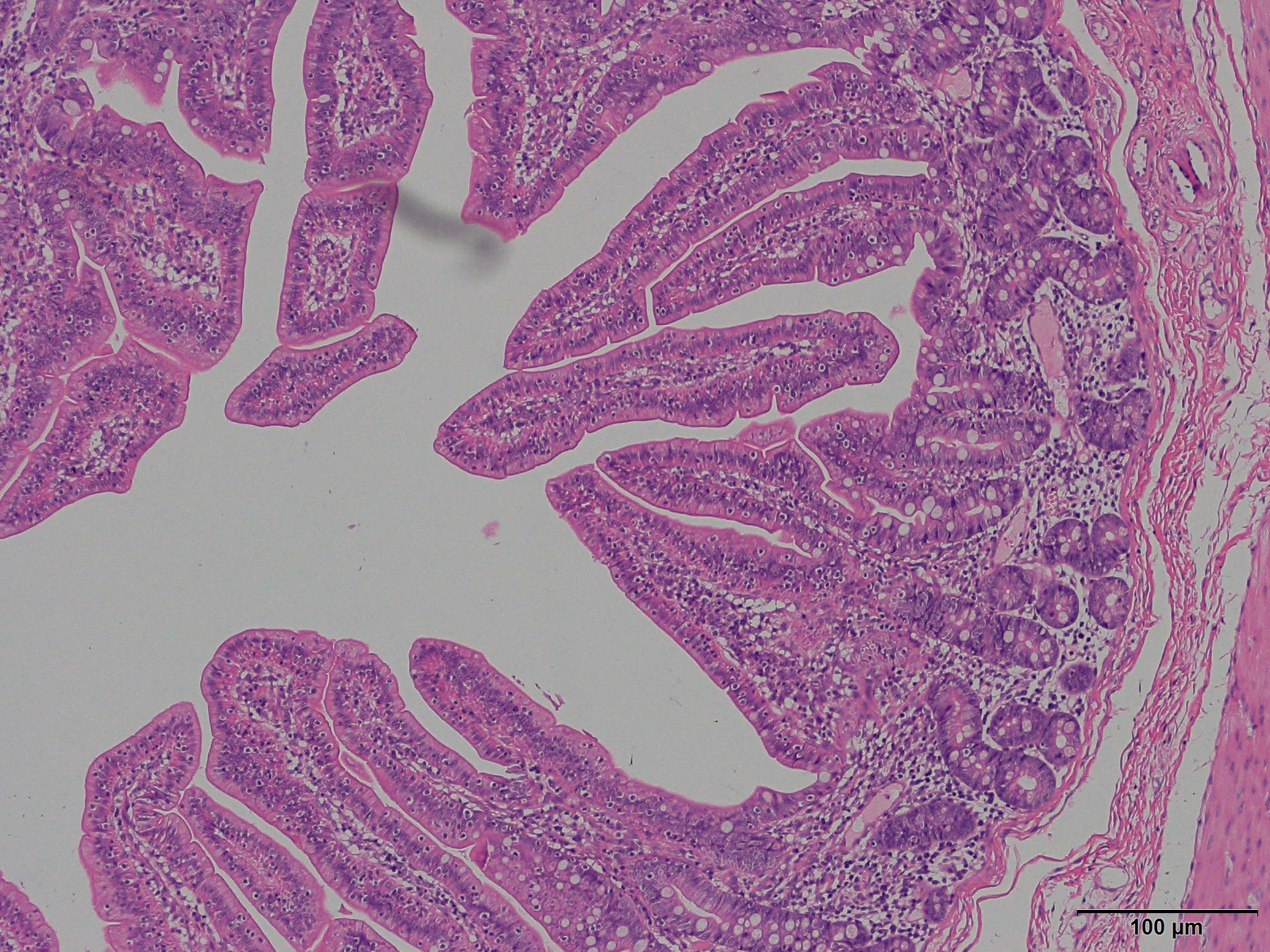

Supplement: Supplementary file 1 [file animals-16-01400-s001.zip › 2. Jejunum/0 CEO group/Jejunum-1-5.jpg]

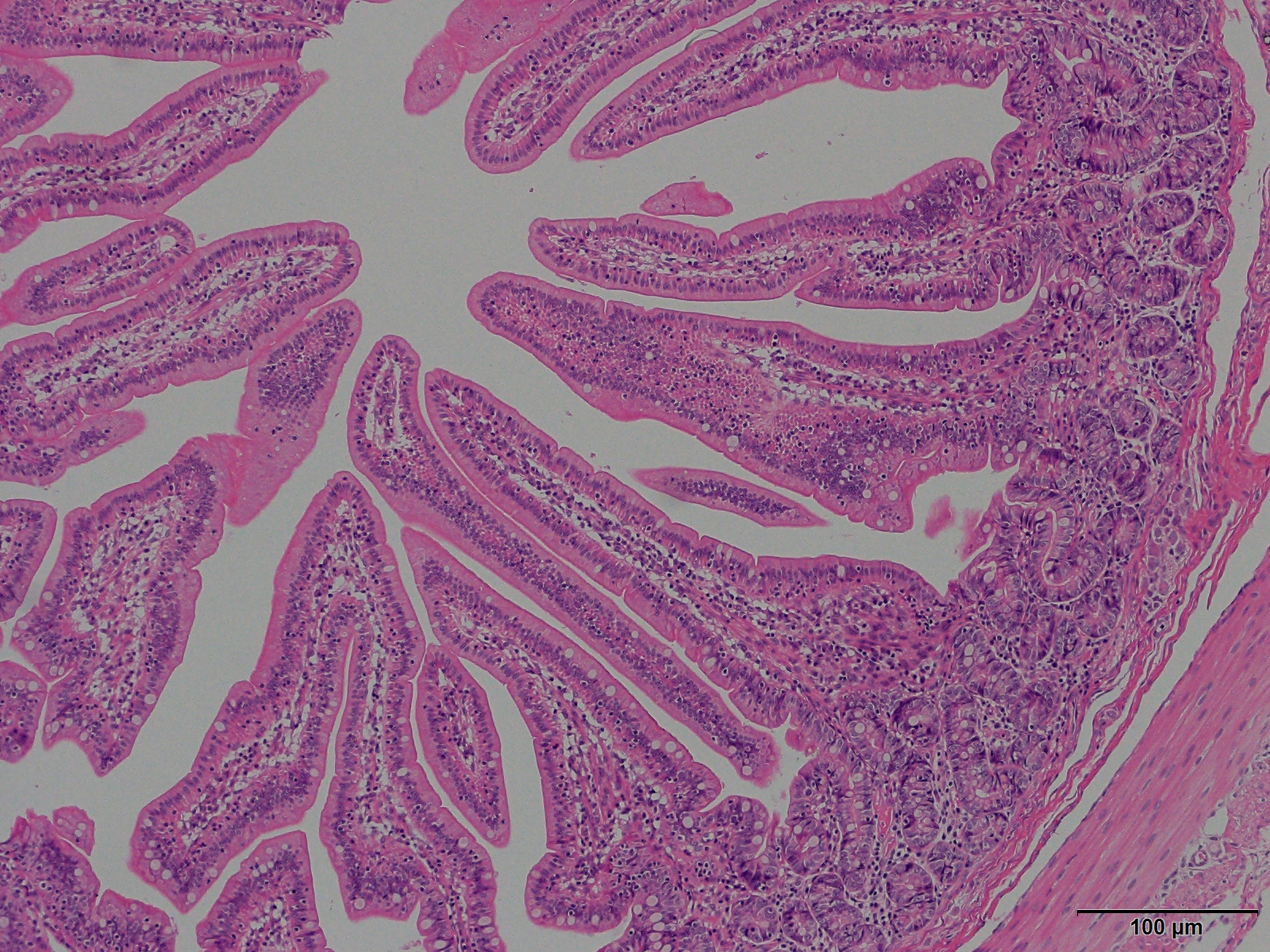

Supplement: Supplementary file 1 [file animals-16-01400-s001.zip › 2. Jejunum/0 CEO group/Jejunum-1-6.jpg]

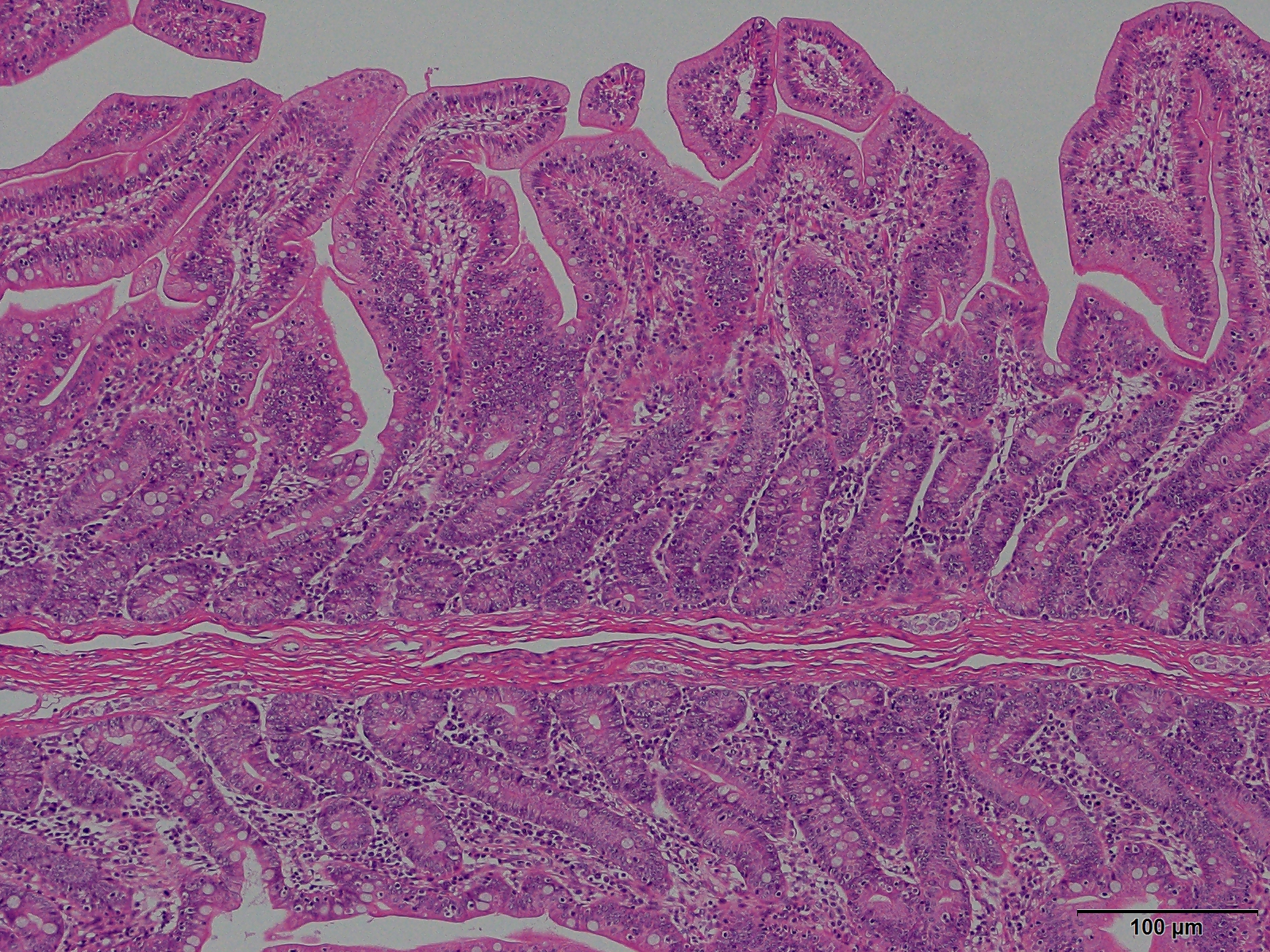

Supplement: Supplementary file 1 [file animals-16-01400-s001.zip › 2. Jejunum/0 CEO group/Jejunum-1-7.jpg]

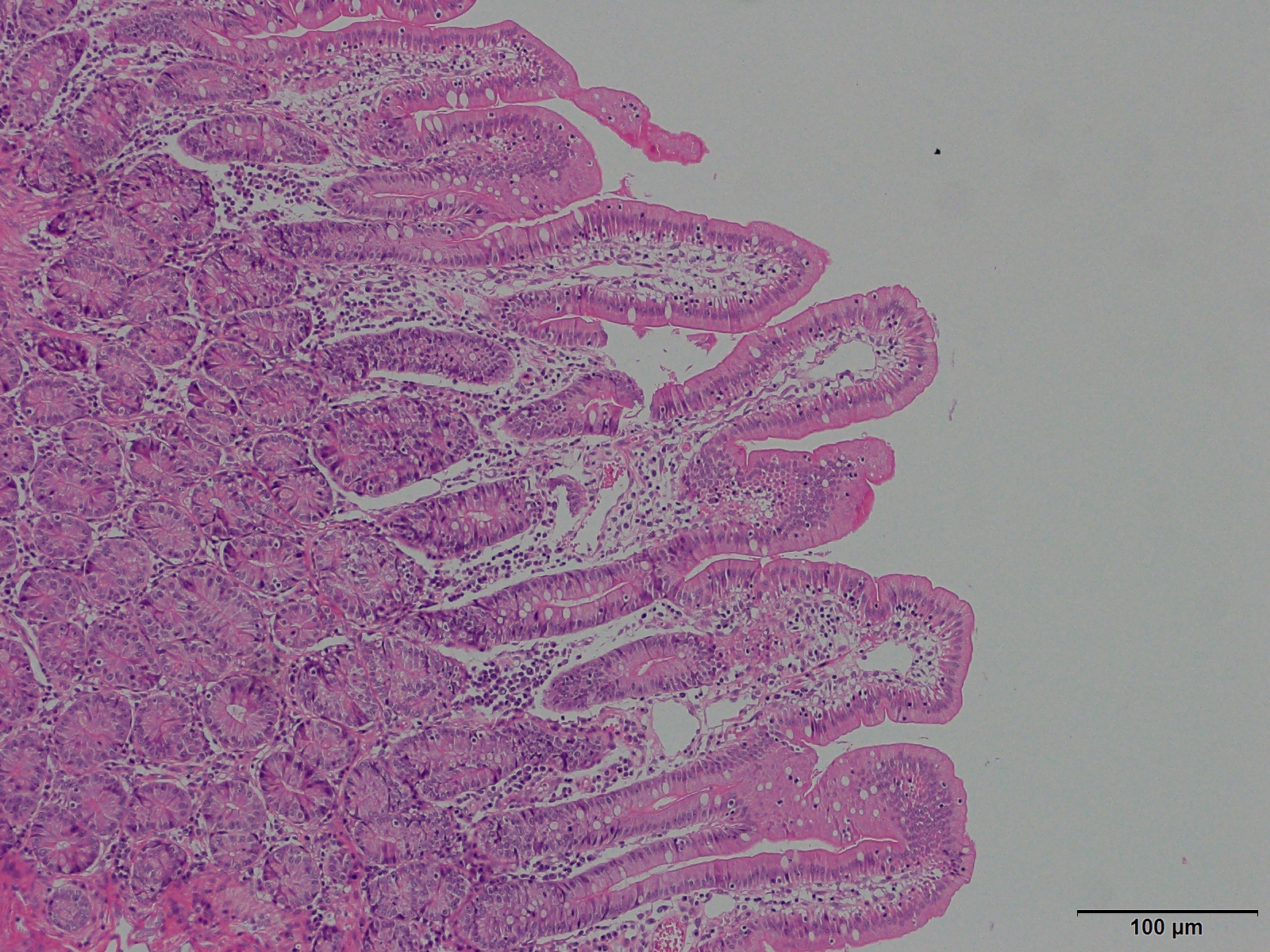

Supplement: Supplementary file 1 [file animals-16-01400-s001.zip › 2. Jejunum/0 CEO group/Jejunum-1-8.jpg]

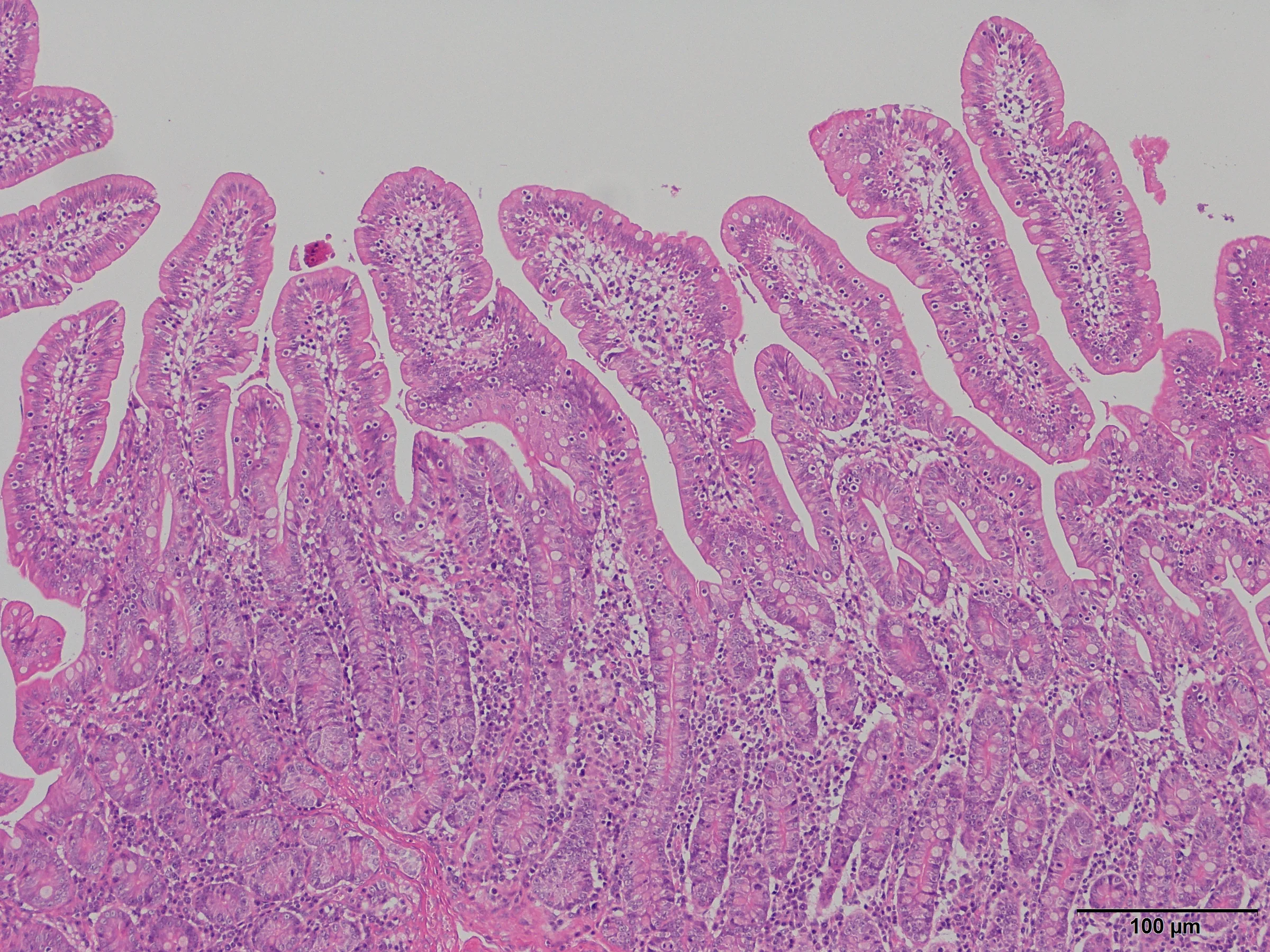

Supplement: Supplementary file 1 [file animals-16-01400-s001.zip › 2. Jejunum/120 mg kg CEO group/Jejunum-2-1.webp]

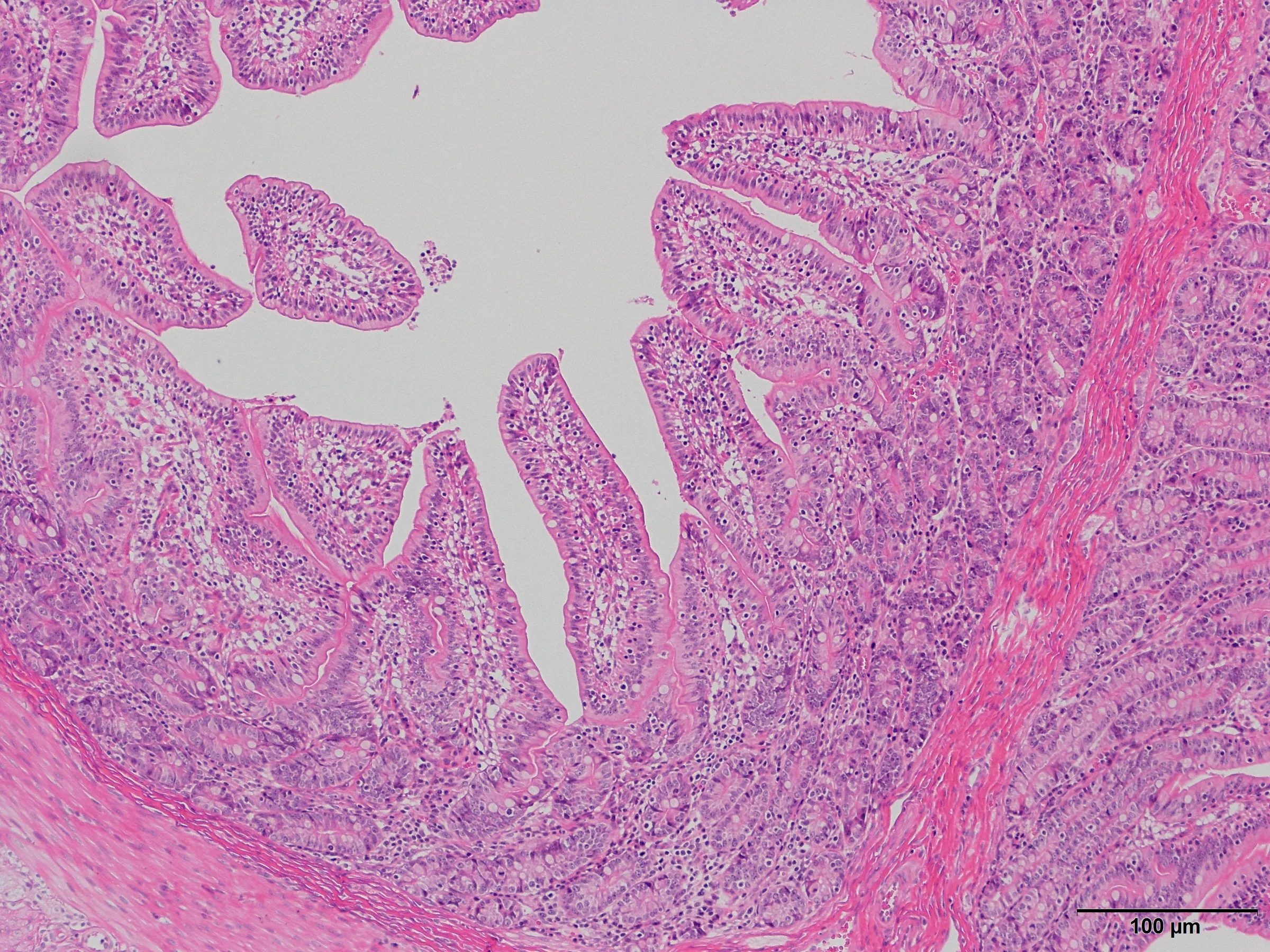

Supplement: Supplementary file 1 [file animals-16-01400-s001.zip › 2. Jejunum/120 mg kg CEO group/Jejunum-2-2.webp]

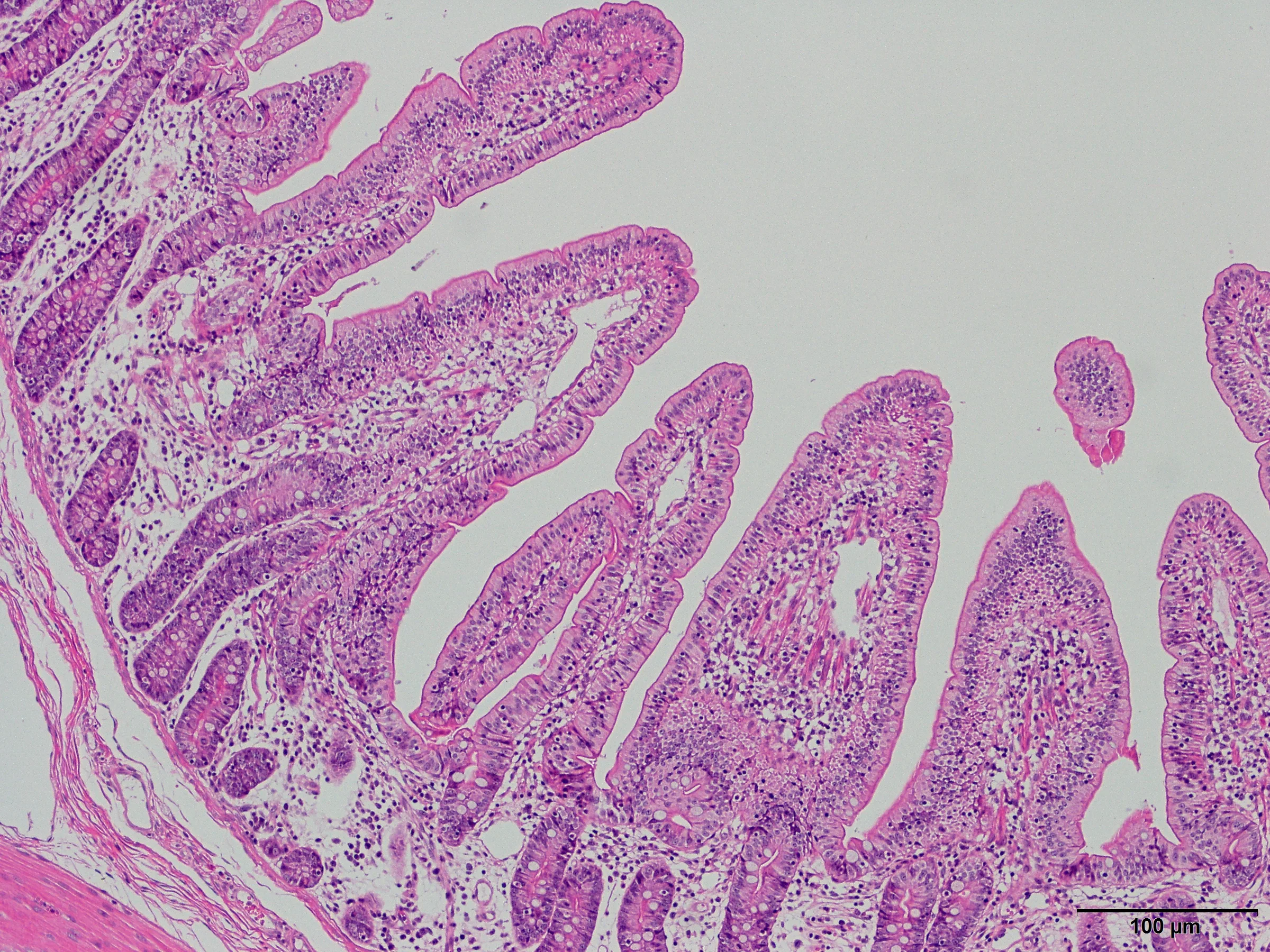

Supplement: Supplementary file 1 [file animals-16-01400-s001.zip › 2. Jejunum/120 mg kg CEO group/Jejunum-2-3.webp]

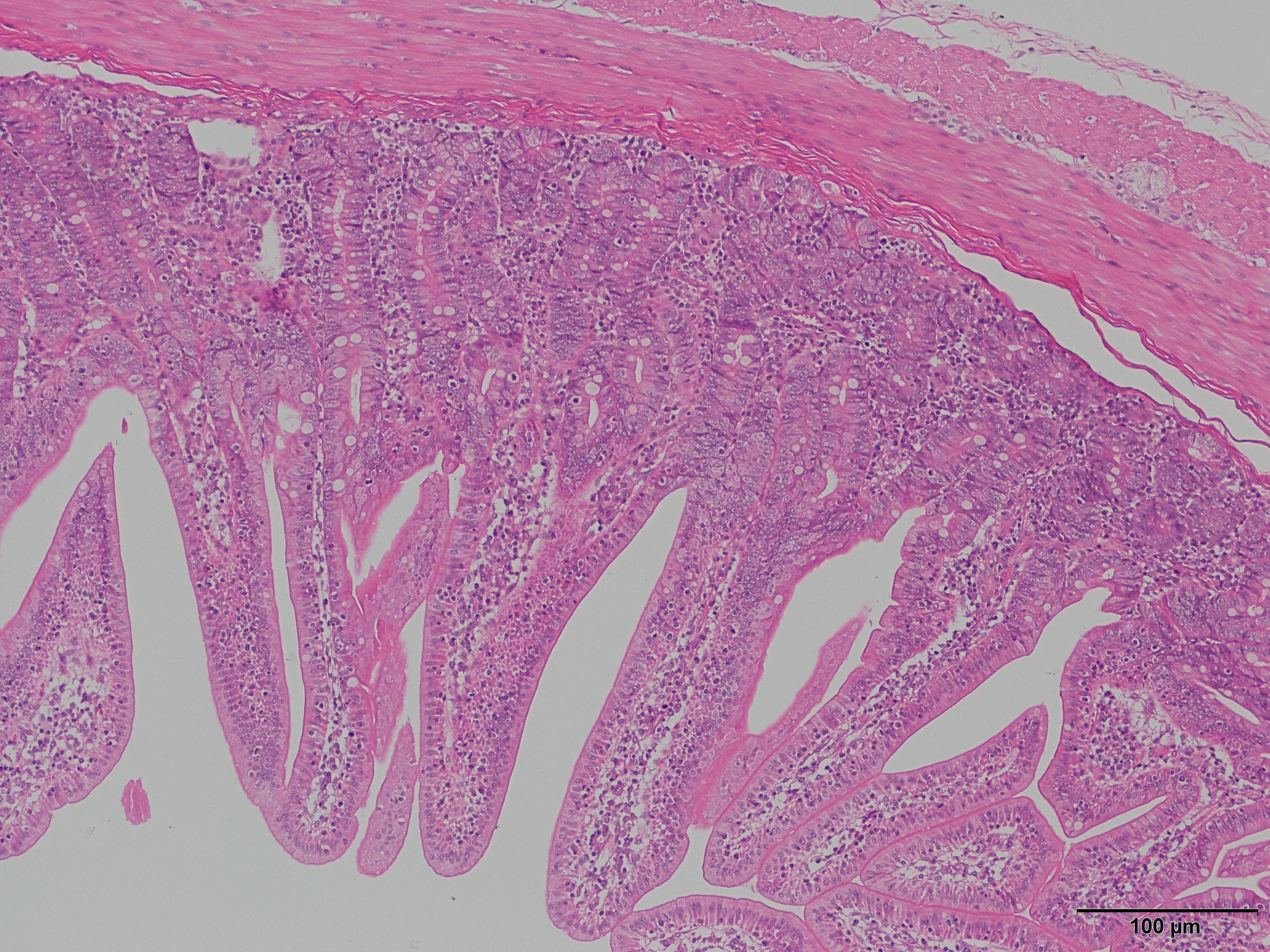

Supplement: Supplementary file 1 [file animals-16-01400-s001.zip › 2. Jejunum/120 mg kg CEO group/Jejunum-2-4.webp]

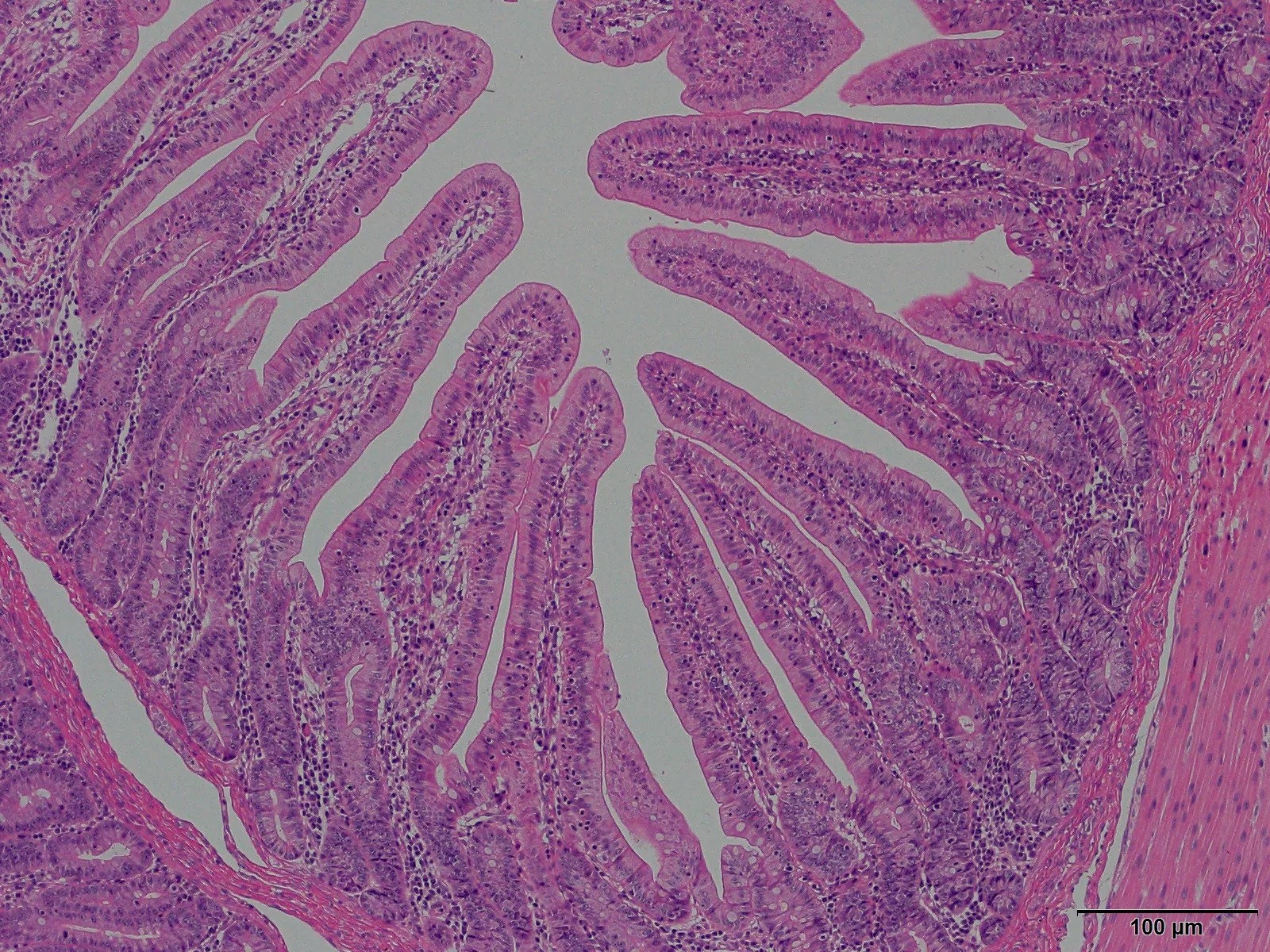

Supplement: Supplementary file 1 [file animals-16-01400-s001.zip › 2. Jejunum/120 mg kg CEO group/Jejunum-2-5-Figure 3A.webp]

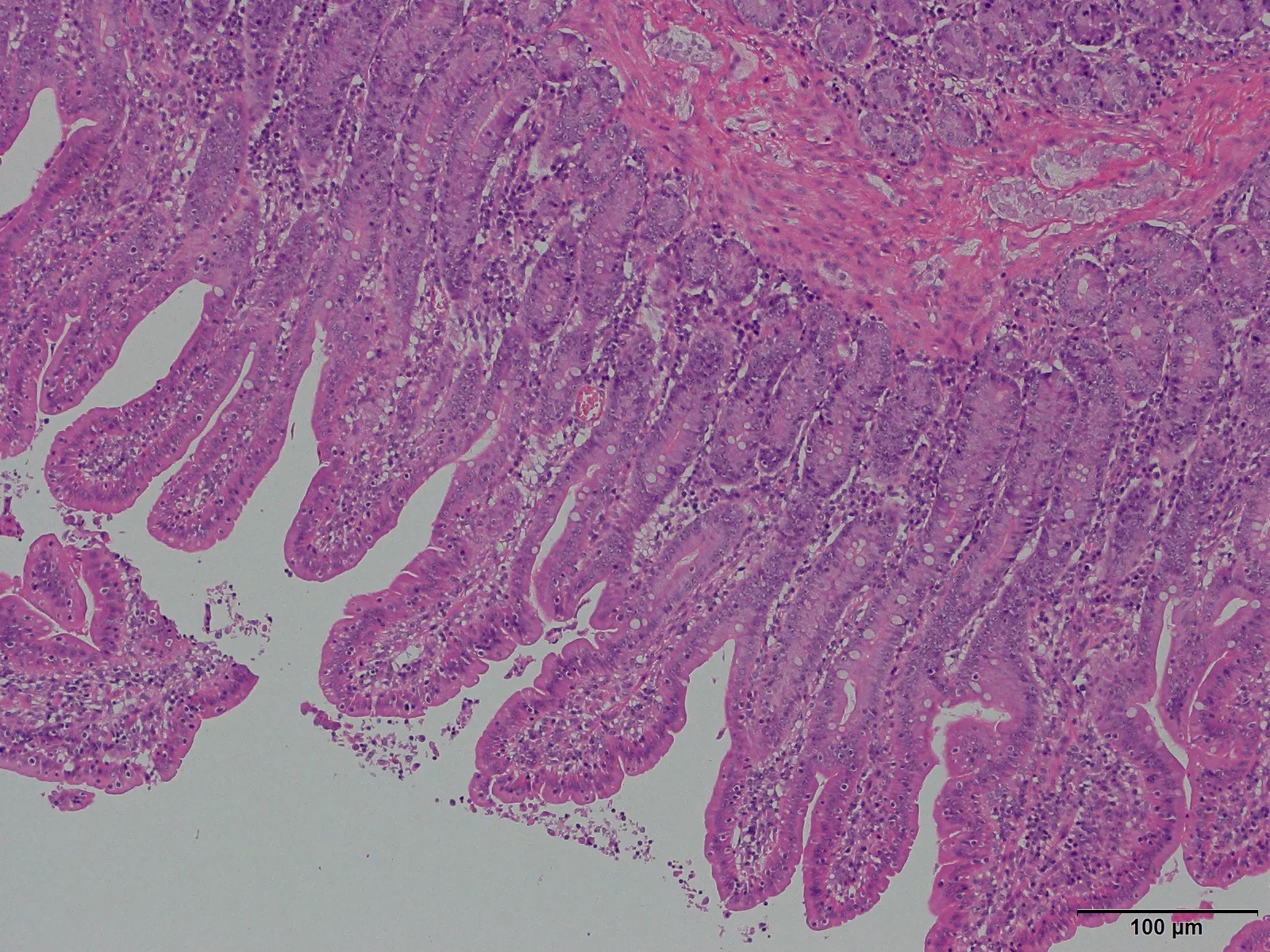

Supplement: Supplementary file 1 [file animals-16-01400-s001.zip › 2. Jejunum/120 mg kg CEO group/Jejunum-2-6.webp]

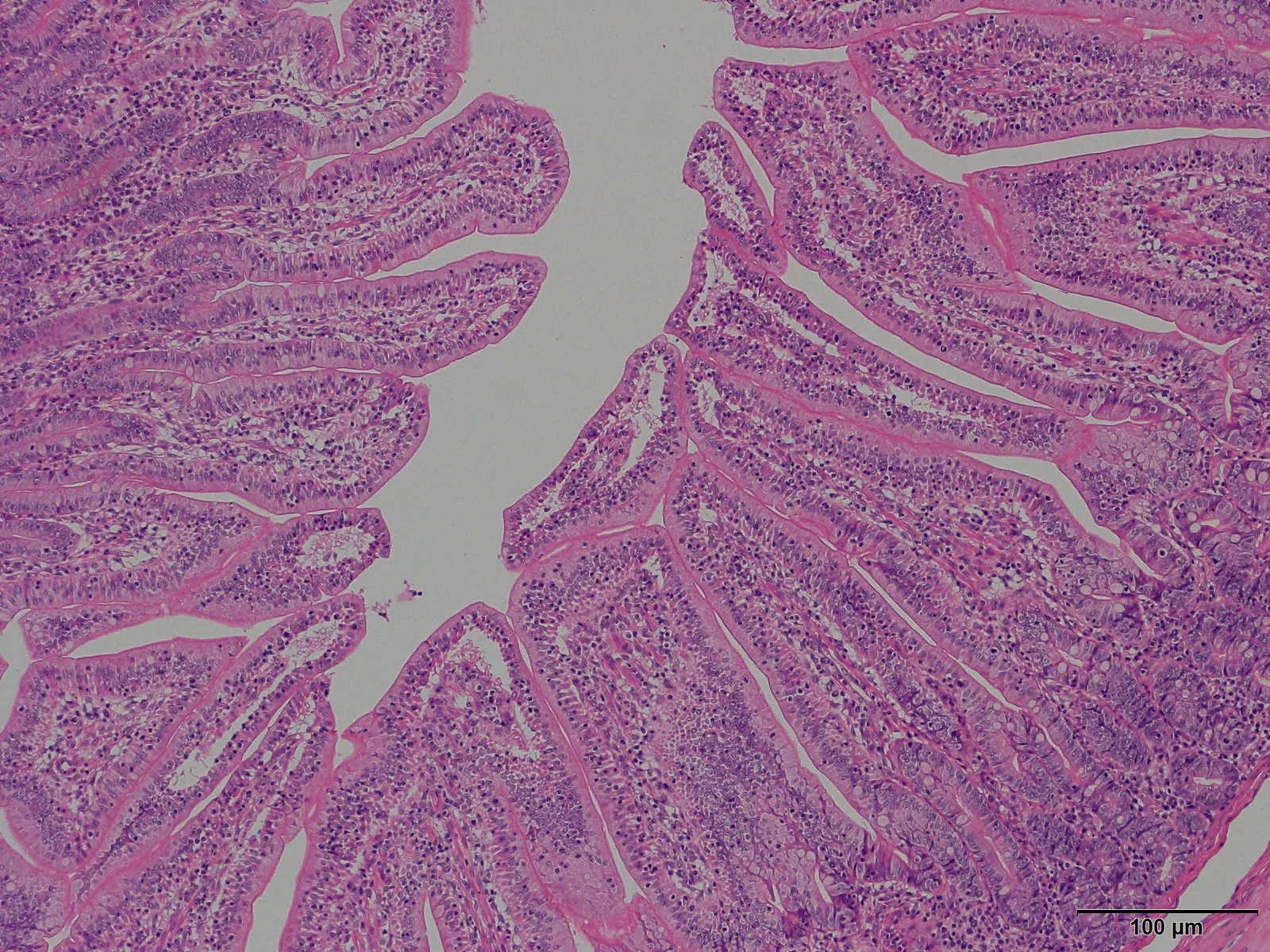

Supplement: Supplementary file 1 [file animals-16-01400-s001.zip › 2. Jejunum/120 mg kg CEO group/Jejunum-2-7.webp]

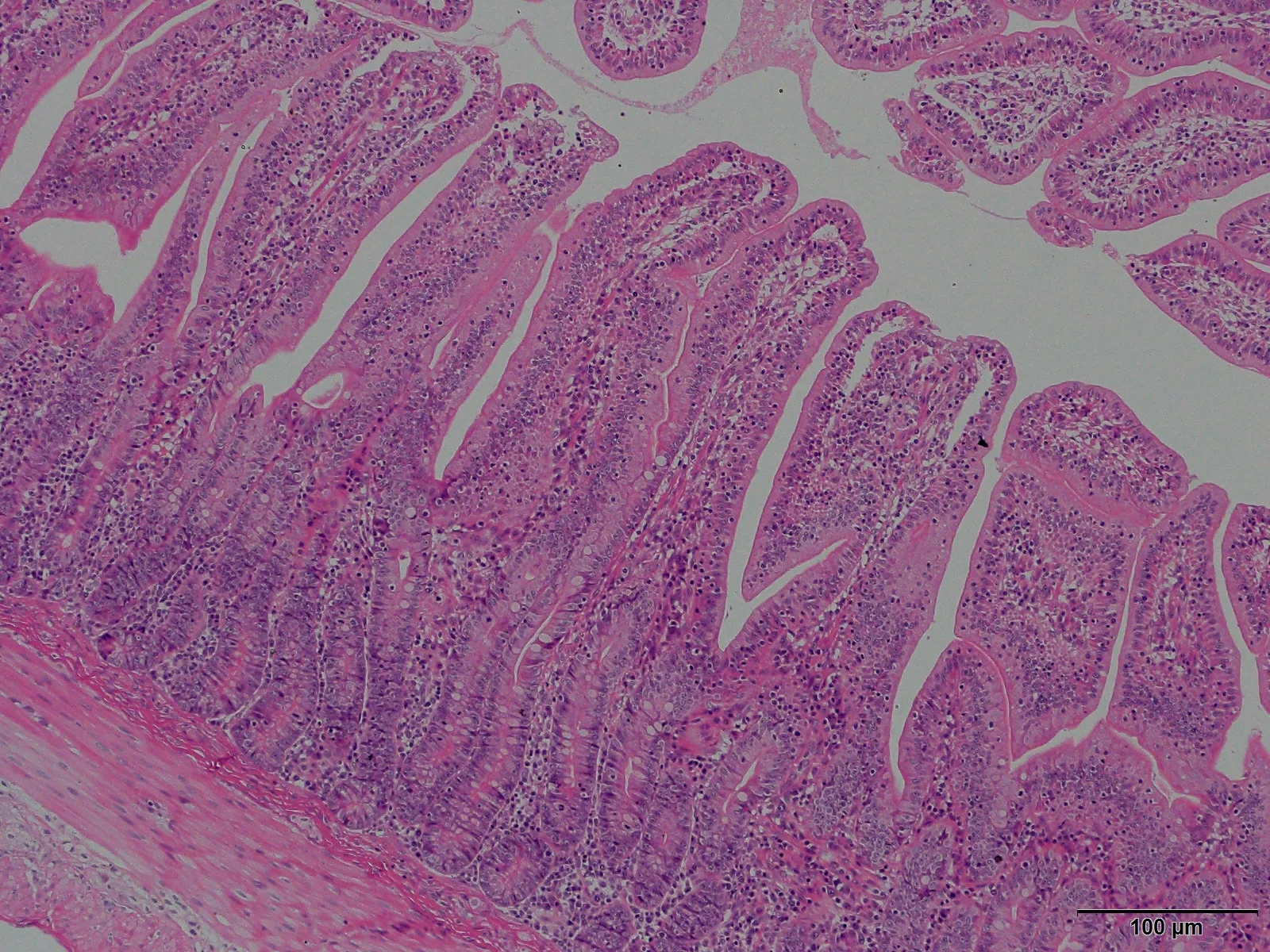

Supplement: Supplementary file 1 [file animals-16-01400-s001.zip › 2. Jejunum/120 mg kg CEO group/Jejunum-2-8.webp]

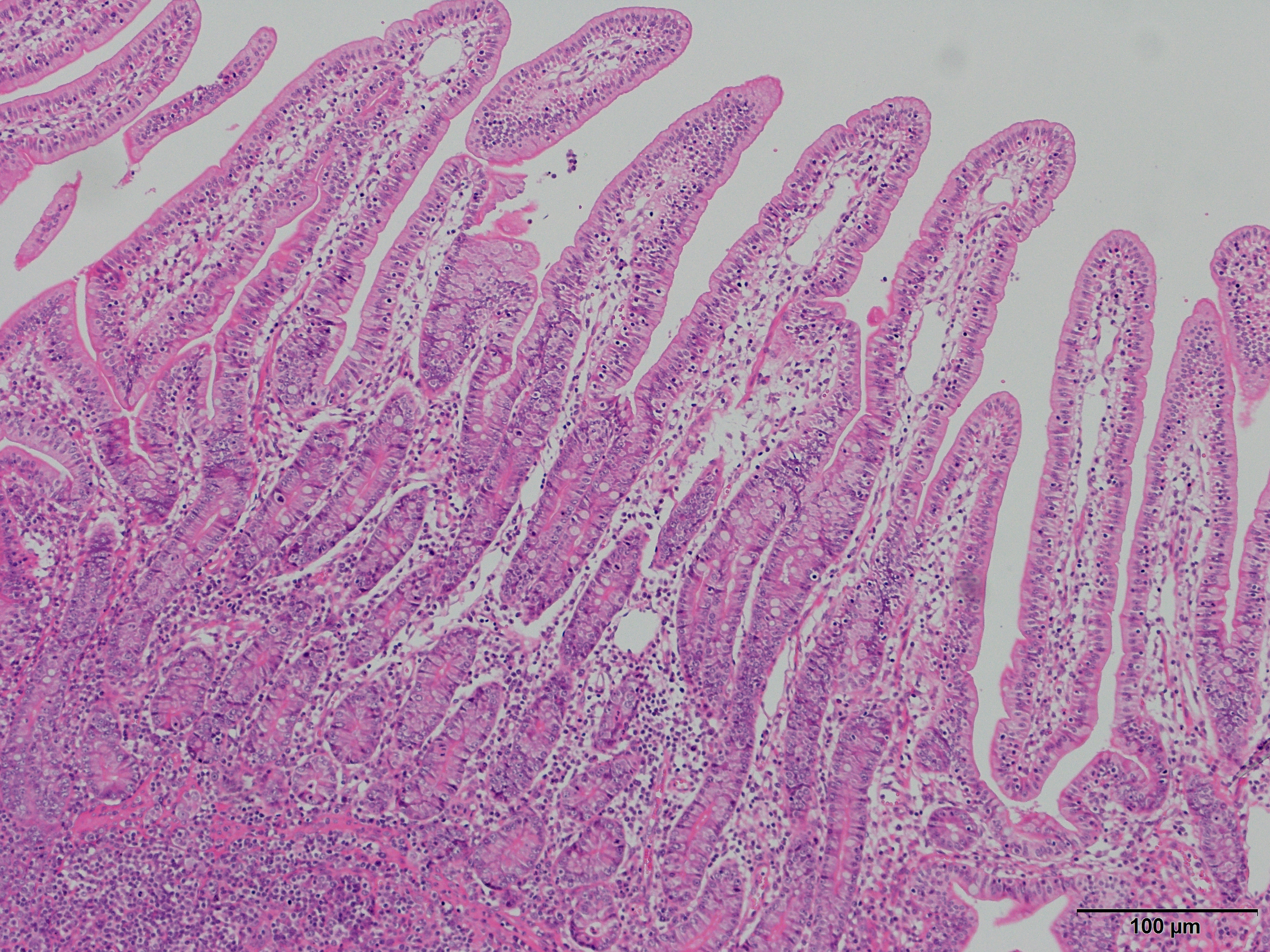

Supplement: Supplementary file 1 [file animals-16-01400-s001.zip › 2. Jejunum/180 mg kg CEO group/Jejunum-3-1.jpg]

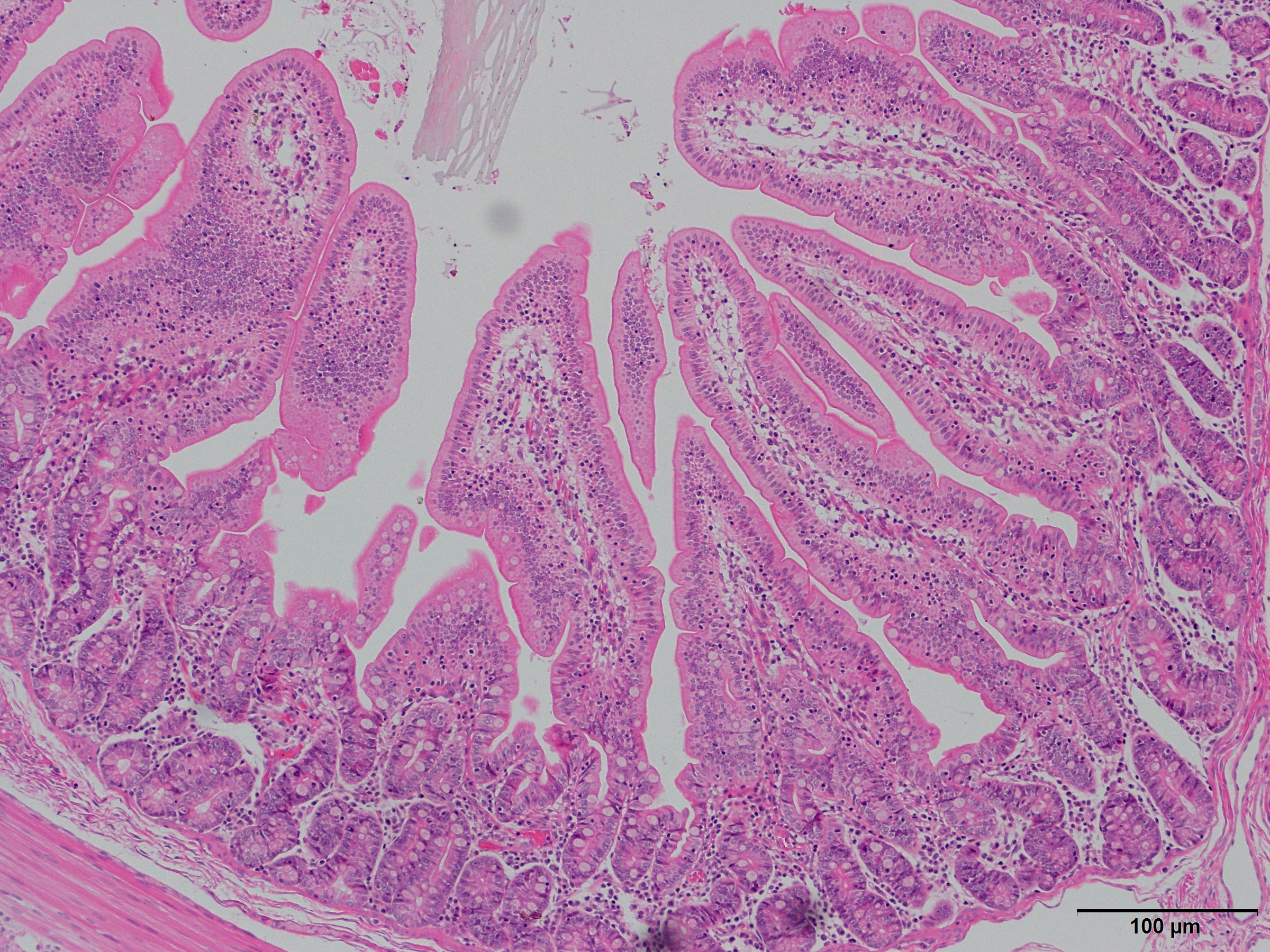

Supplement: Supplementary file 1 [file animals-16-01400-s001.zip › 2. Jejunum/180 mg kg CEO group/Jejunum-3-2.jpg]

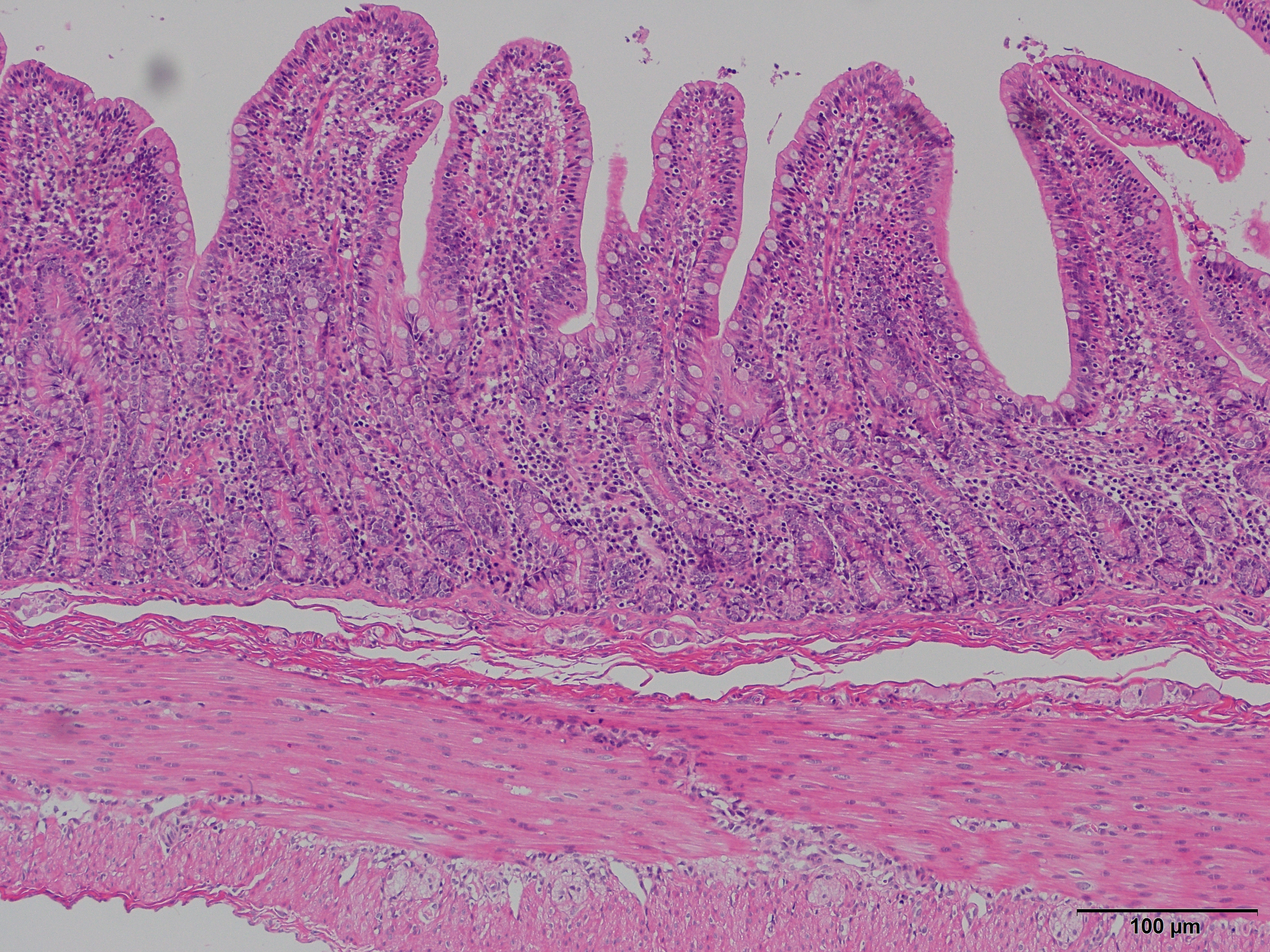

Supplement: Supplementary file 1 [file animals-16-01400-s001.zip › 2. Jejunum/180 mg kg CEO group/Jejunum-3-3.jpg]

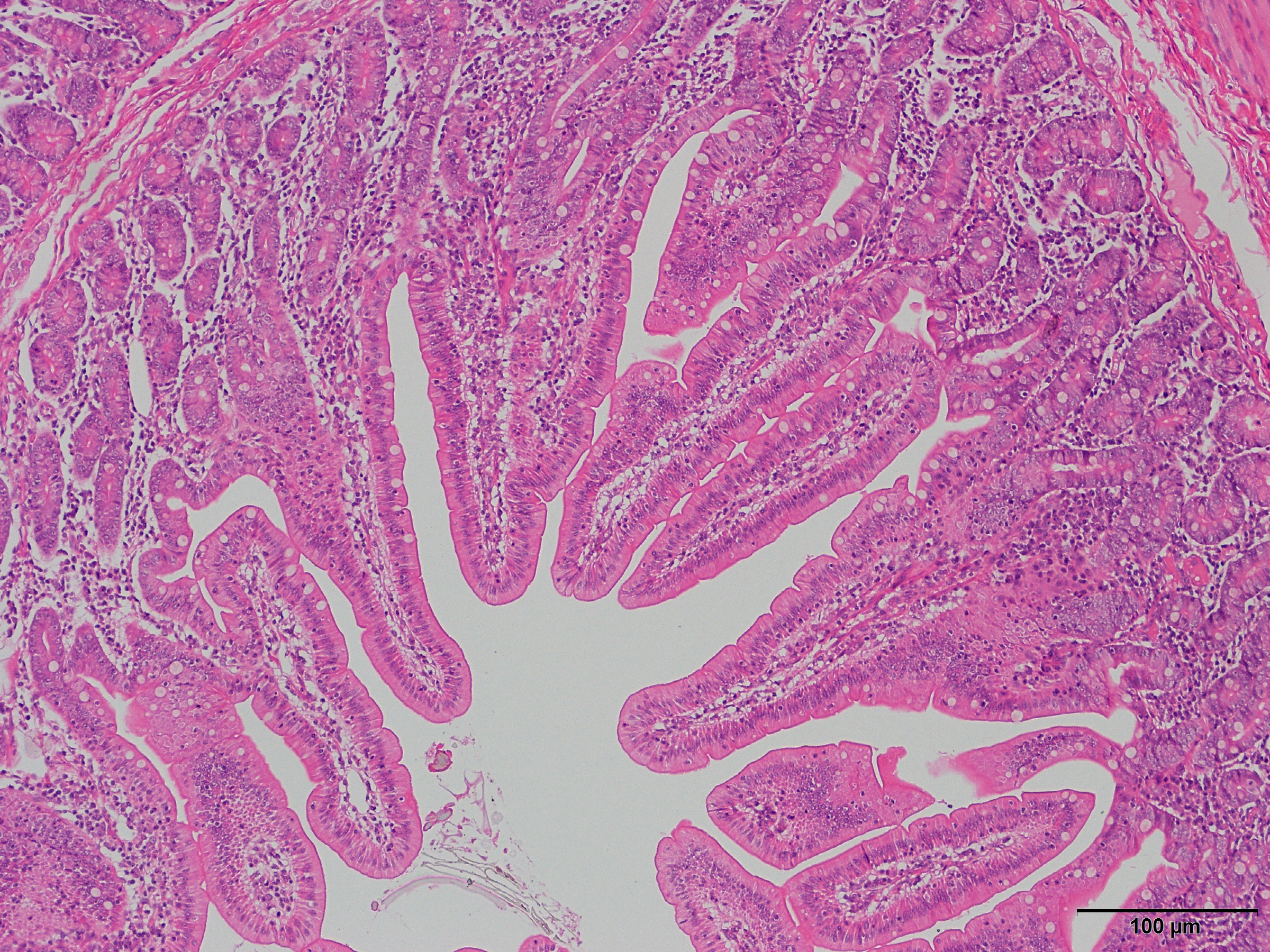

Supplement: Supplementary file 1 [file animals-16-01400-s001.zip › 2. Jejunum/180 mg kg CEO group/Jejunum-3-4.jpg]

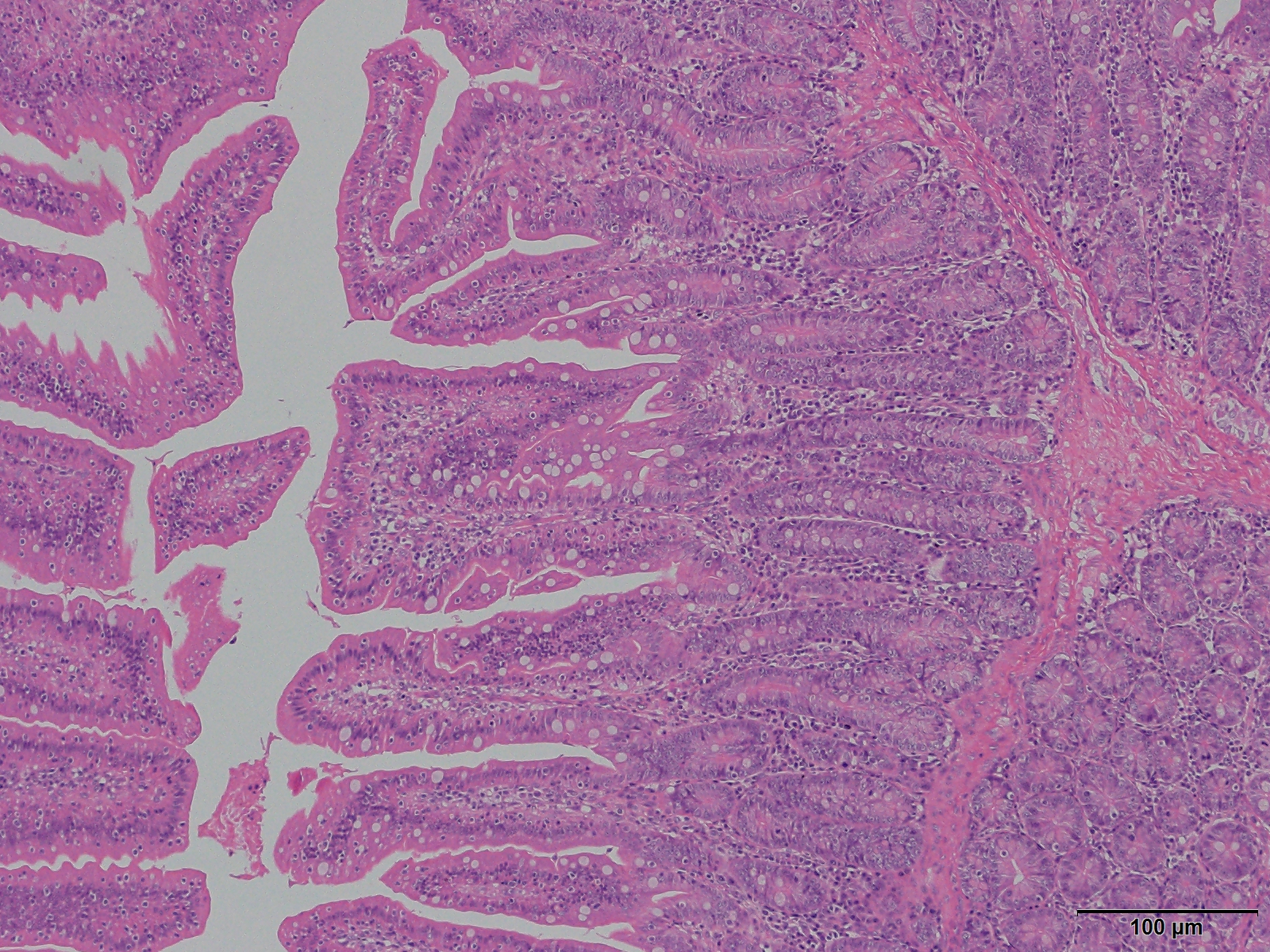

Supplement: Supplementary file 1 [file animals-16-01400-s001.zip › 2. Jejunum/180 mg kg CEO group/Jejunum-3-5.jpg]

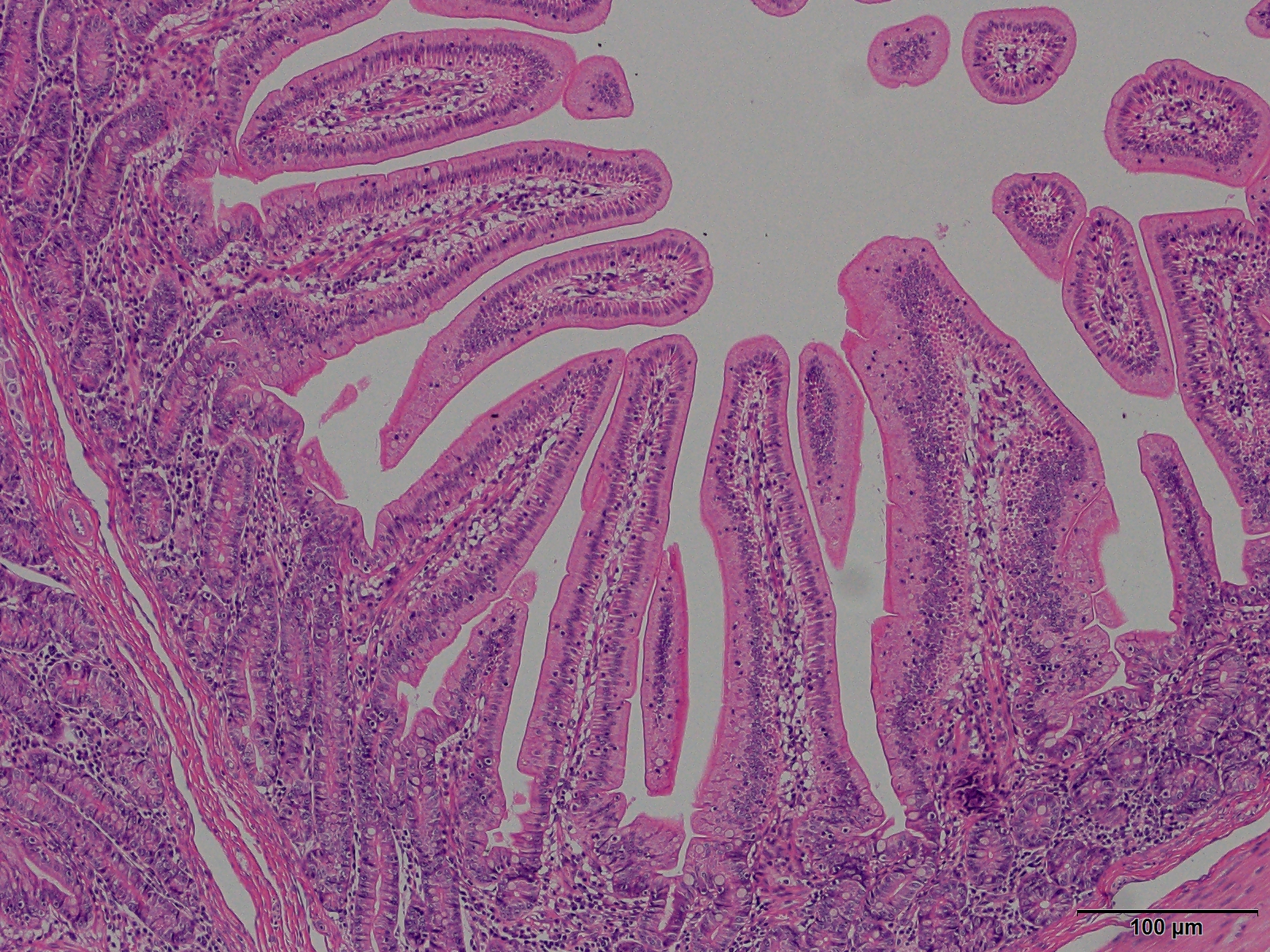

Supplement: Supplementary file 1 [file animals-16-01400-s001.zip › 2. Jejunum/180 mg kg CEO group/Jejunum-3-6.jpg]

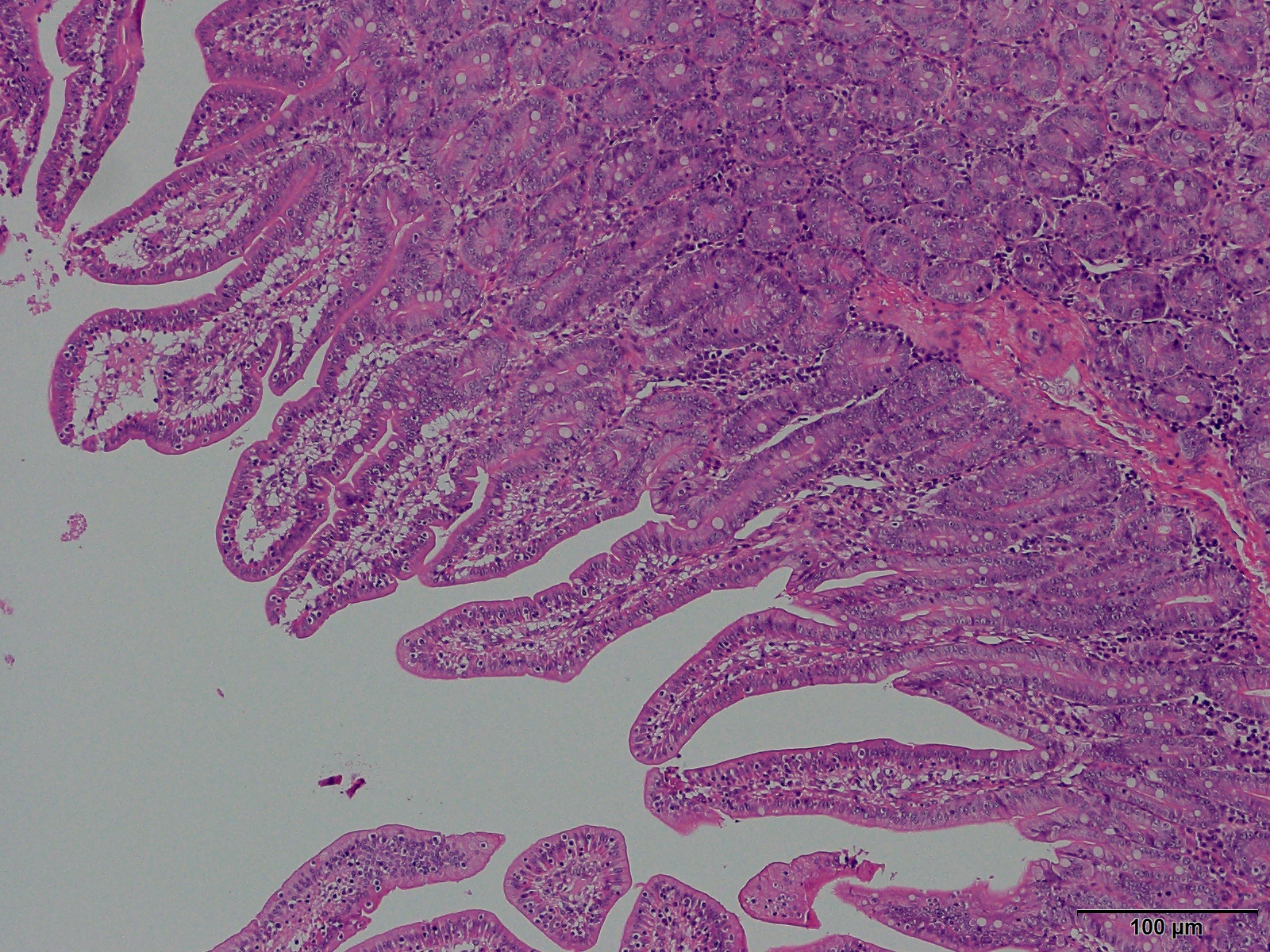

Supplement: Supplementary file 1 [file animals-16-01400-s001.zip › 2. Jejunum/180 mg kg CEO group/Jejunum-3-7.jpg]

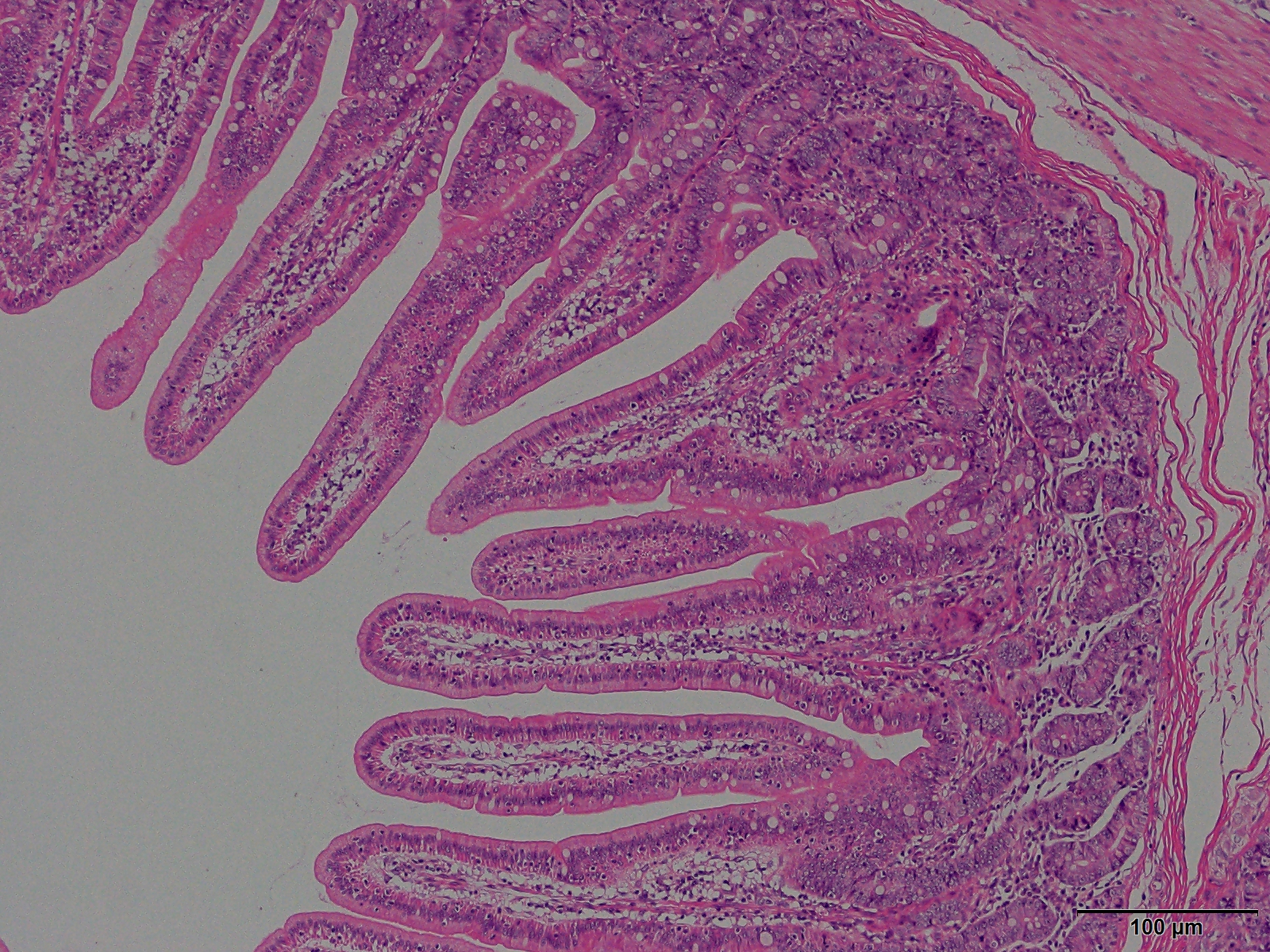

Supplement: Supplementary file 1 [file animals-16-01400-s001.zip › 2. Jejunum/180 mg kg CEO group/Jejunum-3-8-Figure 3A.jpg]

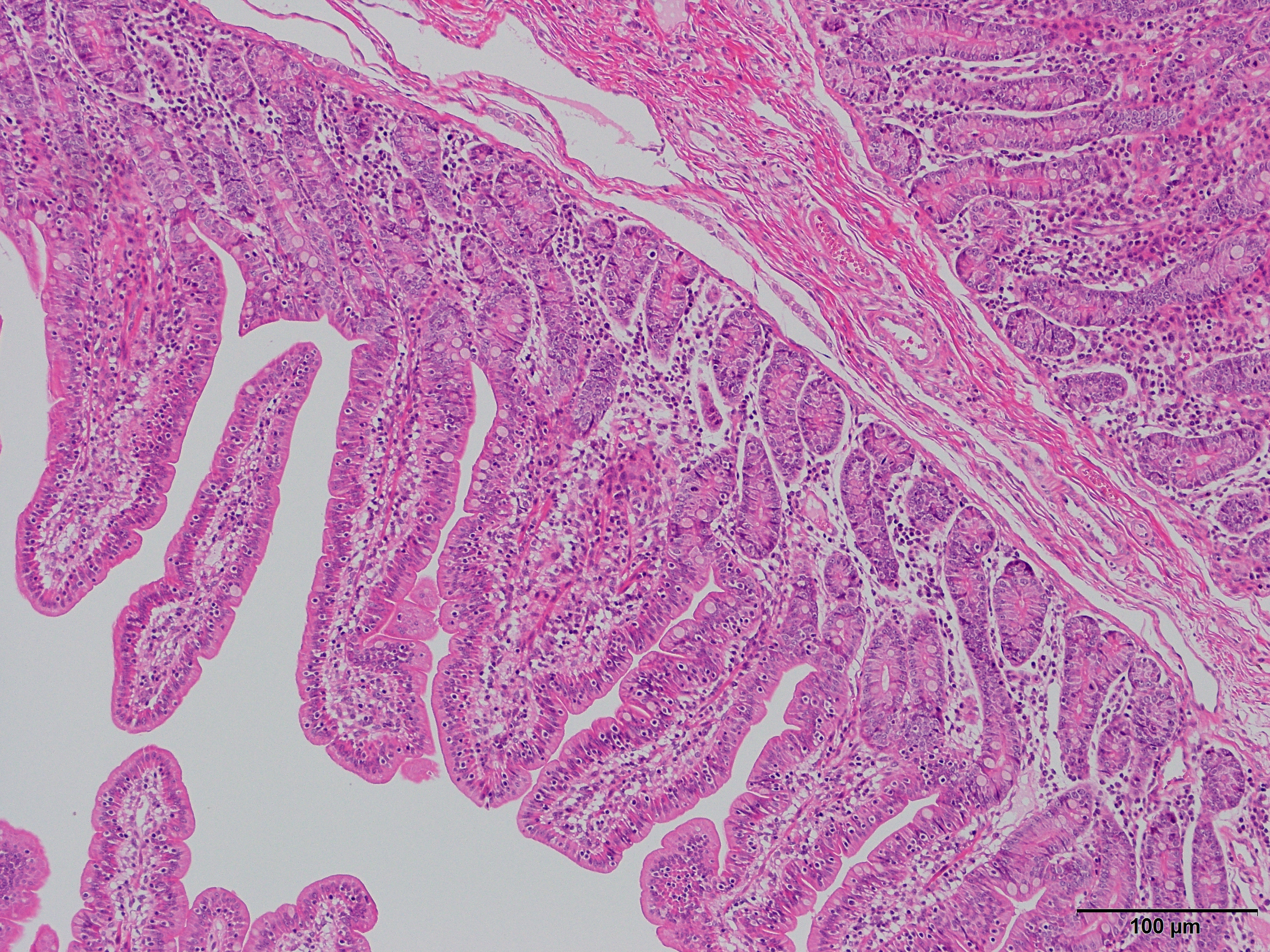

Supplement: Supplementary file 1 [file animals-16-01400-s001.zip › 2. Jejunum/240 mg kg CEO group/Jejunum-4-1.jpg]

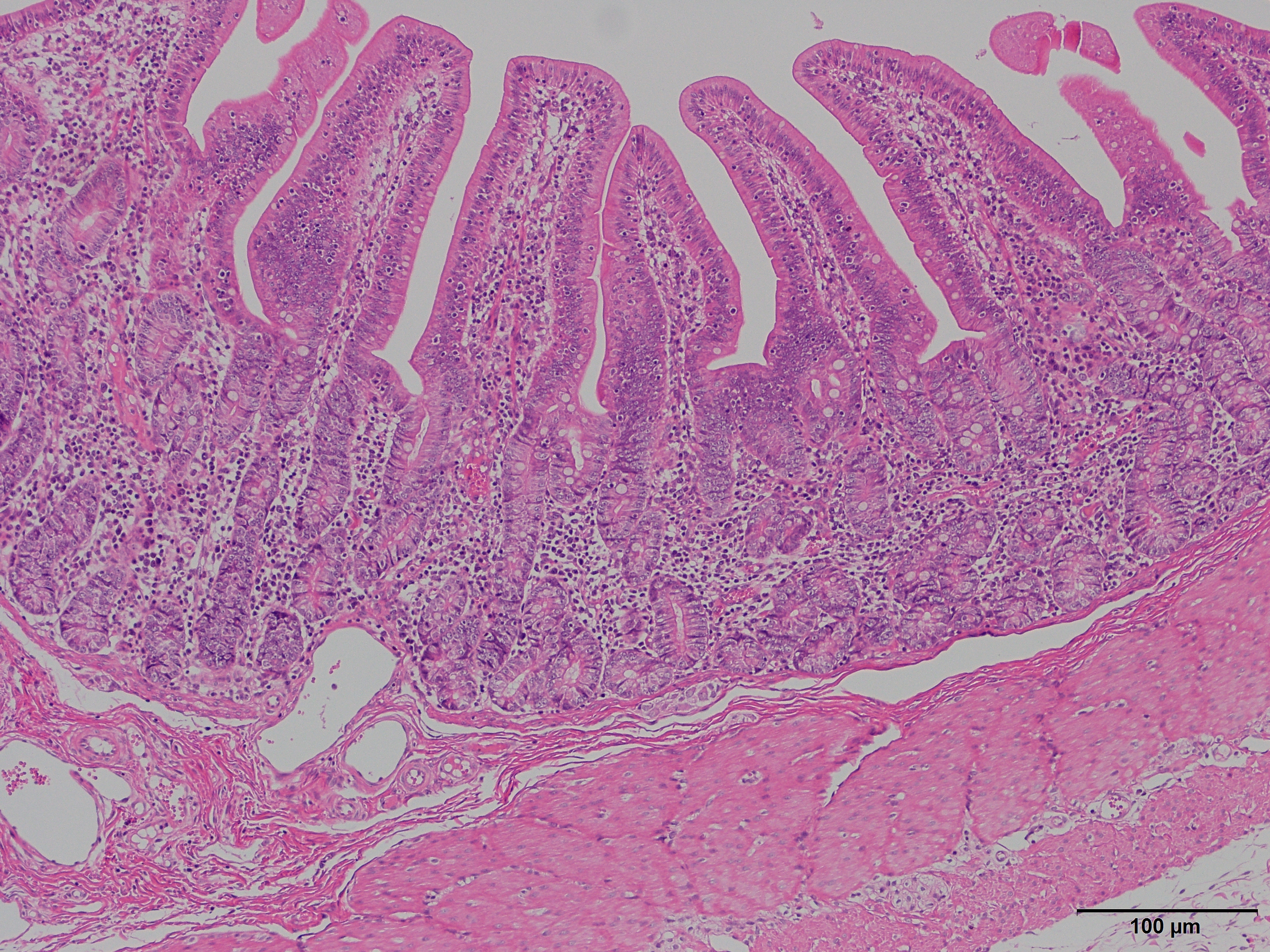

Supplement: Supplementary file 1 [file animals-16-01400-s001.zip › 2. Jejunum/240 mg kg CEO group/Jejunum-4-2.jpg]

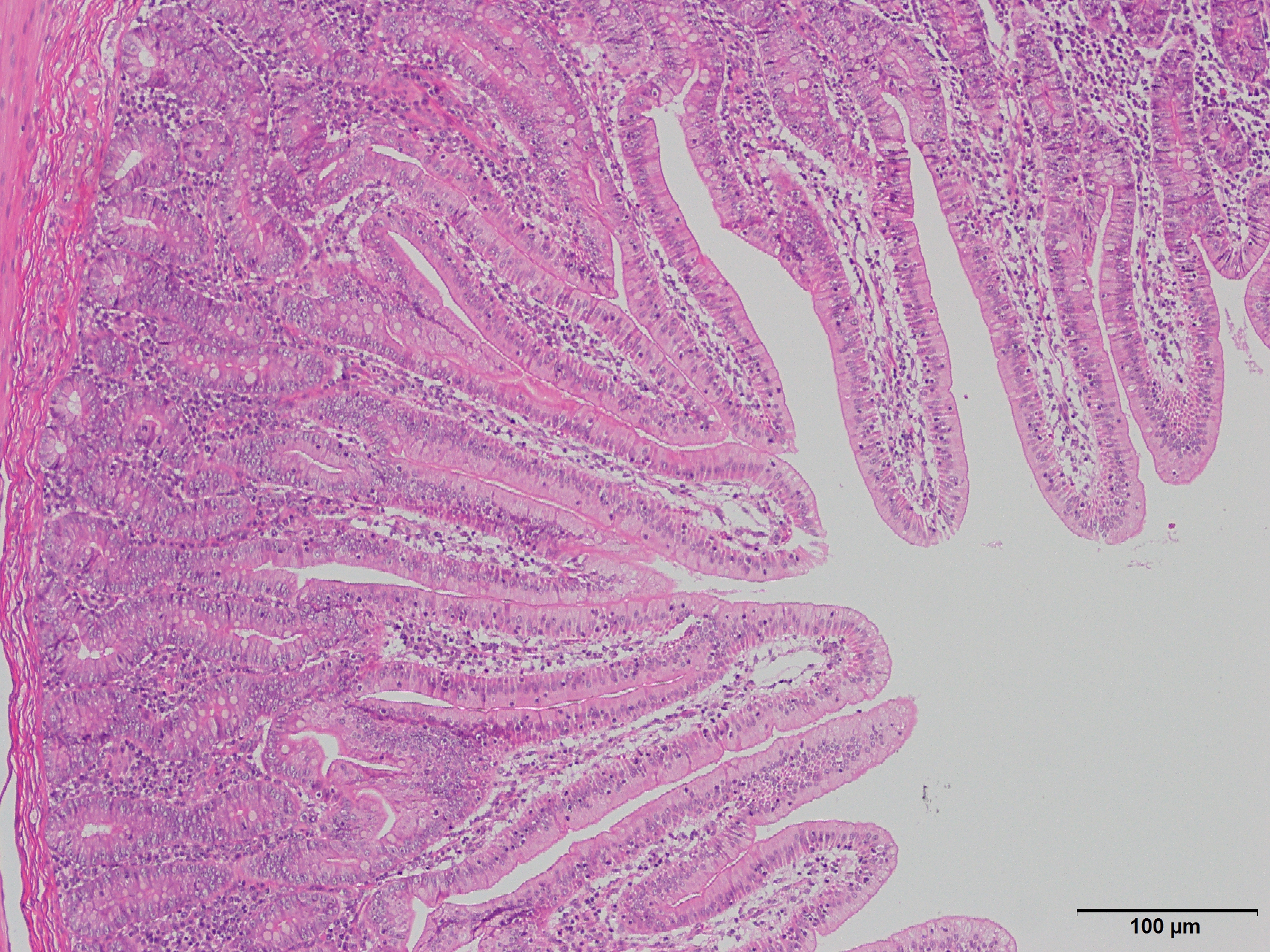

Supplement: Supplementary file 1 [file animals-16-01400-s001.zip › 2. Jejunum/240 mg kg CEO group/Jejunum-4-3-Figure 3A.jpg]

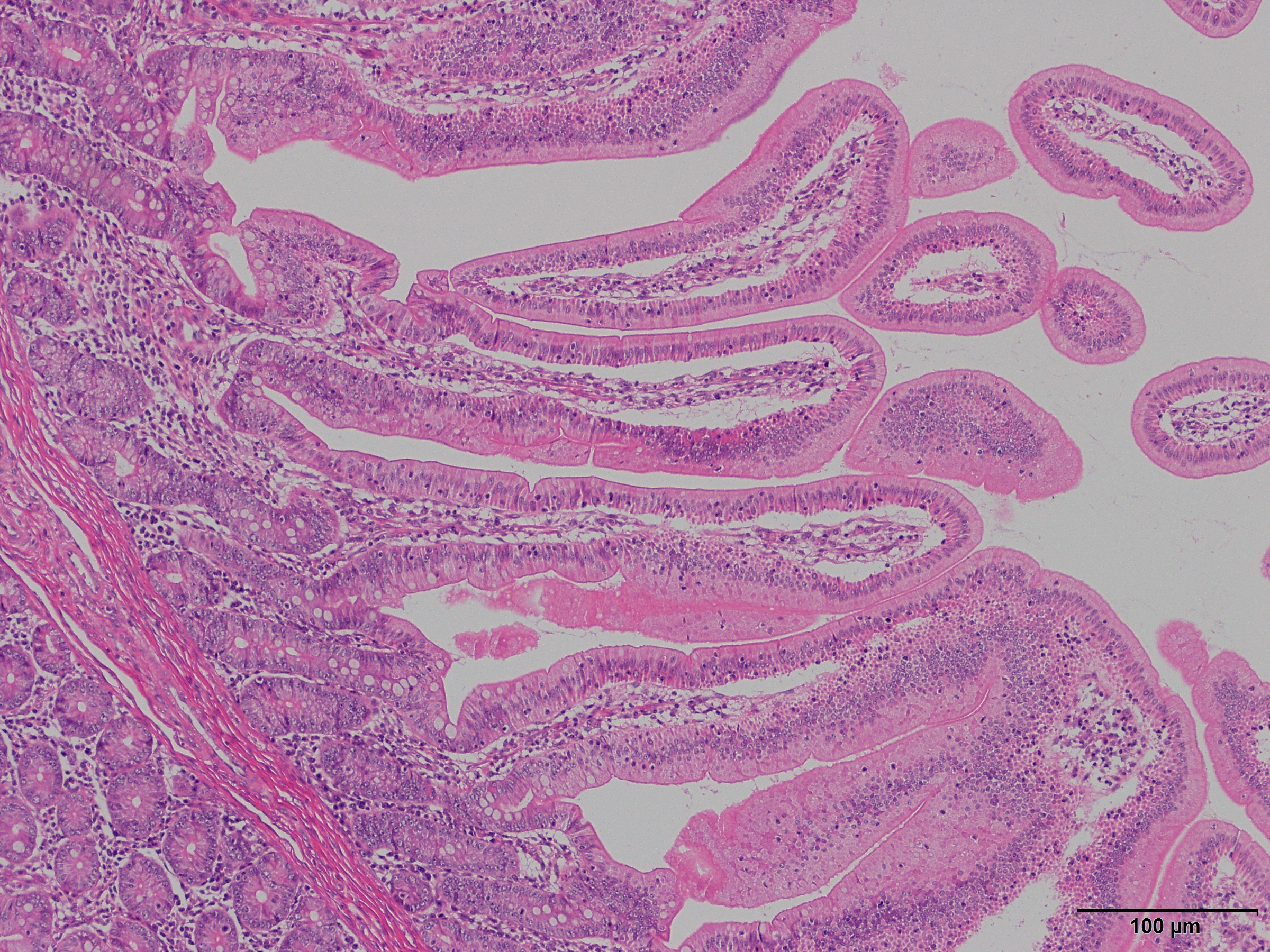

Supplement: Supplementary file 1 [file animals-16-01400-s001.zip › 2. Jejunum/240 mg kg CEO group/Jejunum-4-4.jpg]

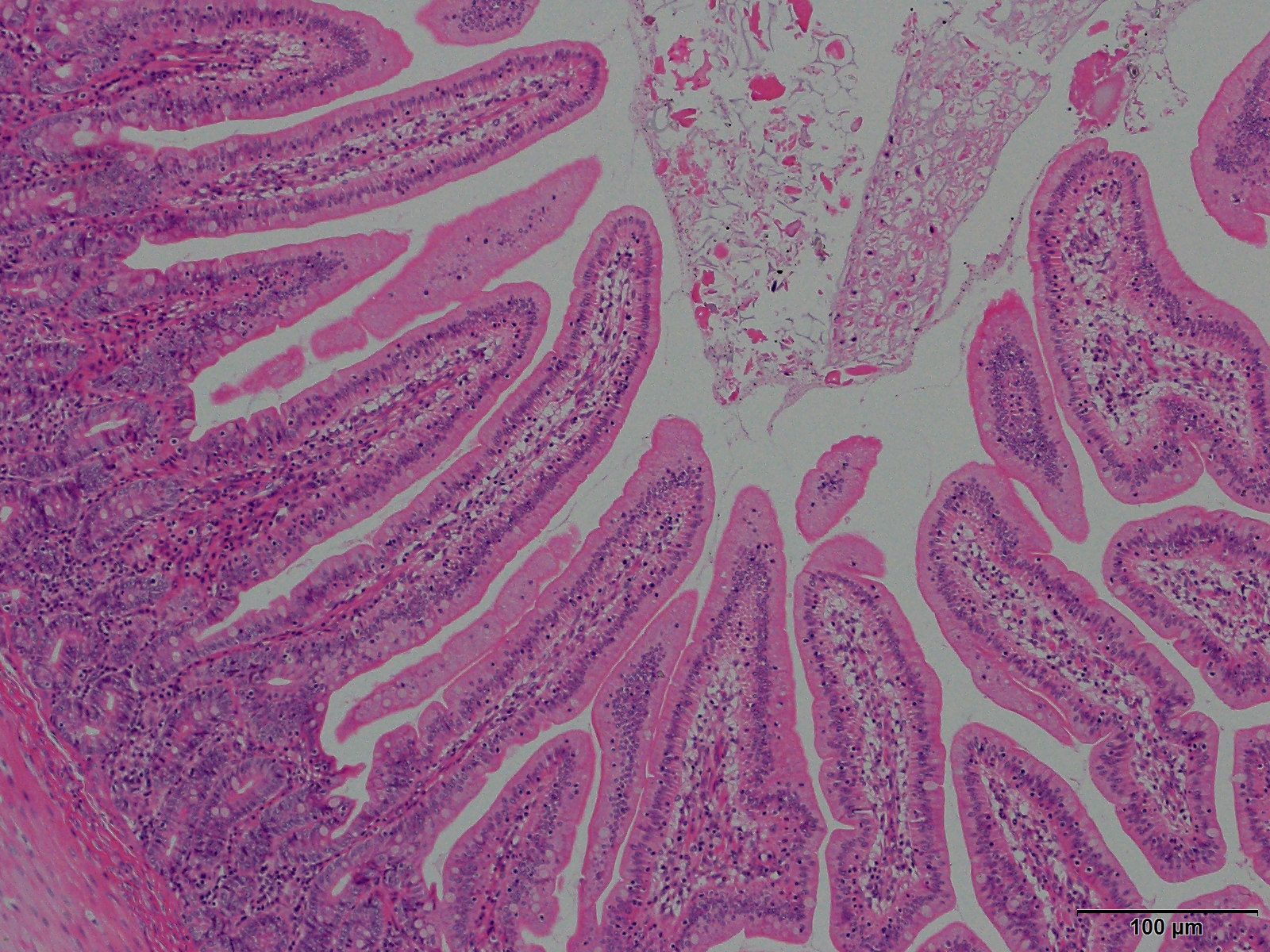

Supplement: Supplementary file 1 [file animals-16-01400-s001.zip › 2. Jejunum/240 mg kg CEO group/Jejunum-4-5.jpg]

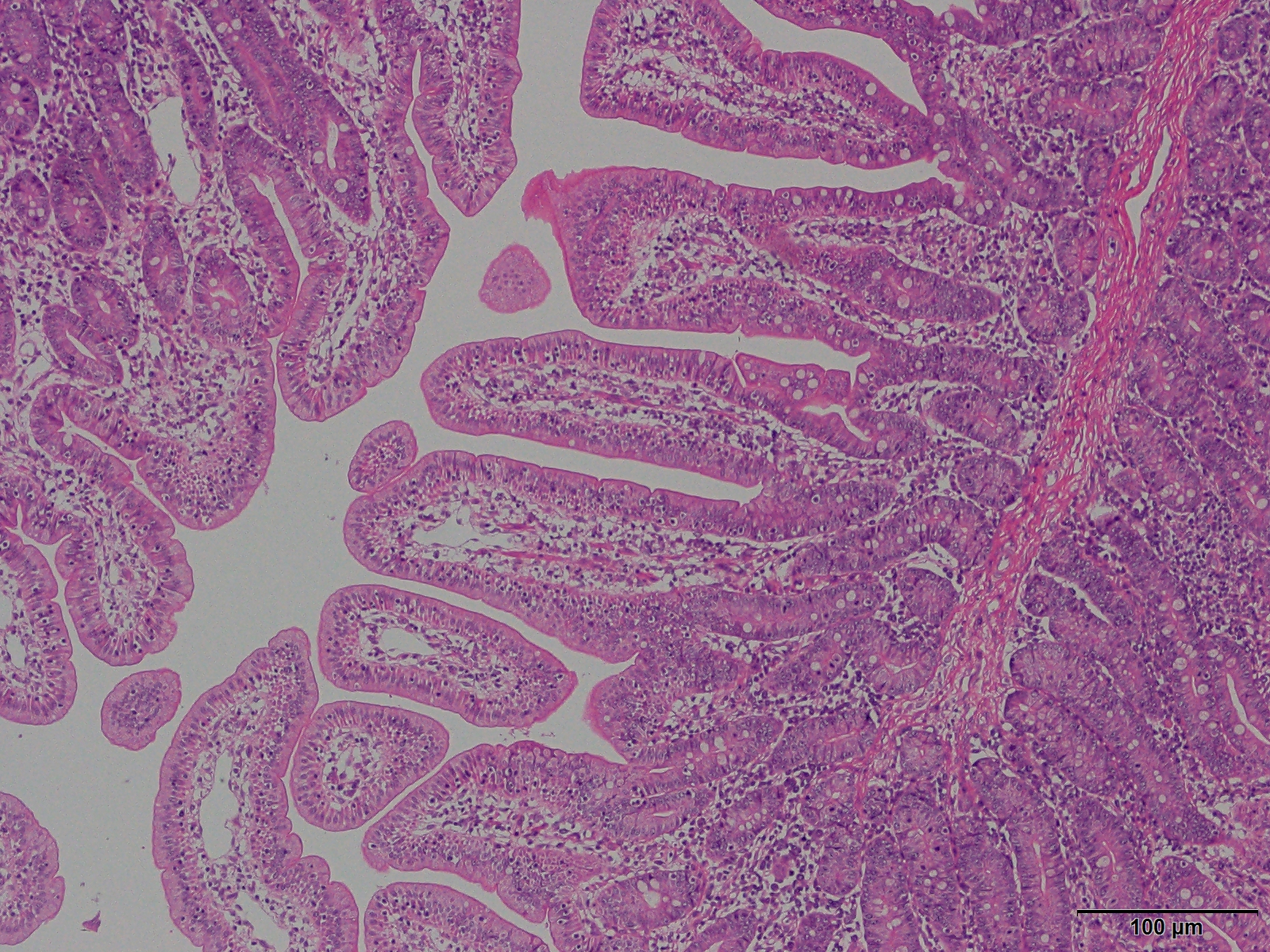

Supplement: Supplementary file 1 [file animals-16-01400-s001.zip › 2. Jejunum/240 mg kg CEO group/Jejunum-4-6.jpg]

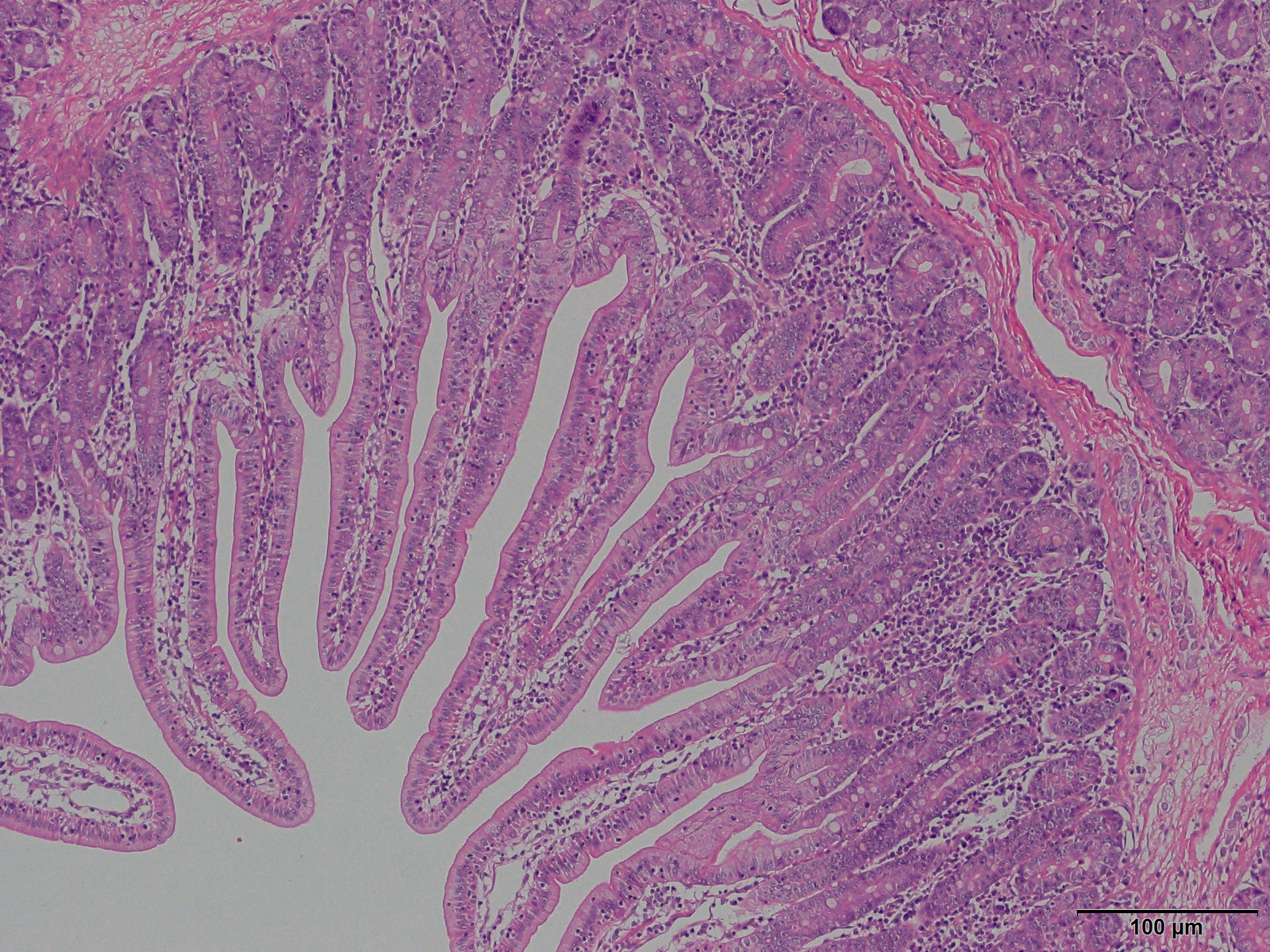

Supplement: Supplementary file 1 [file animals-16-01400-s001.zip › 2. Jejunum/240 mg kg CEO group/Jejunum-4-7.jpg]

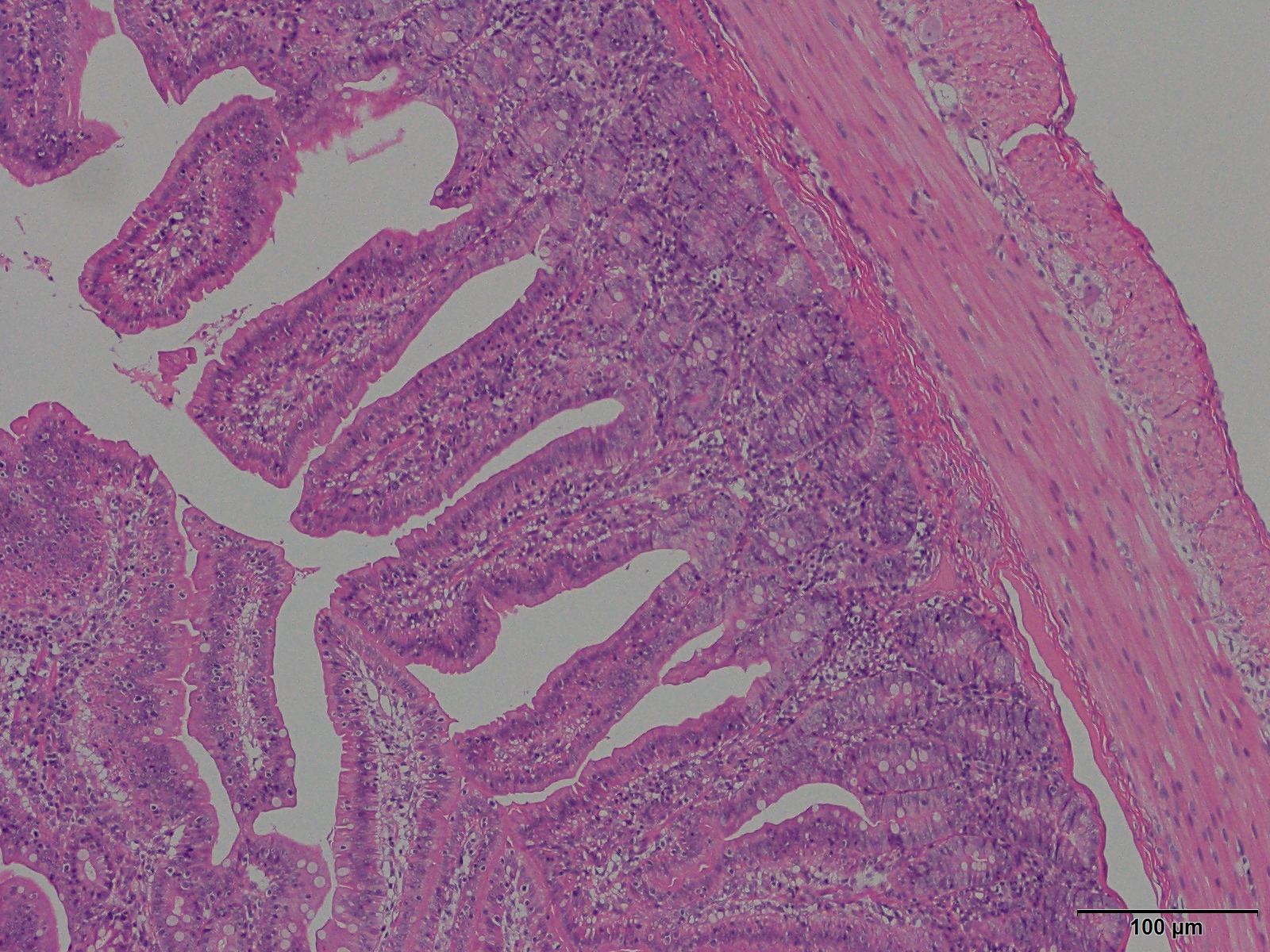

Supplement: Supplementary file 1 [file animals-16-01400-s001.zip › 2. Jejunum/240 mg kg CEO group/Jejunum-4-8.jpg]

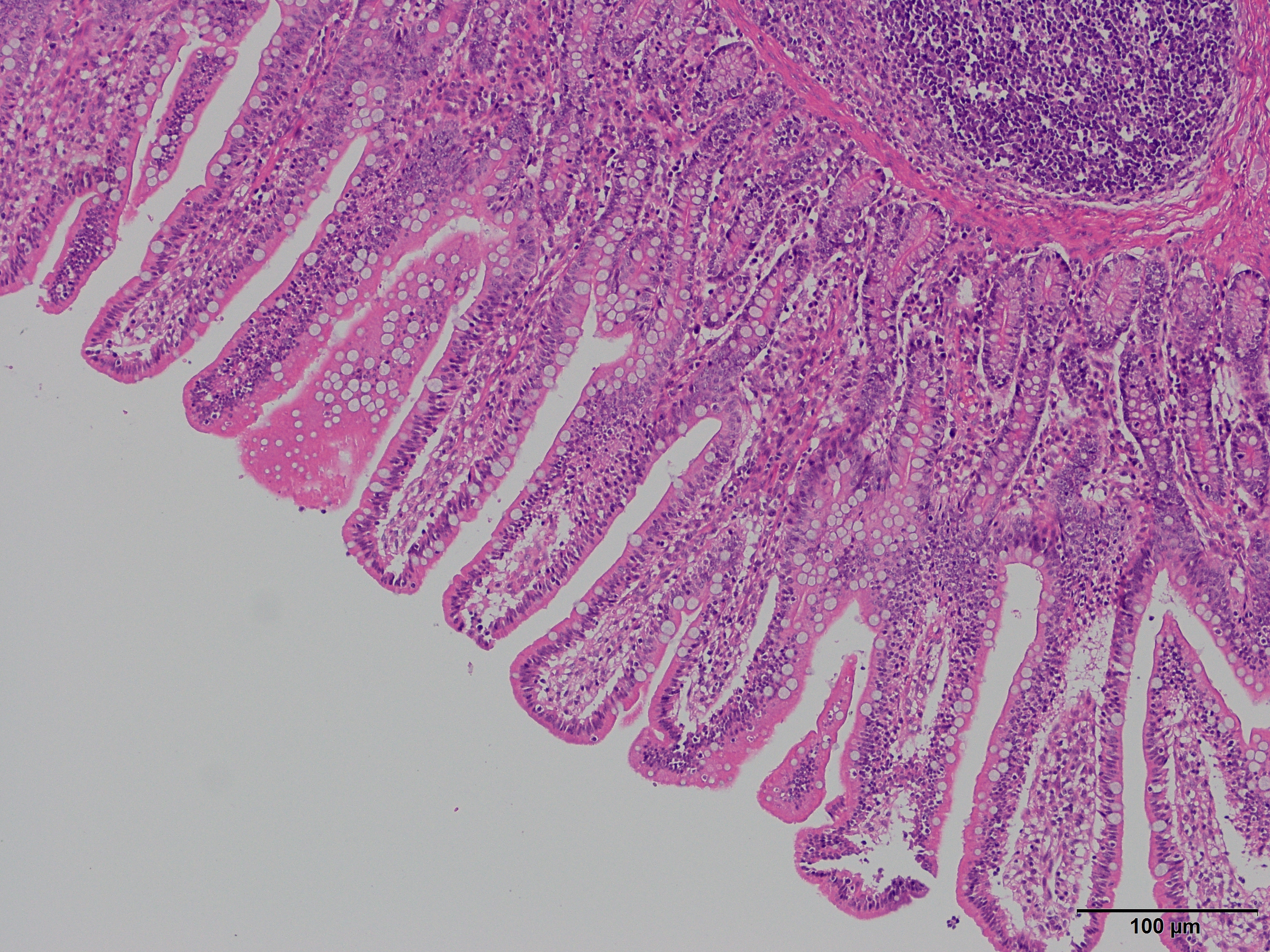

Supplement: Supplementary file 1 [file animals-16-01400-s001.zip › 3. Ileum/0 CEO group/Ileum-1-1.jpg]

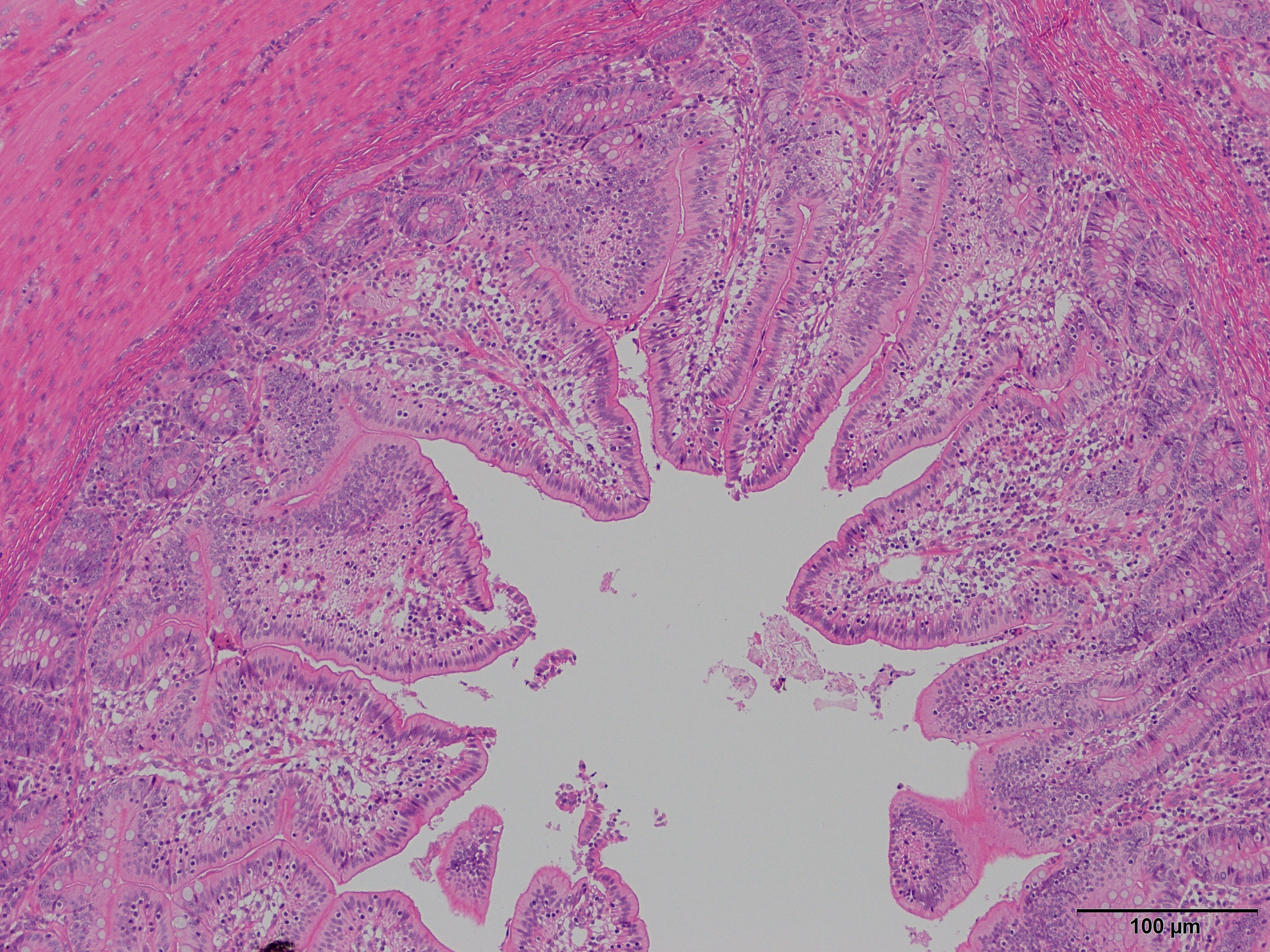

Supplement: Supplementary file 1 [file animals-16-01400-s001.zip › 3. Ileum/0 CEO group/Ileum-1-2.jpg]

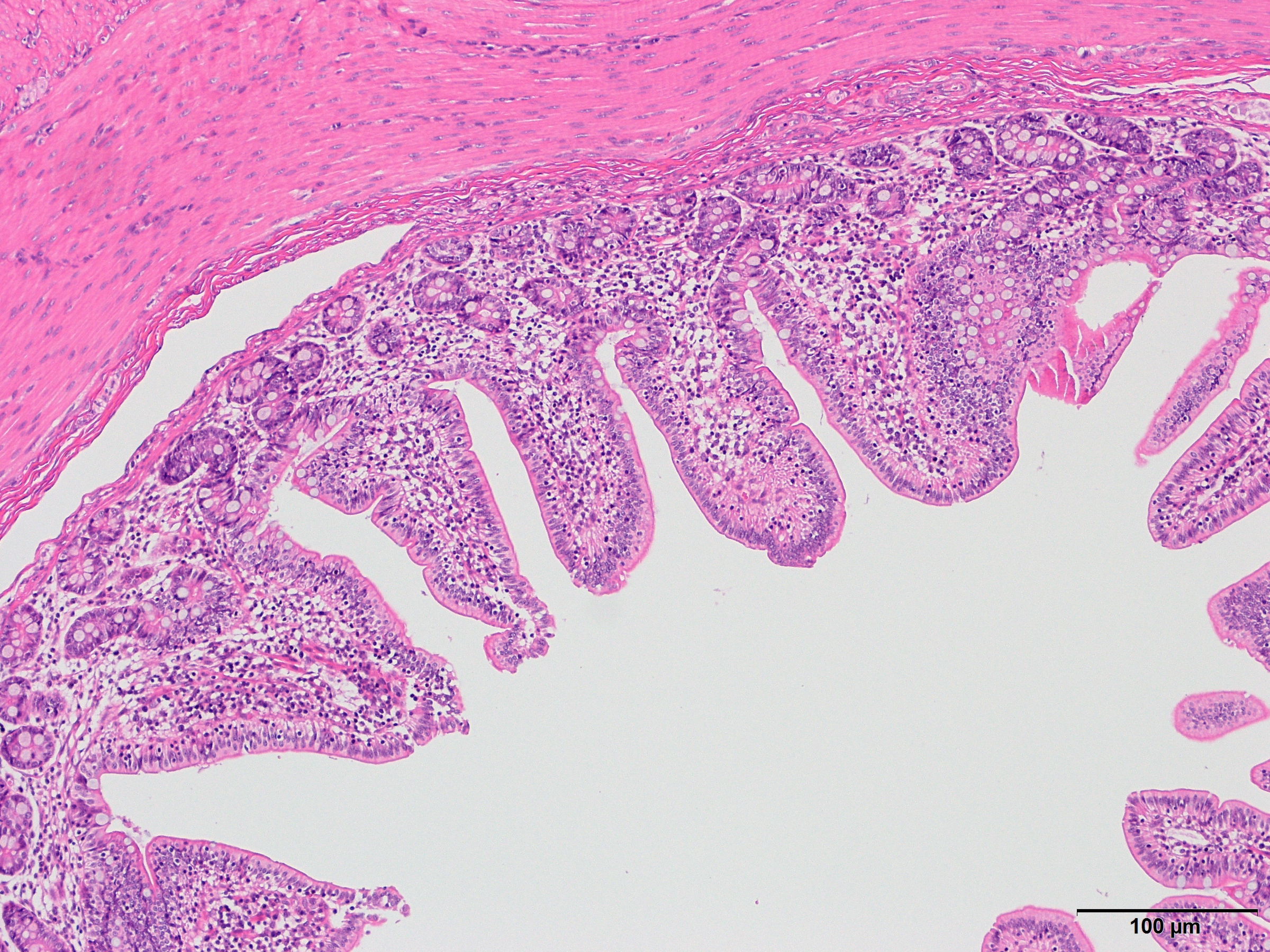

Supplement: Supplementary file 1 [file animals-16-01400-s001.zip › 3. Ileum/0 CEO group/Ileum-1-3.jpg]

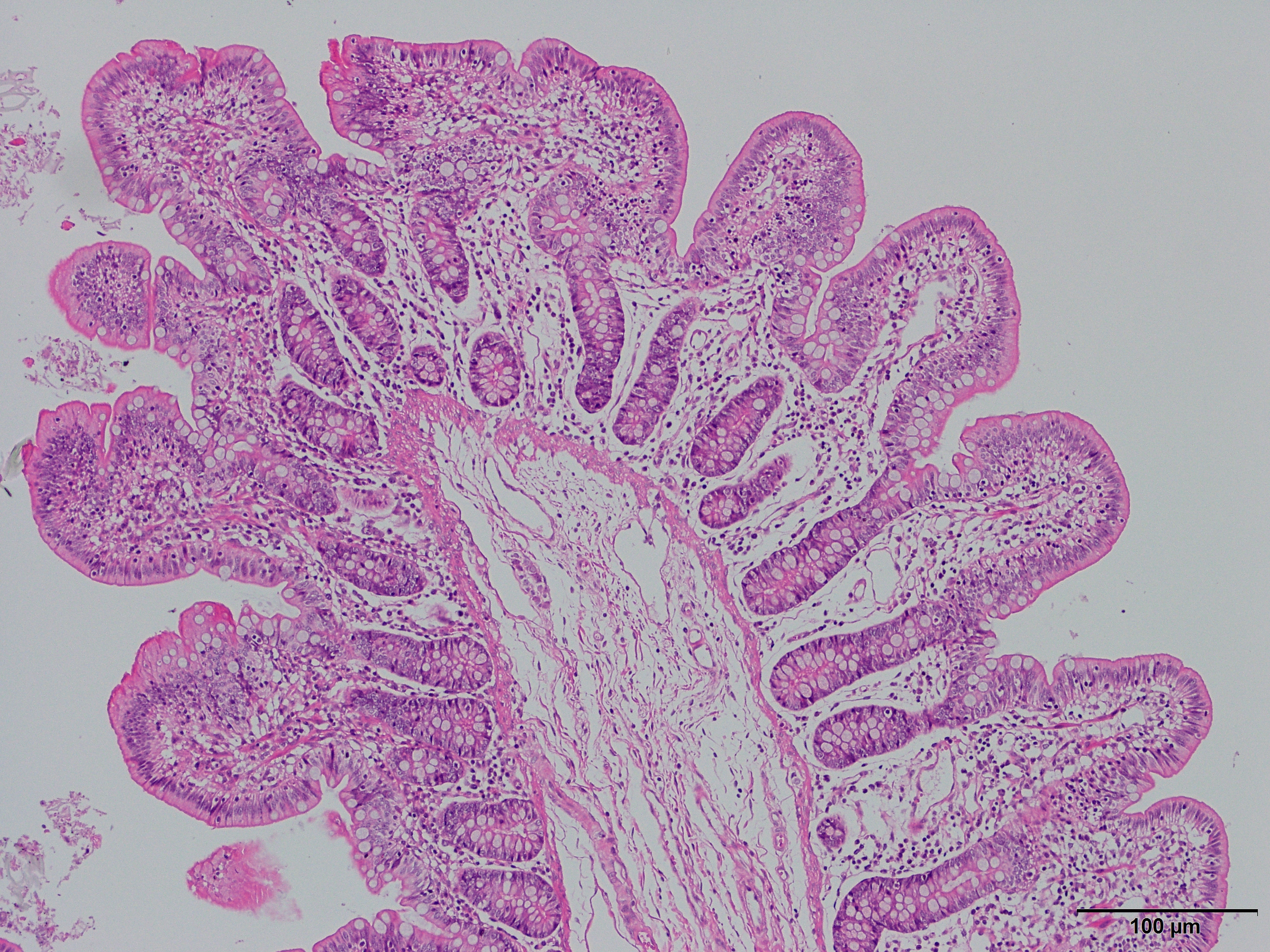

Supplement: Supplementary file 1 [file animals-16-01400-s001.zip › 3. Ileum/0 CEO group/Ileum-1-4.jpg]

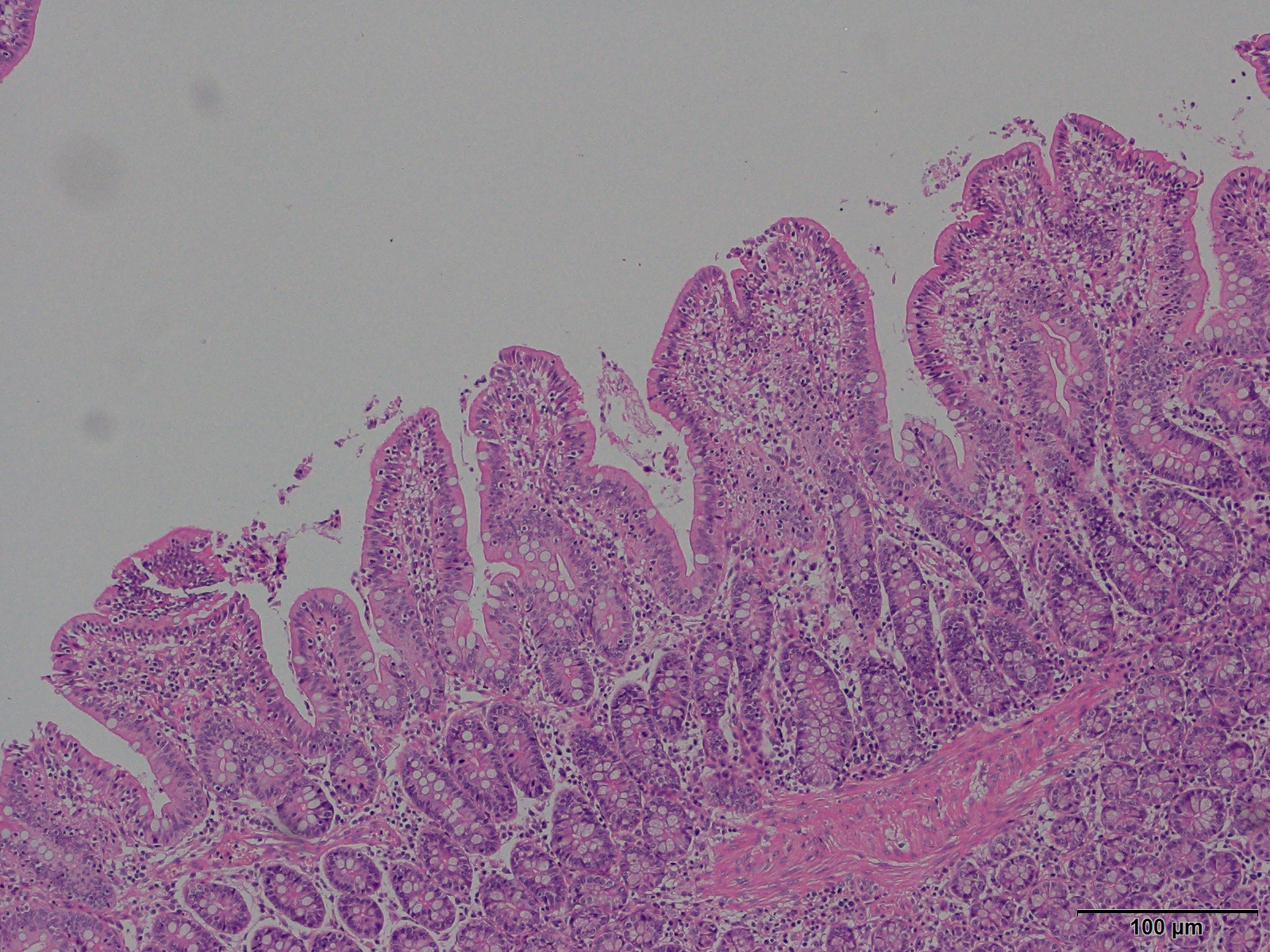

Supplement: Supplementary file 1 [file animals-16-01400-s001.zip › 3. Ileum/0 CEO group/Ileum-1-5.jpg]

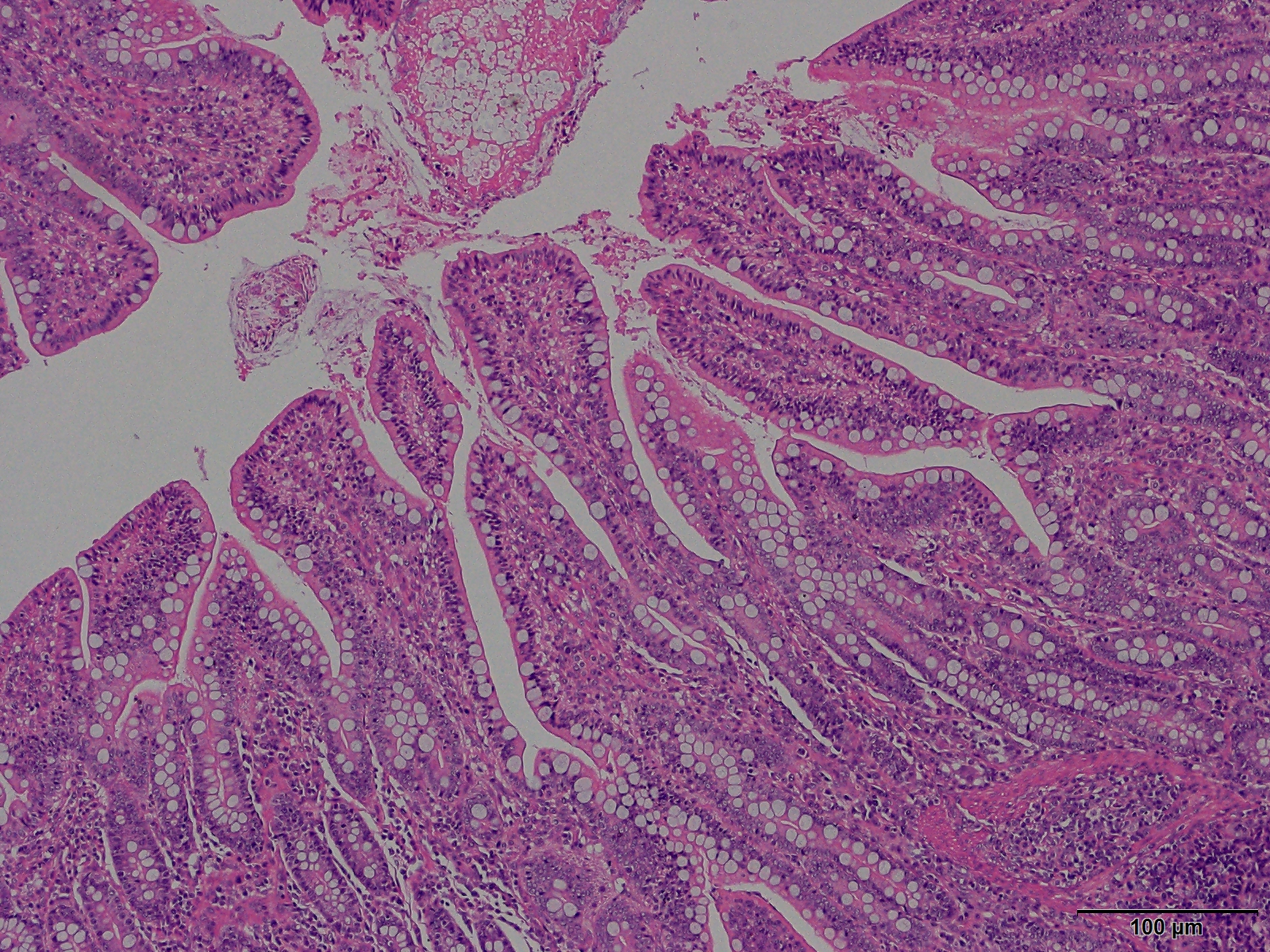

Supplement: Supplementary file 1 [file animals-16-01400-s001.zip › 3. Ileum/0 CEO group/Ileum-1-6.jpg]

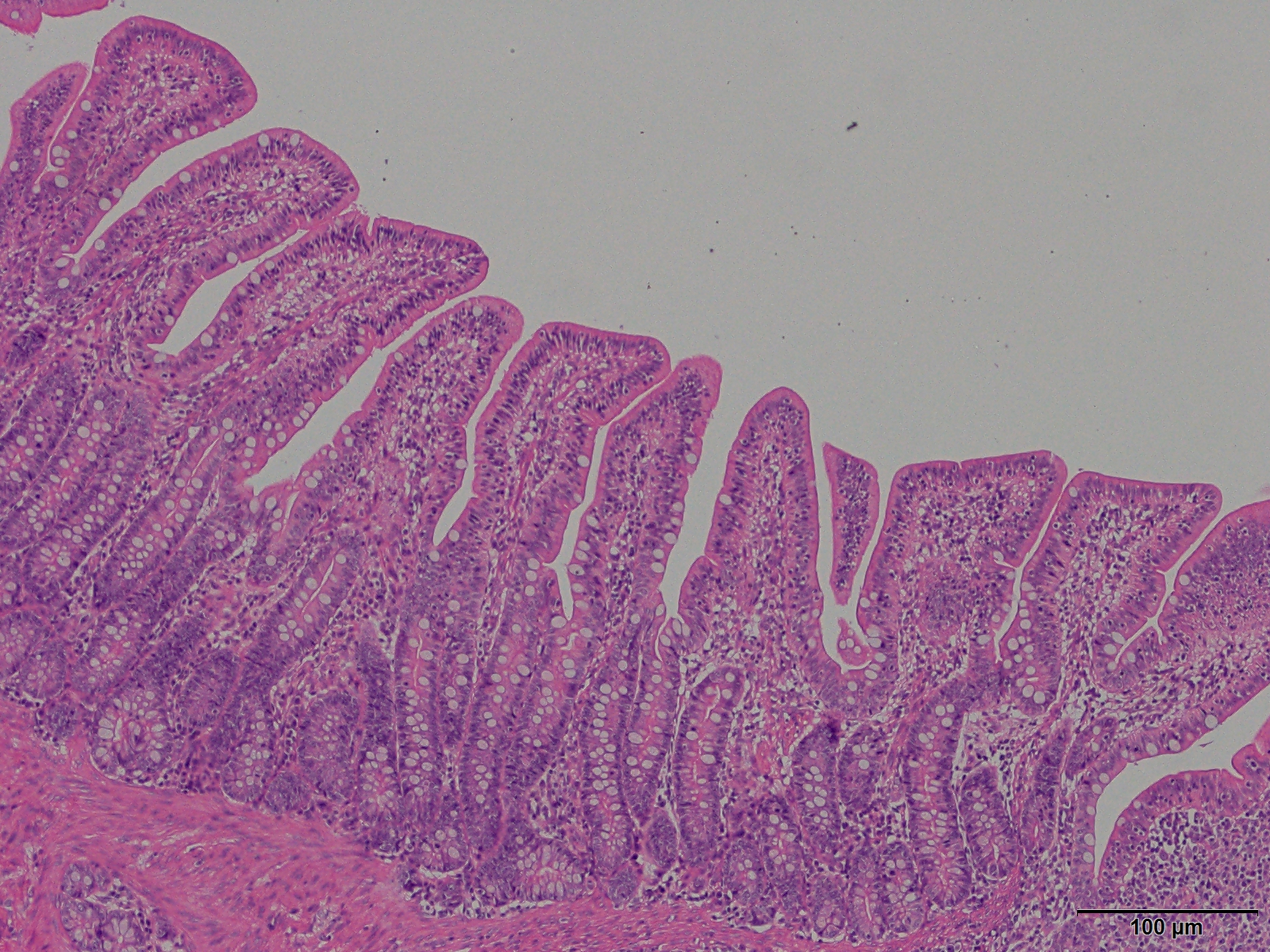

Supplement: Supplementary file 1 [file animals-16-01400-s001.zip › 3. Ileum/0 CEO group/Ileum-1-7-Figure 3A.jpg]

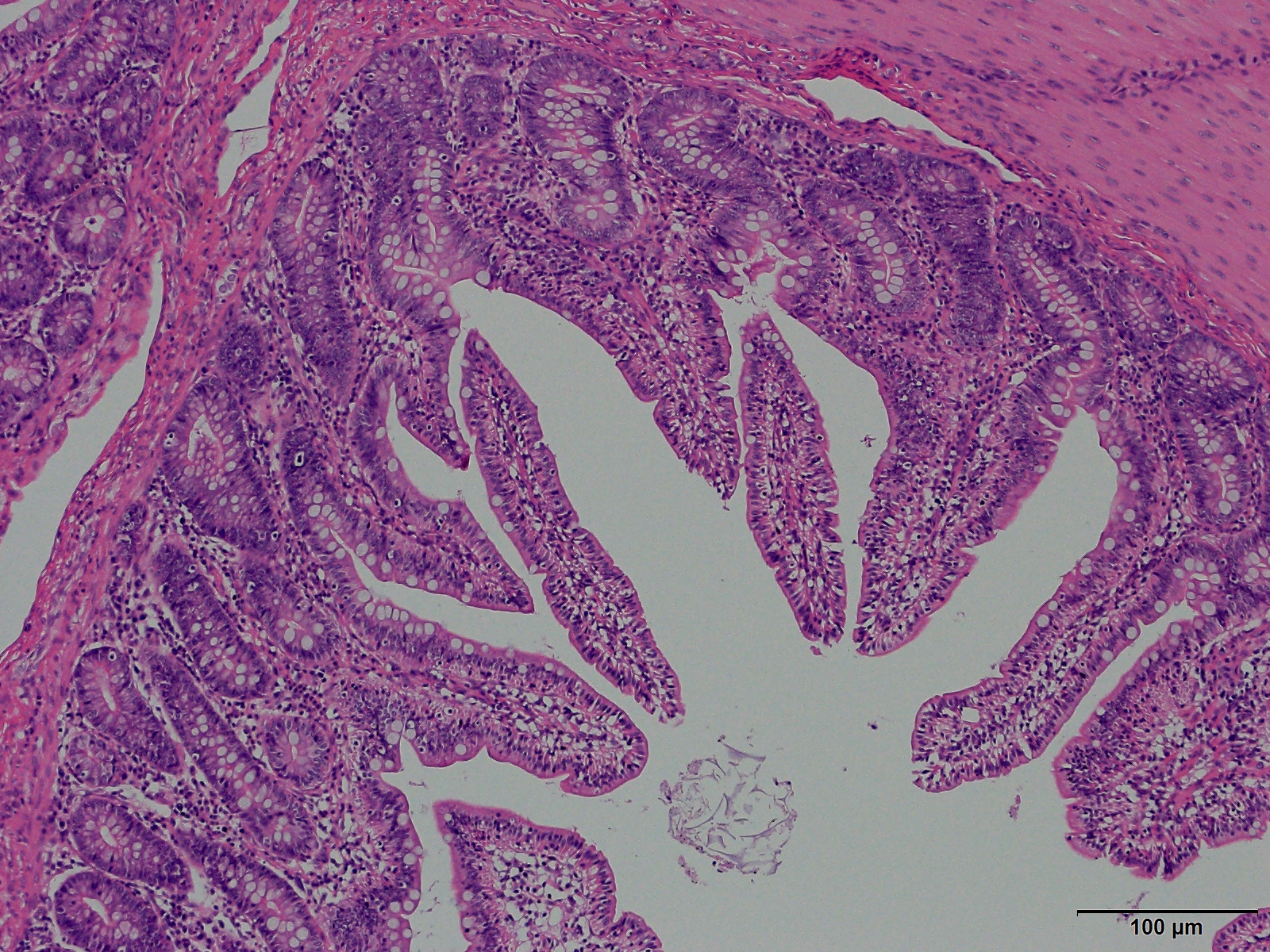

Supplement: Supplementary file 1 [file animals-16-01400-s001.zip › 3. Ileum/0 CEO group/Ileum-1-8.jpg]

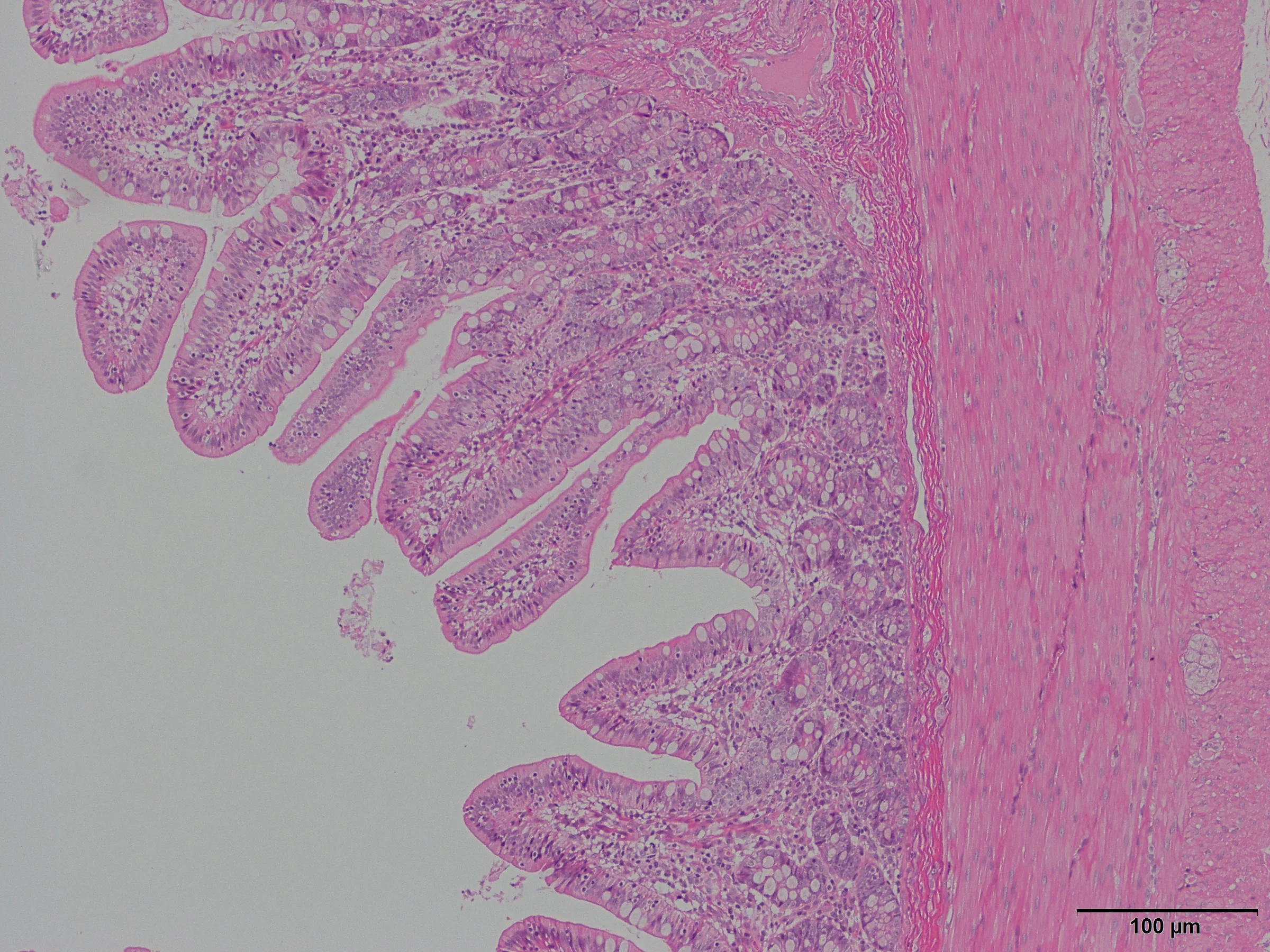

Supplement: Supplementary file 1 [file animals-16-01400-s001.zip › 3. Ileum/120 mg kg CEO group/Ileum-2-1.webp]

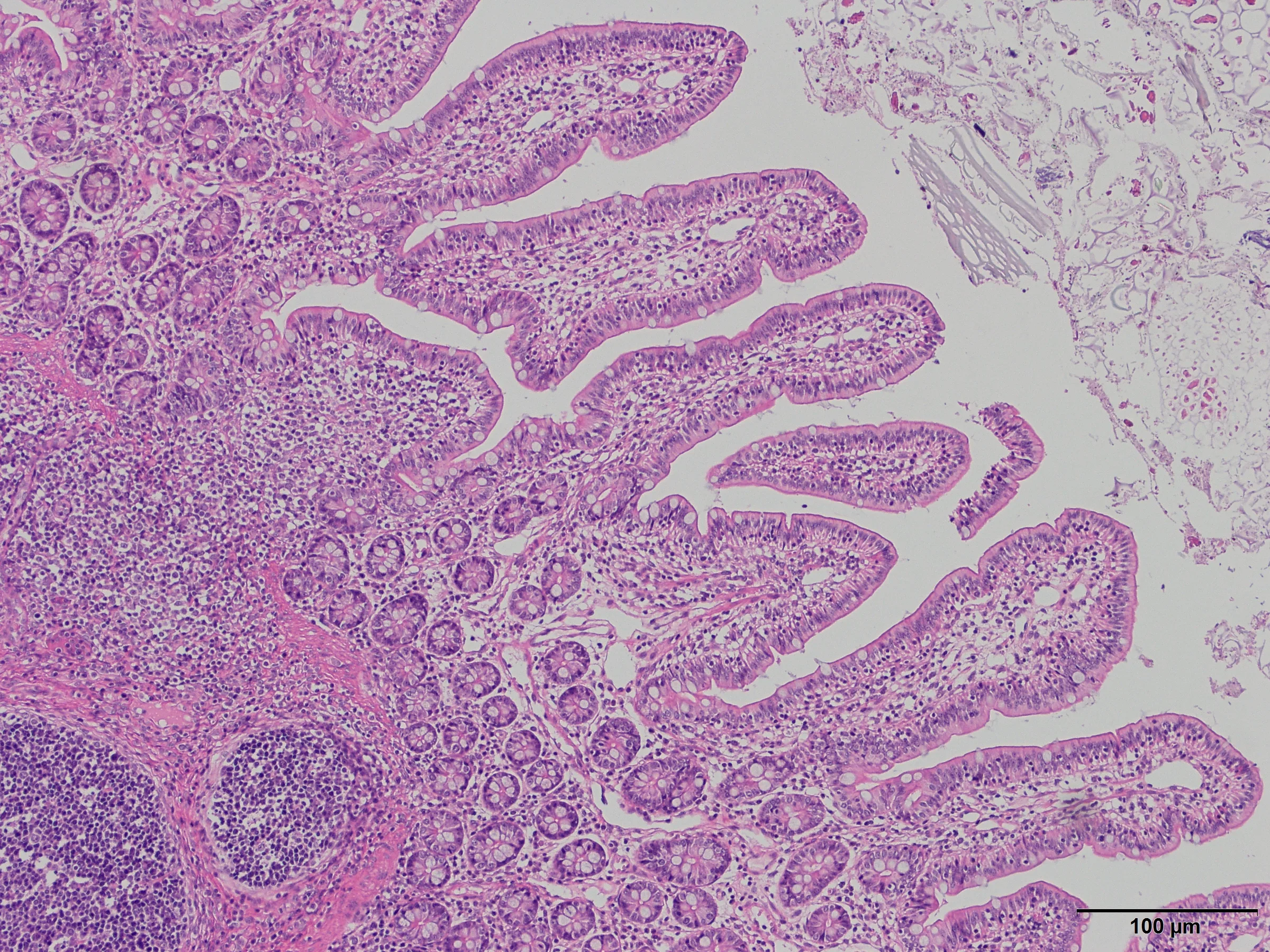

Supplement: Supplementary file 1 [file animals-16-01400-s001.zip › 3. Ileum/120 mg kg CEO group/Ileum-2-2.webp]

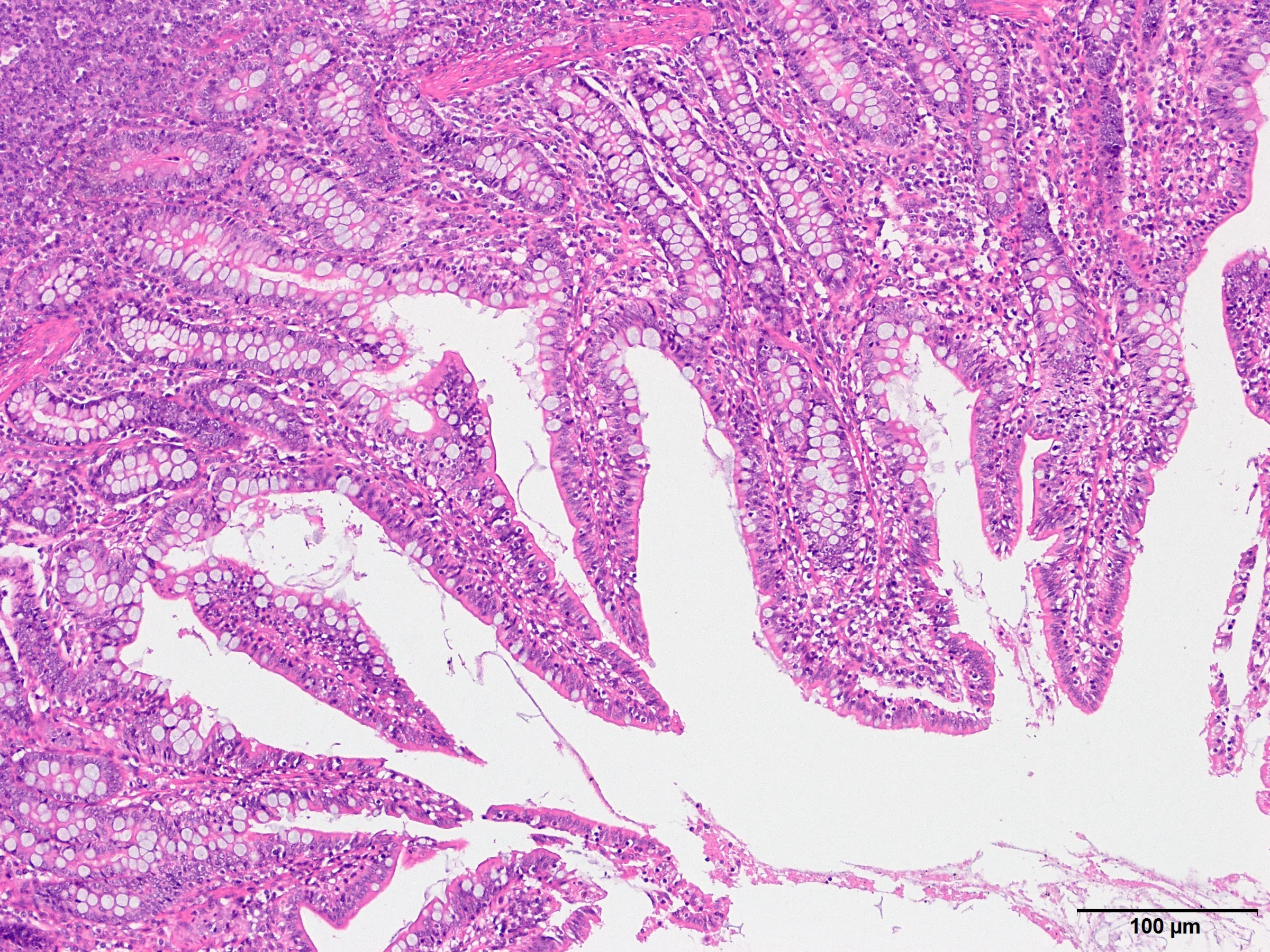

Supplement: Supplementary file 1 [file animals-16-01400-s001.zip › 3. Ileum/120 mg kg CEO group/Ileum-2-3.webp]

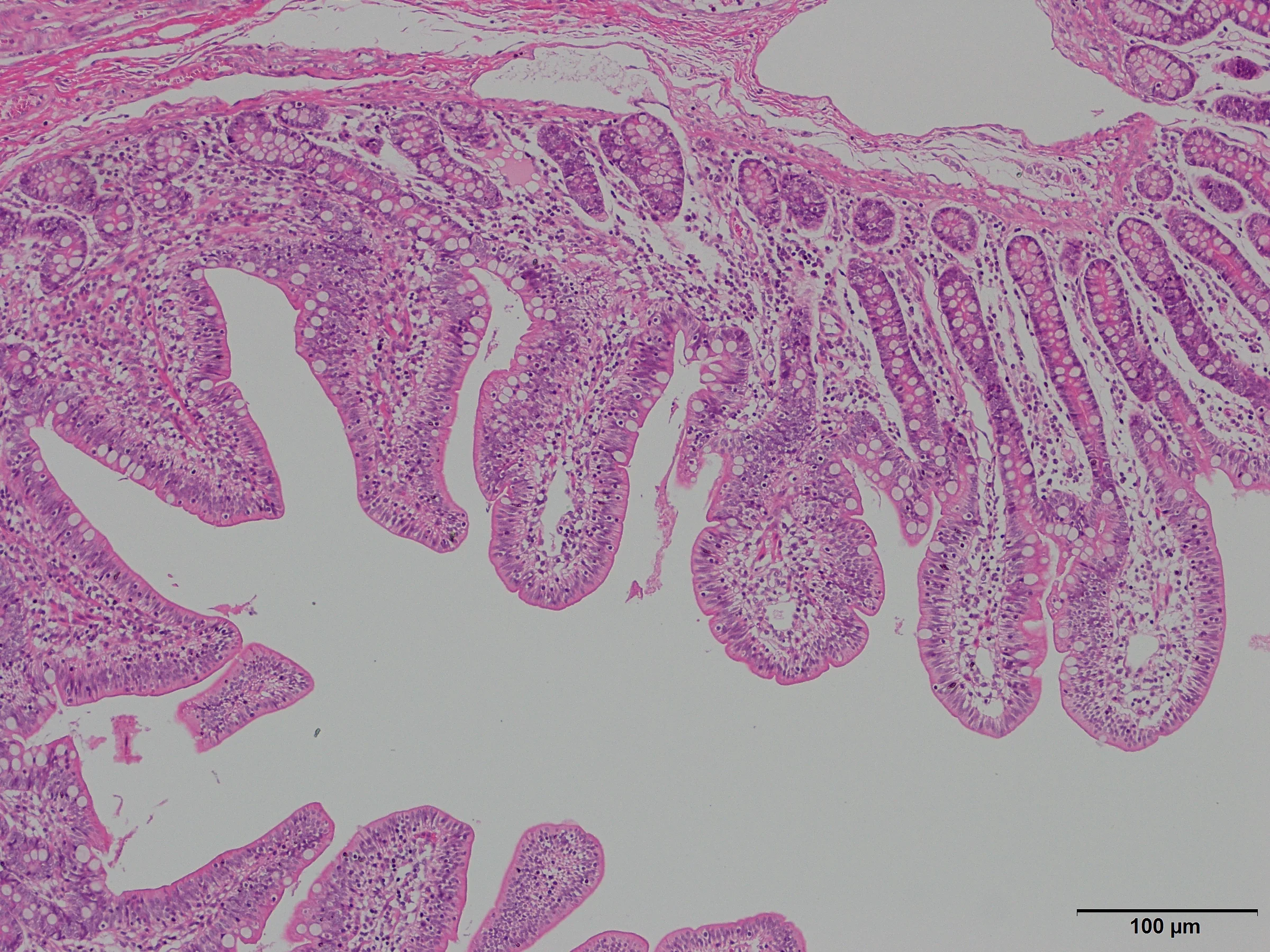

Supplement: Supplementary file 1 [file animals-16-01400-s001.zip › 3. Ileum/120 mg kg CEO group/Ileum-2-4-Figure 3A.webp]

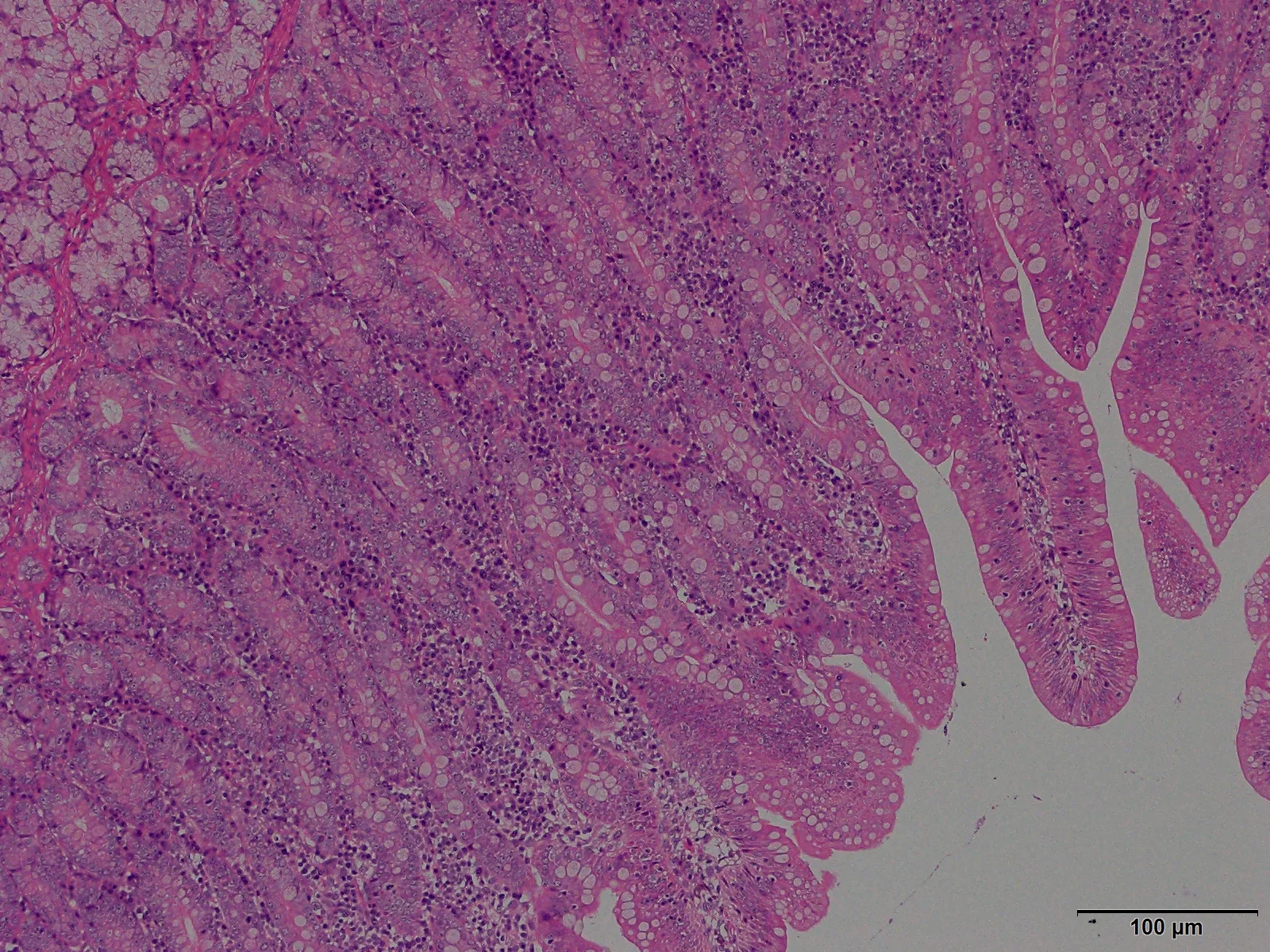

Supplement: Supplementary file 1 [file animals-16-01400-s001.zip › 3. Ileum/120 mg kg CEO group/Ileum-2-5.webp]

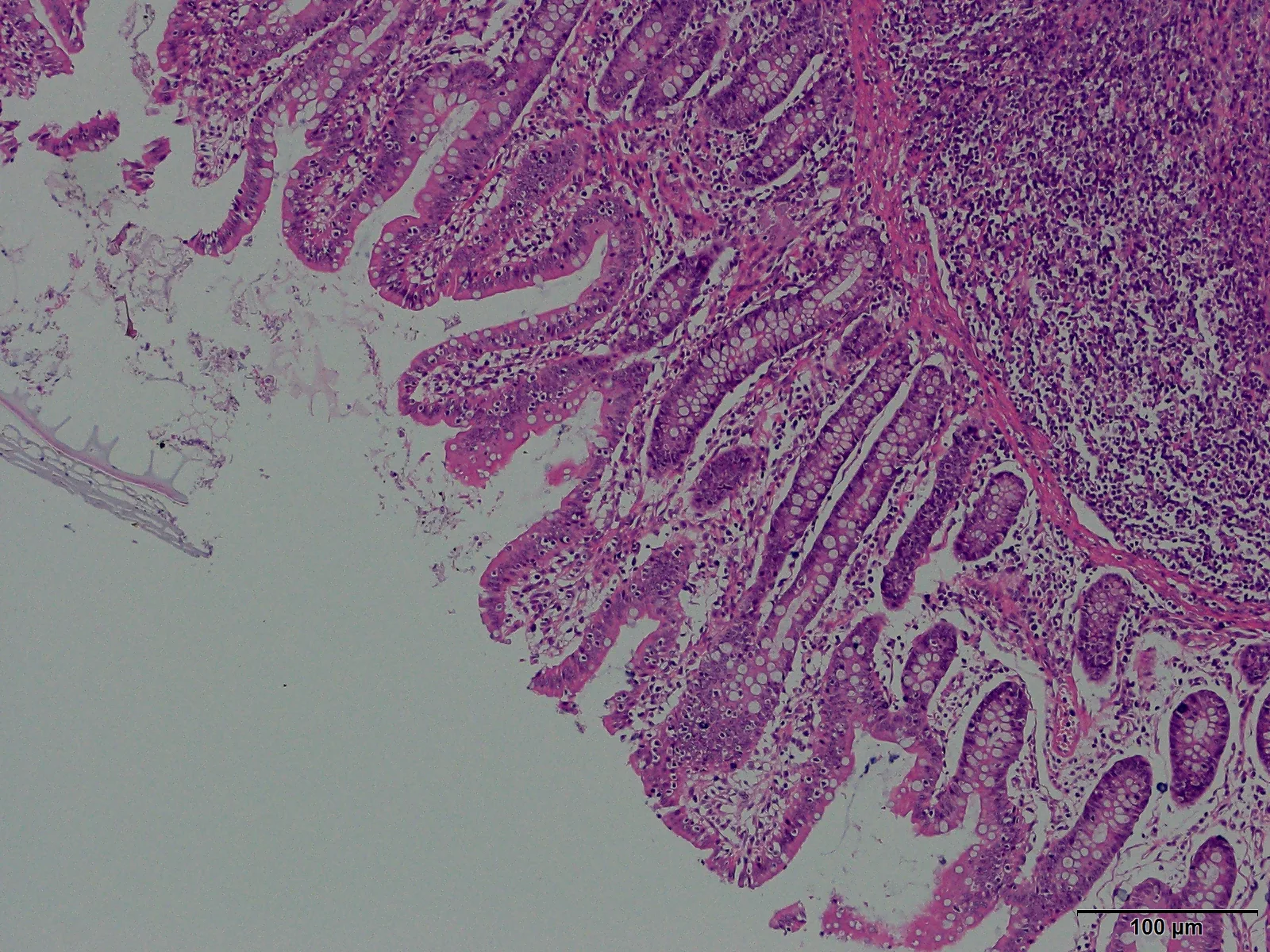

Supplement: Supplementary file 1 [file animals-16-01400-s001.zip › 3. Ileum/120 mg kg CEO group/Ileum-2-6.webp]

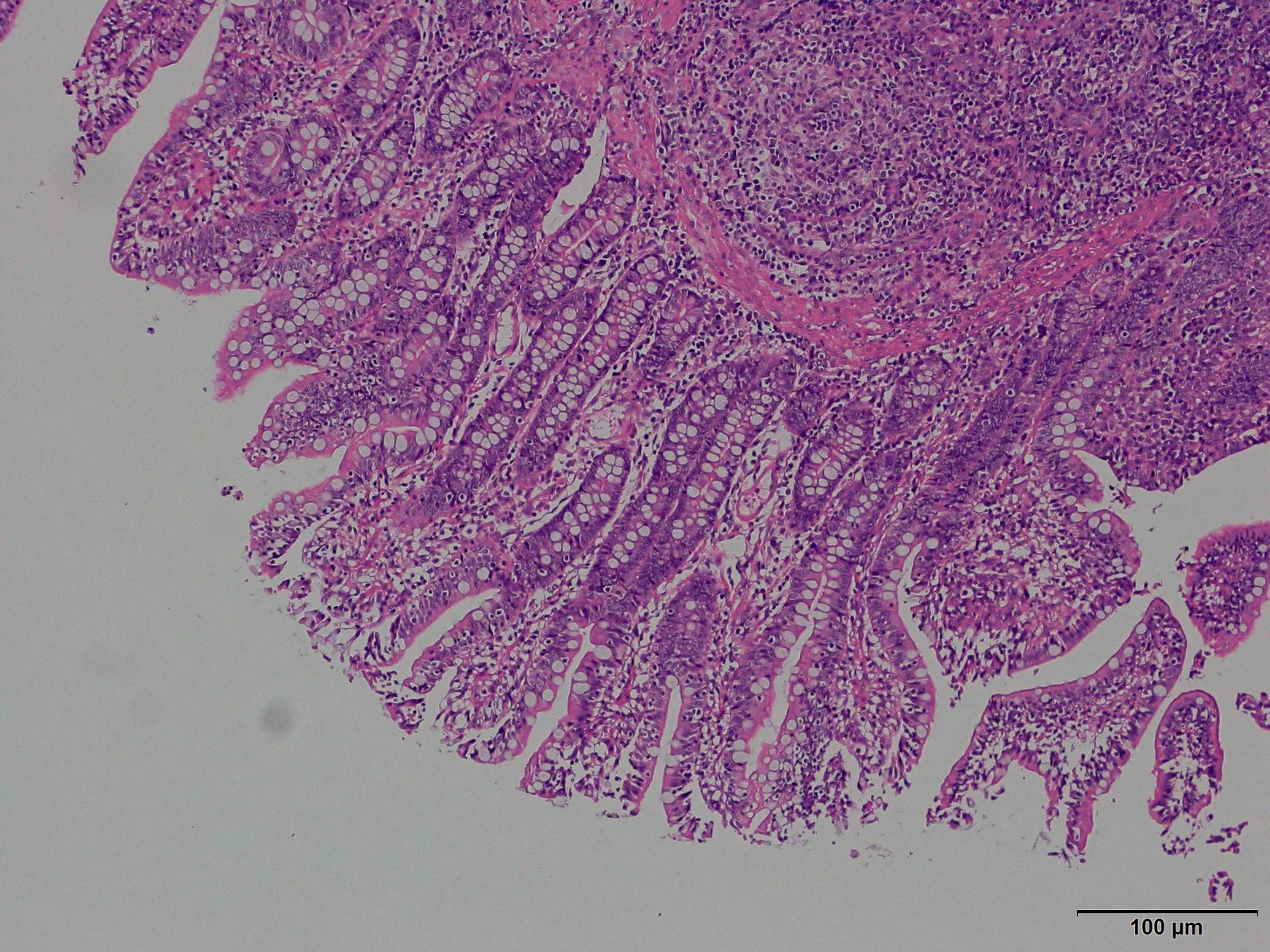

Supplement: Supplementary file 1 [file animals-16-01400-s001.zip › 3. Ileum/120 mg kg CEO group/Ileum-2-7.webp]

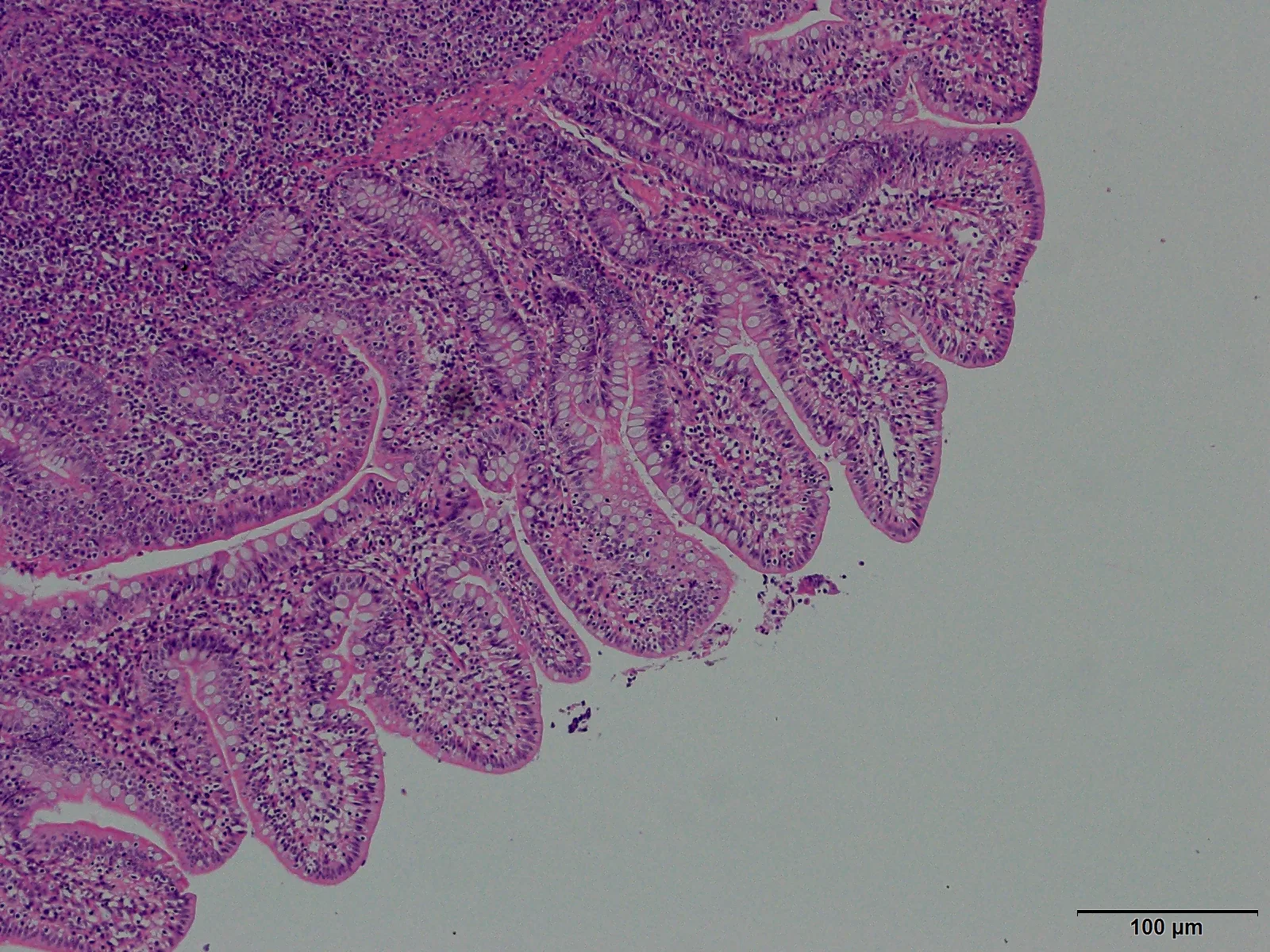

Supplement: Supplementary file 1 [file animals-16-01400-s001.zip › 3. Ileum/120 mg kg CEO group/Ileum-2-8.webp]

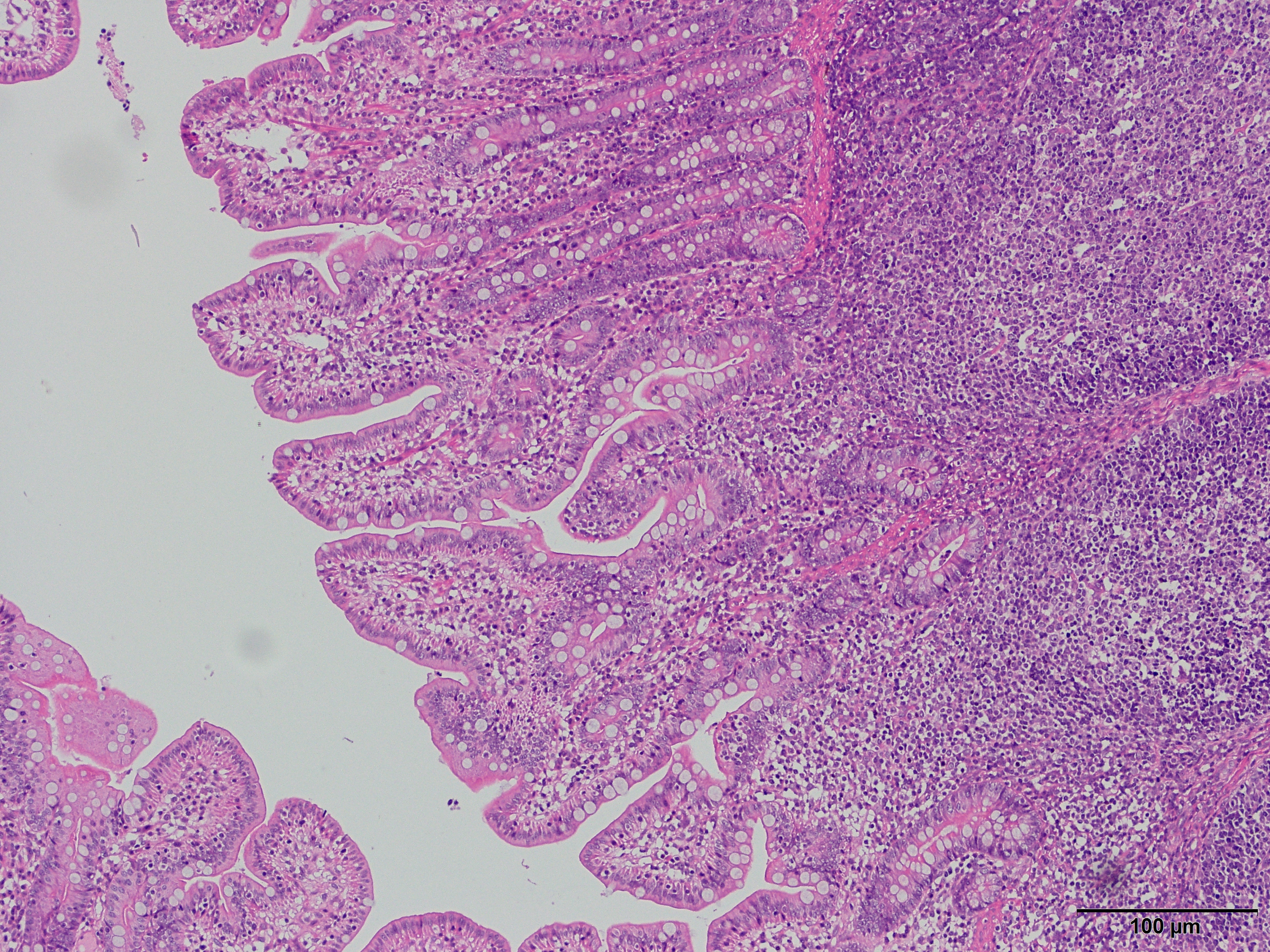

Supplement: Supplementary file 1 [file animals-16-01400-s001.zip › 3. Ileum/180 mg kg CEO group/Ileum-3-1.jpg]

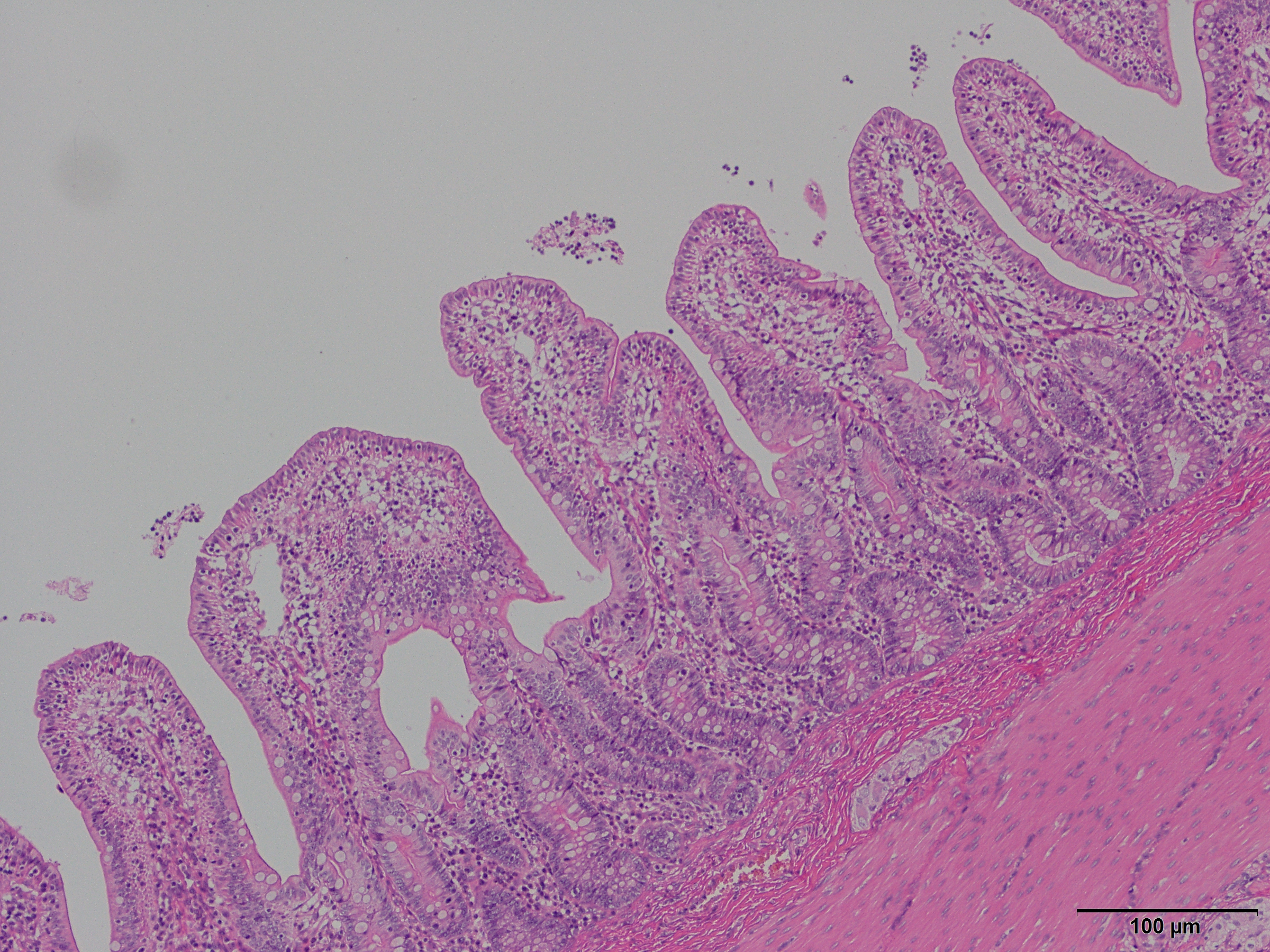

Supplement: Supplementary file 1 [file animals-16-01400-s001.zip › 3. Ileum/180 mg kg CEO group/Ileum-3-2.jpg]

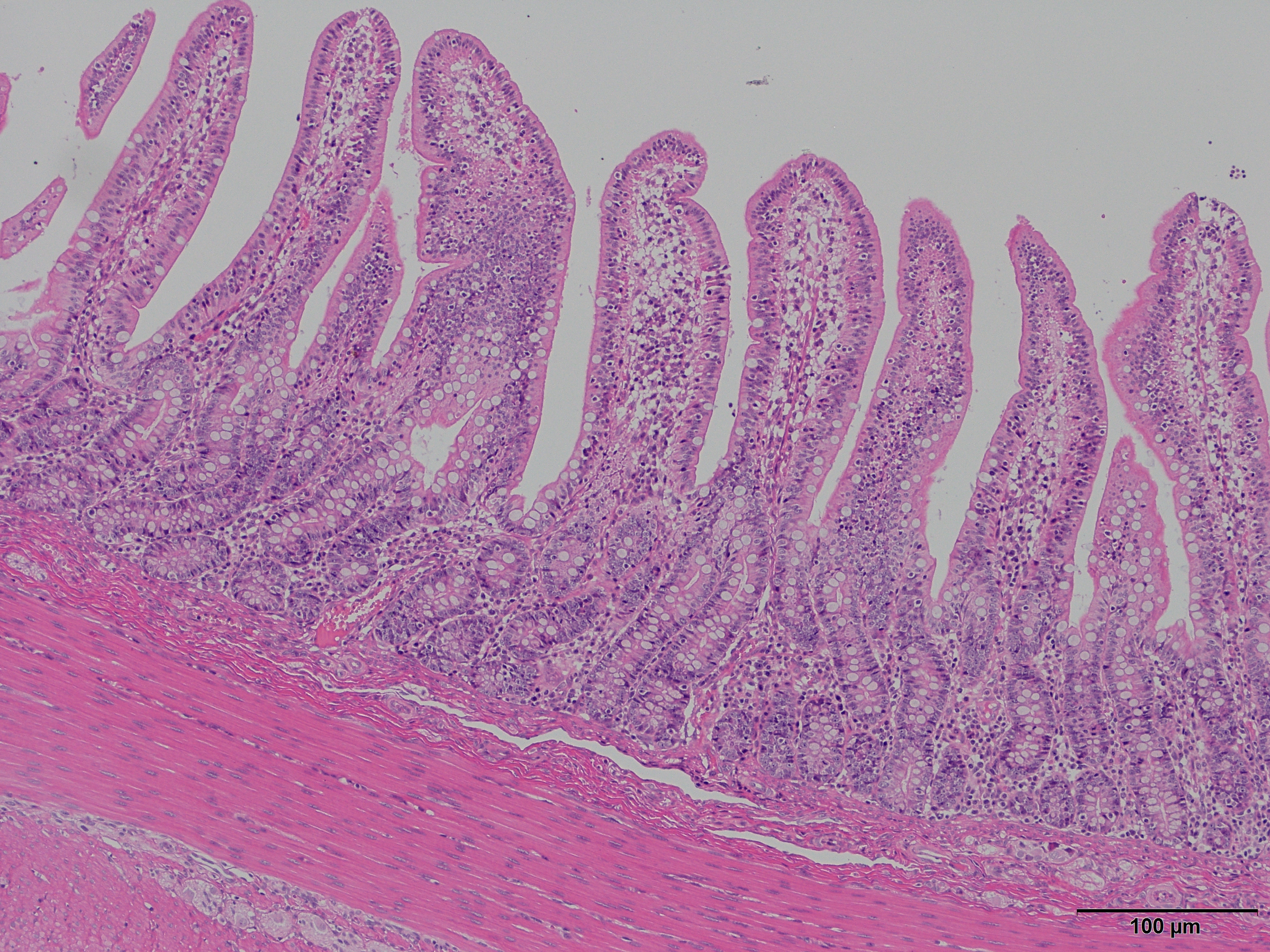

Supplement: Supplementary file 1 [file animals-16-01400-s001.zip › 3. Ileum/180 mg kg CEO group/Ileum-3-3-Figure 3A.jpg]

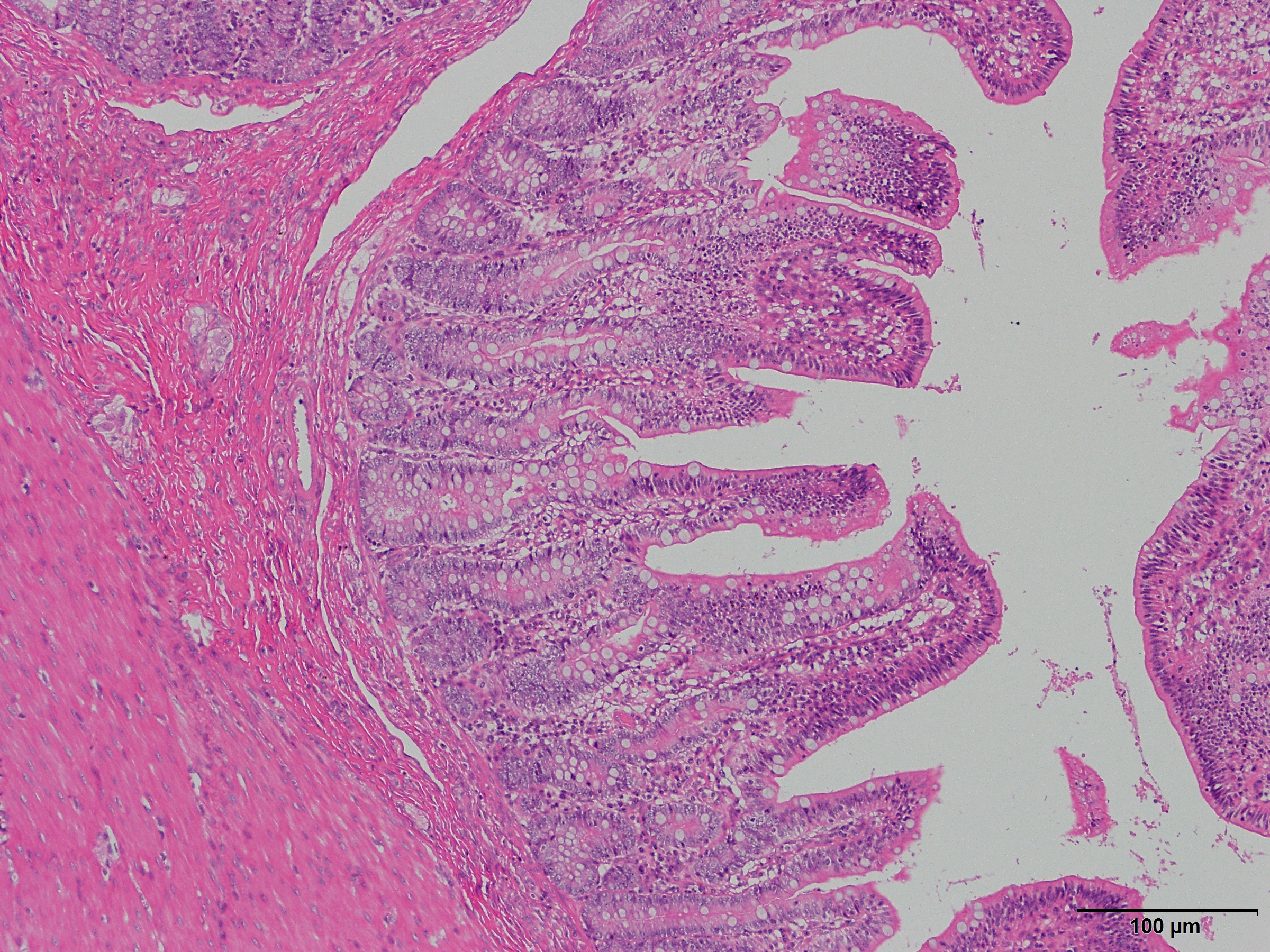

Supplement: Supplementary file 1 [file animals-16-01400-s001.zip › 3. Ileum/180 mg kg CEO group/Ileum-3-4.jpg]

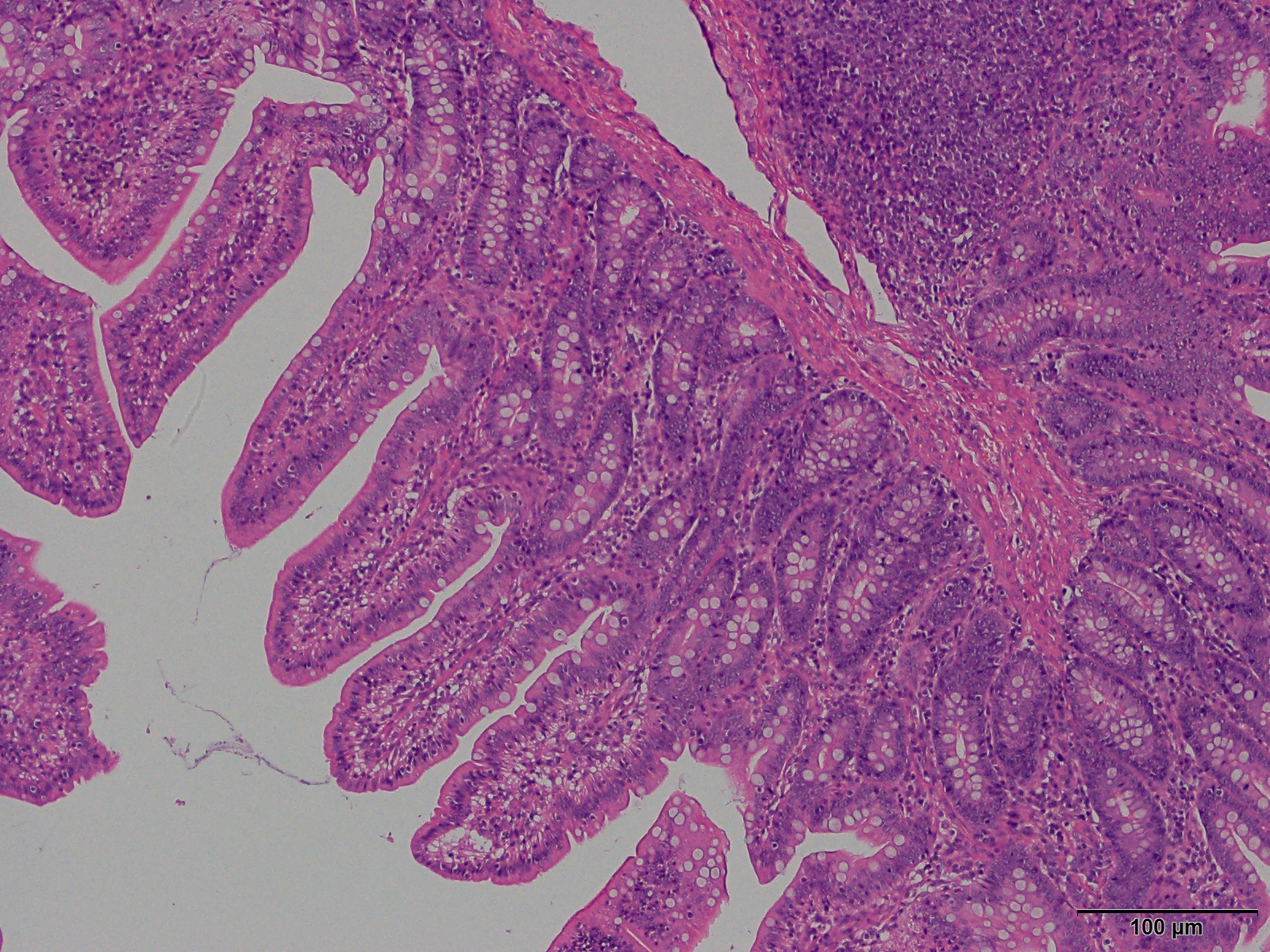

Supplement: Supplementary file 1 [file animals-16-01400-s001.zip › 3. Ileum/180 mg kg CEO group/Ileum-3-5.jpg]

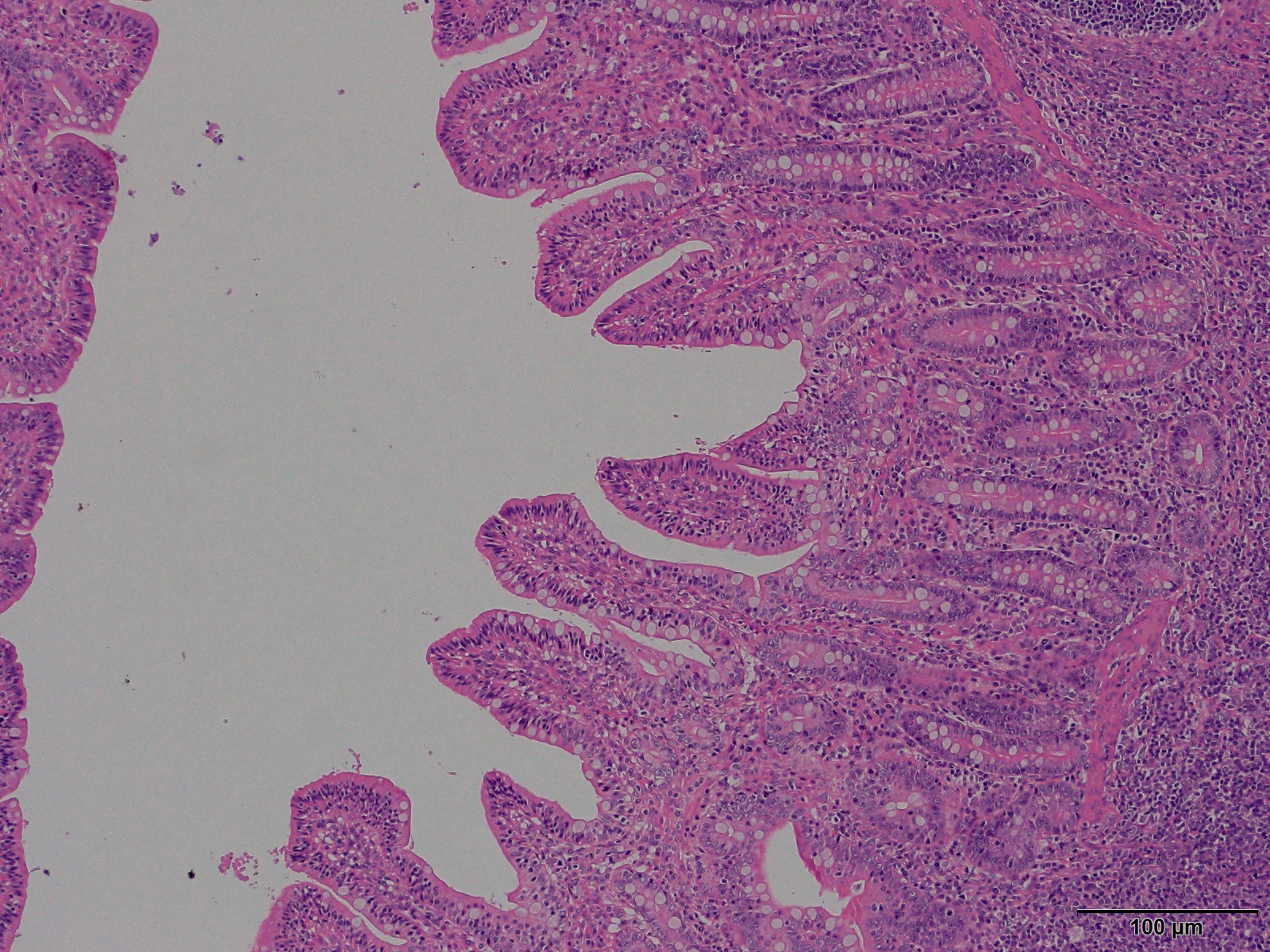

Supplement: Supplementary file 1 [file animals-16-01400-s001.zip › 3. Ileum/180 mg kg CEO group/Ileum-3-6.jpg]

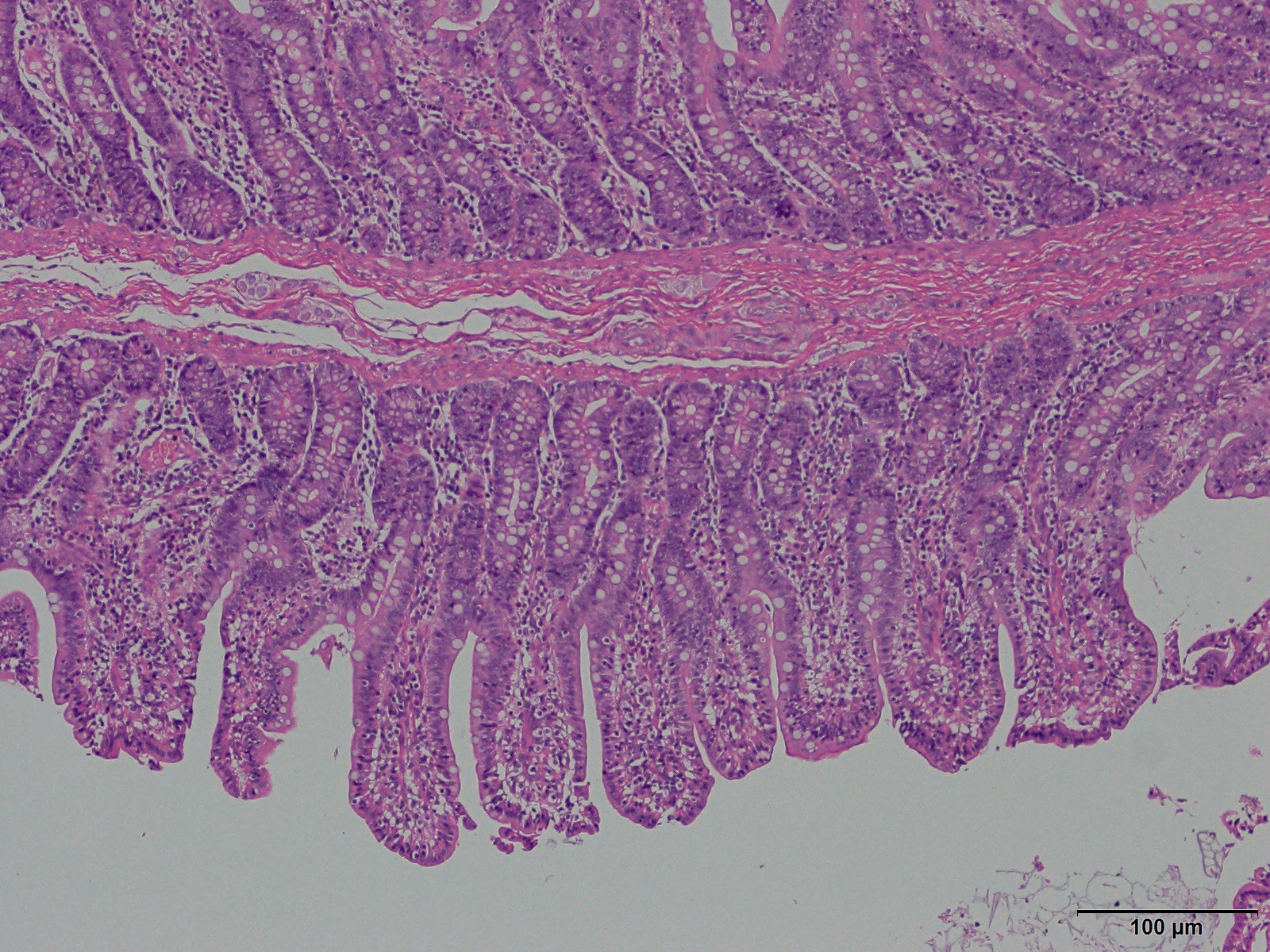

Supplement: Supplementary file 1 [file animals-16-01400-s001.zip › 3. Ileum/180 mg kg CEO group/Ileum-3-7.jpg]

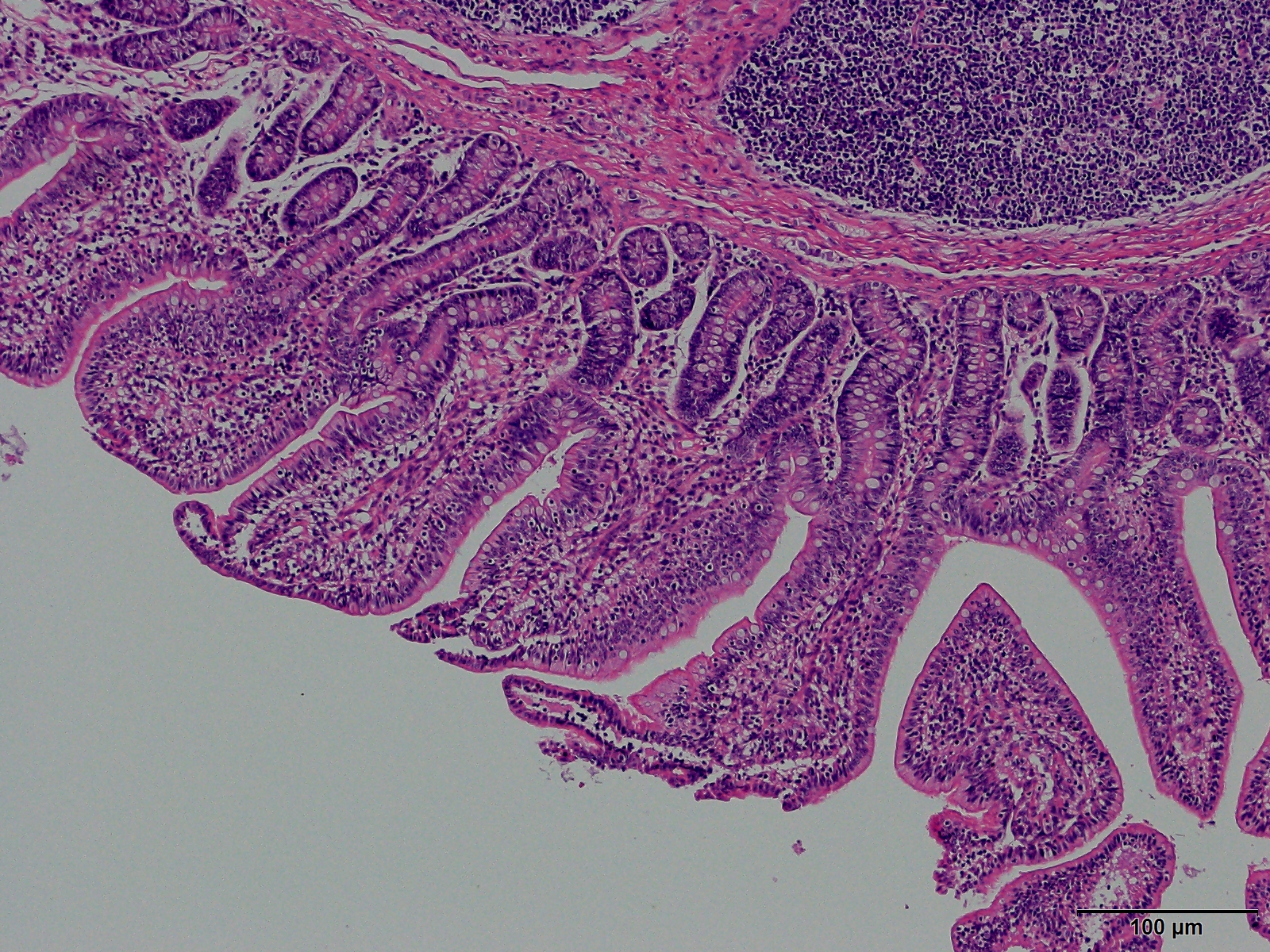

Supplement: Supplementary file 1 [file animals-16-01400-s001.zip › 3. Ileum/180 mg kg CEO group/Ileum-3-8.jpg]

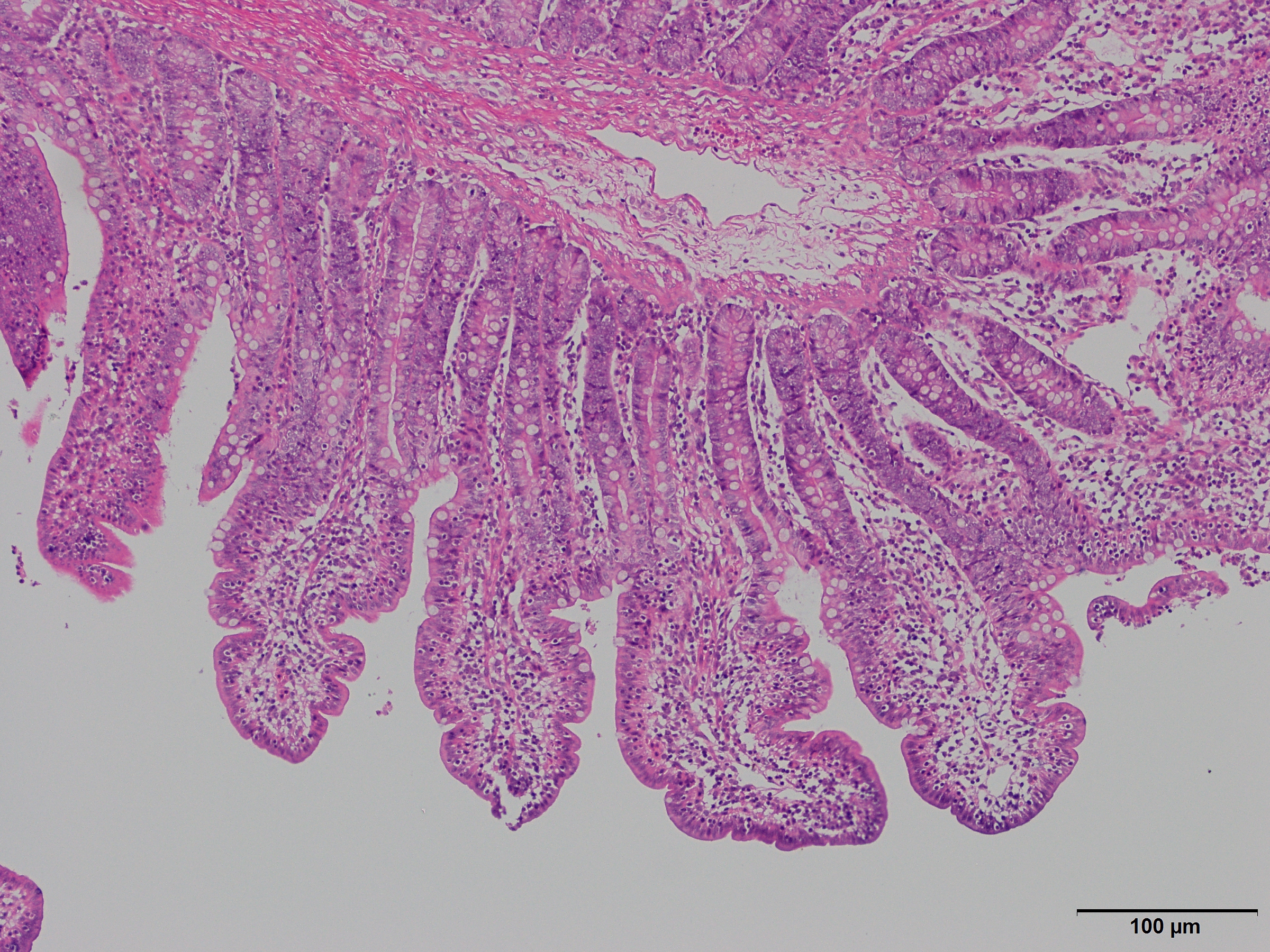

Supplement: Supplementary file 1 [file animals-16-01400-s001.zip › 3. Ileum/240 mg kg CEO group/Ileum-4-1.jpg]

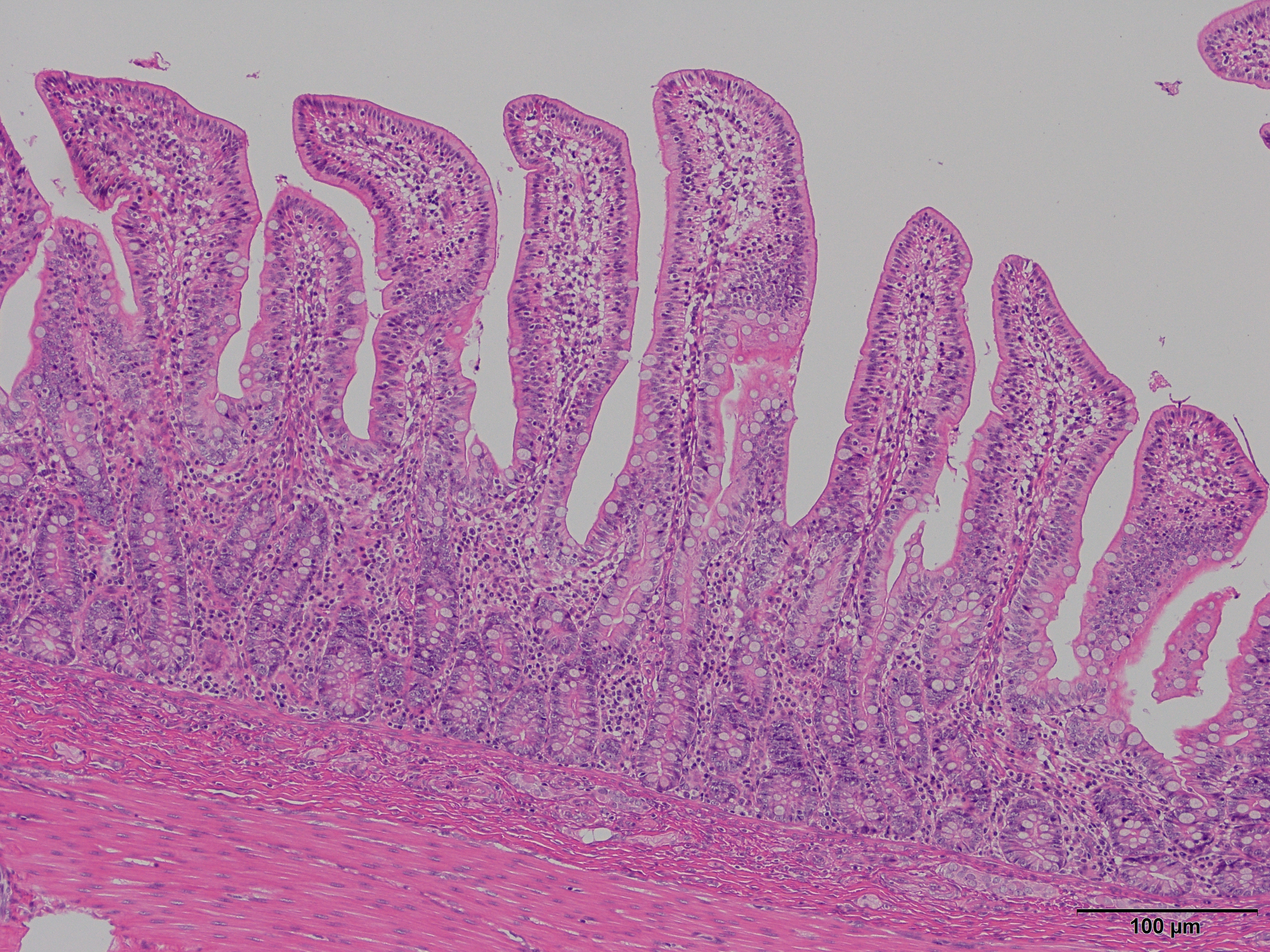

Supplement: Supplementary file 1 [file animals-16-01400-s001.zip › 3. Ileum/240 mg kg CEO group/Ileum-4-2-Figure 3A.jpg]

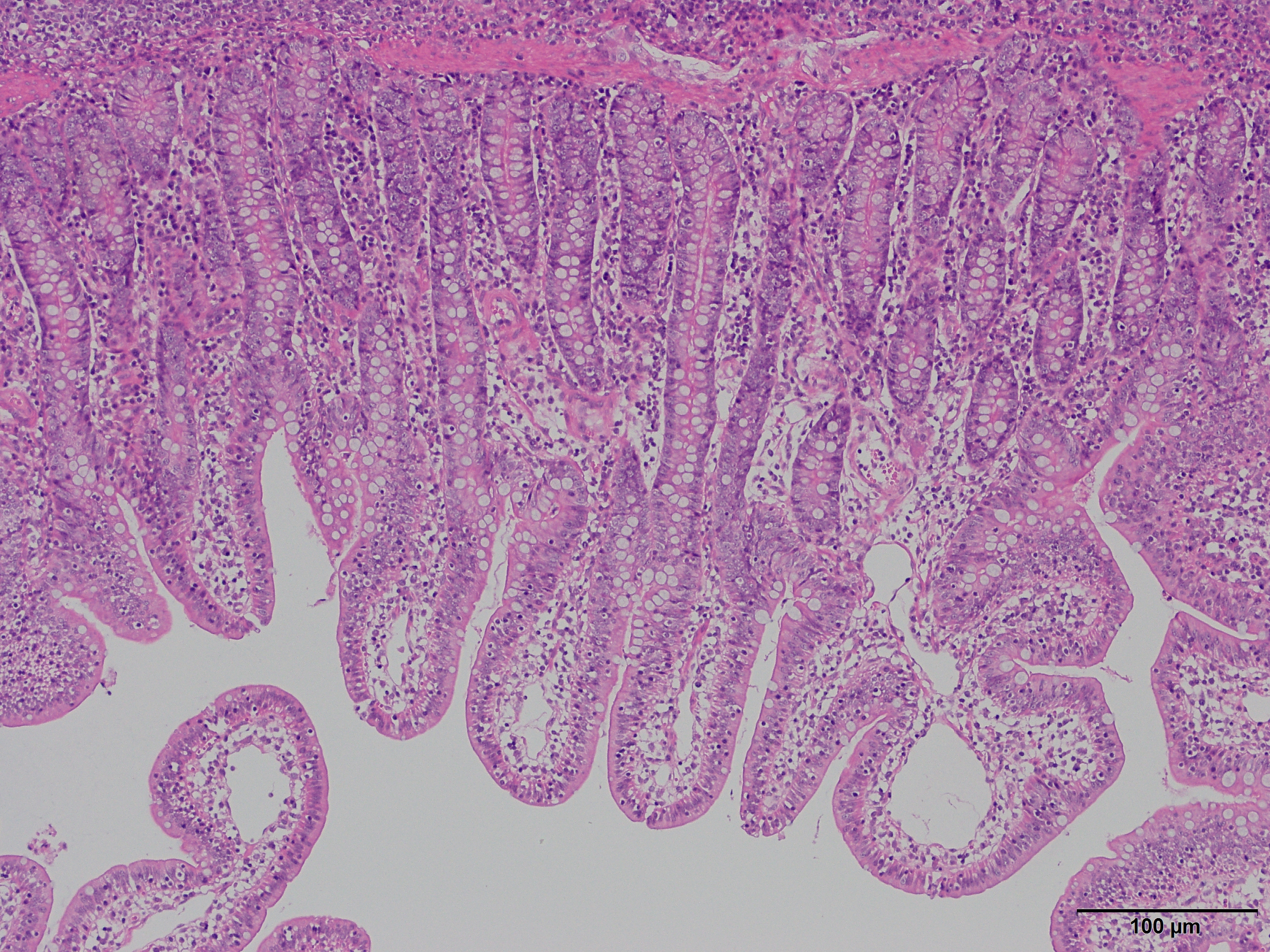

Supplement: Supplementary file 1 [file animals-16-01400-s001.zip › 3. Ileum/240 mg kg CEO group/Ileum-4-3.jpg]

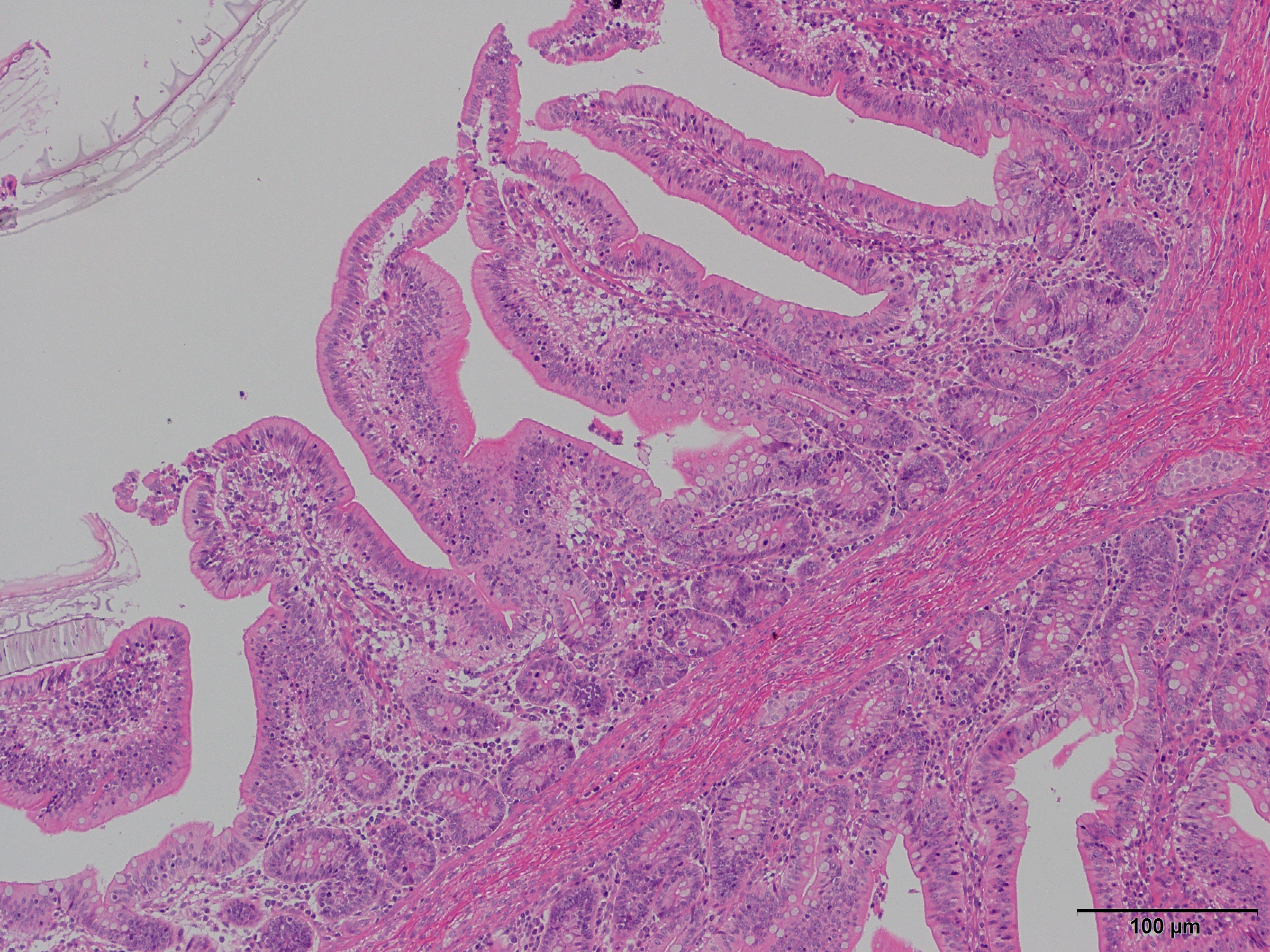

Supplement: Supplementary file 1 [file animals-16-01400-s001.zip › 3. Ileum/240 mg kg CEO group/Ileum-4-4.jpg]

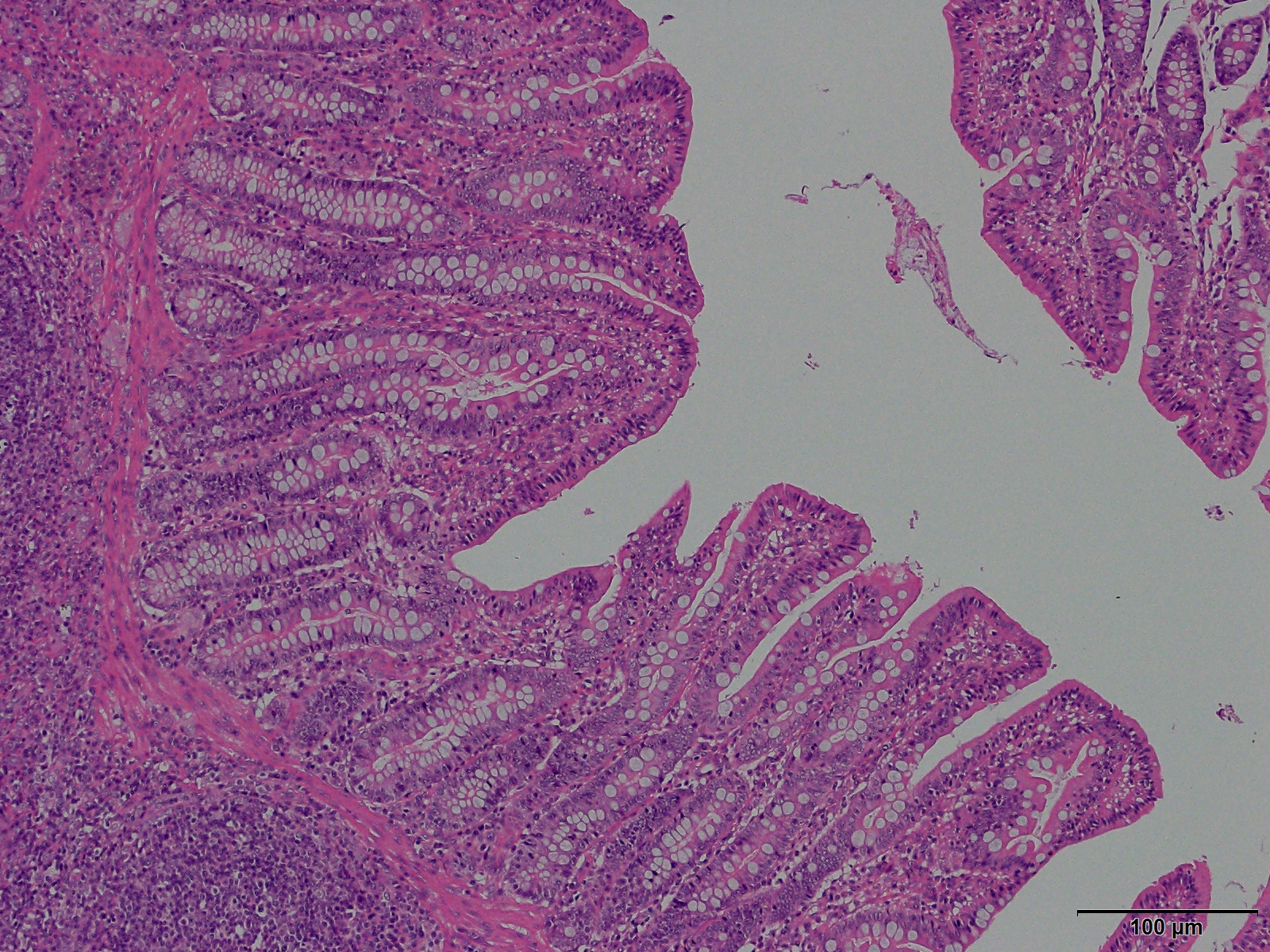

Supplement: Supplementary file 1 [file animals-16-01400-s001.zip › 3. Ileum/240 mg kg CEO group/Ileum-4-5.jpg]

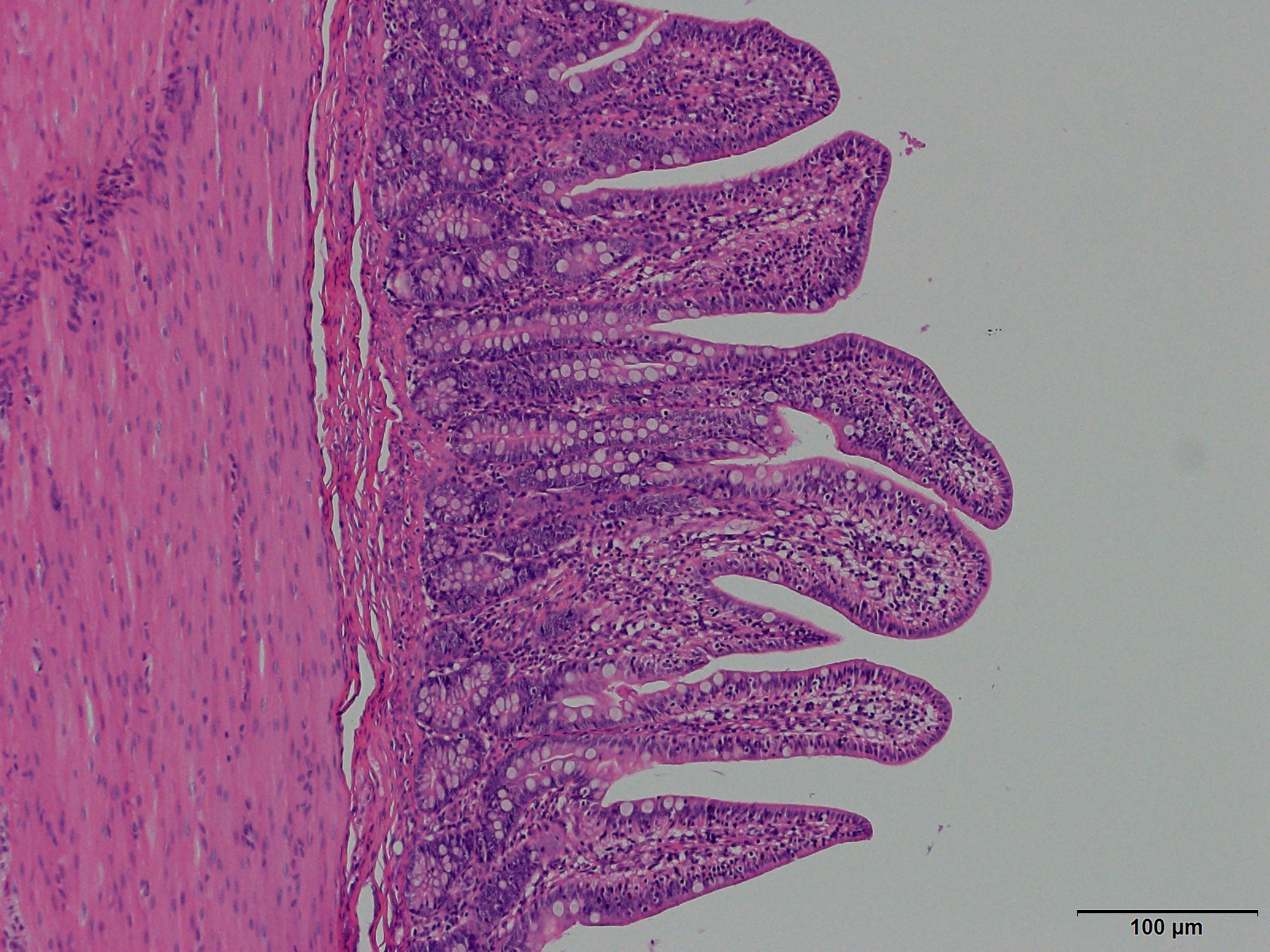

Supplement: Supplementary file 1 [file animals-16-01400-s001.zip › 3. Ileum/240 mg kg CEO group/Ileum-4-6.jpg]

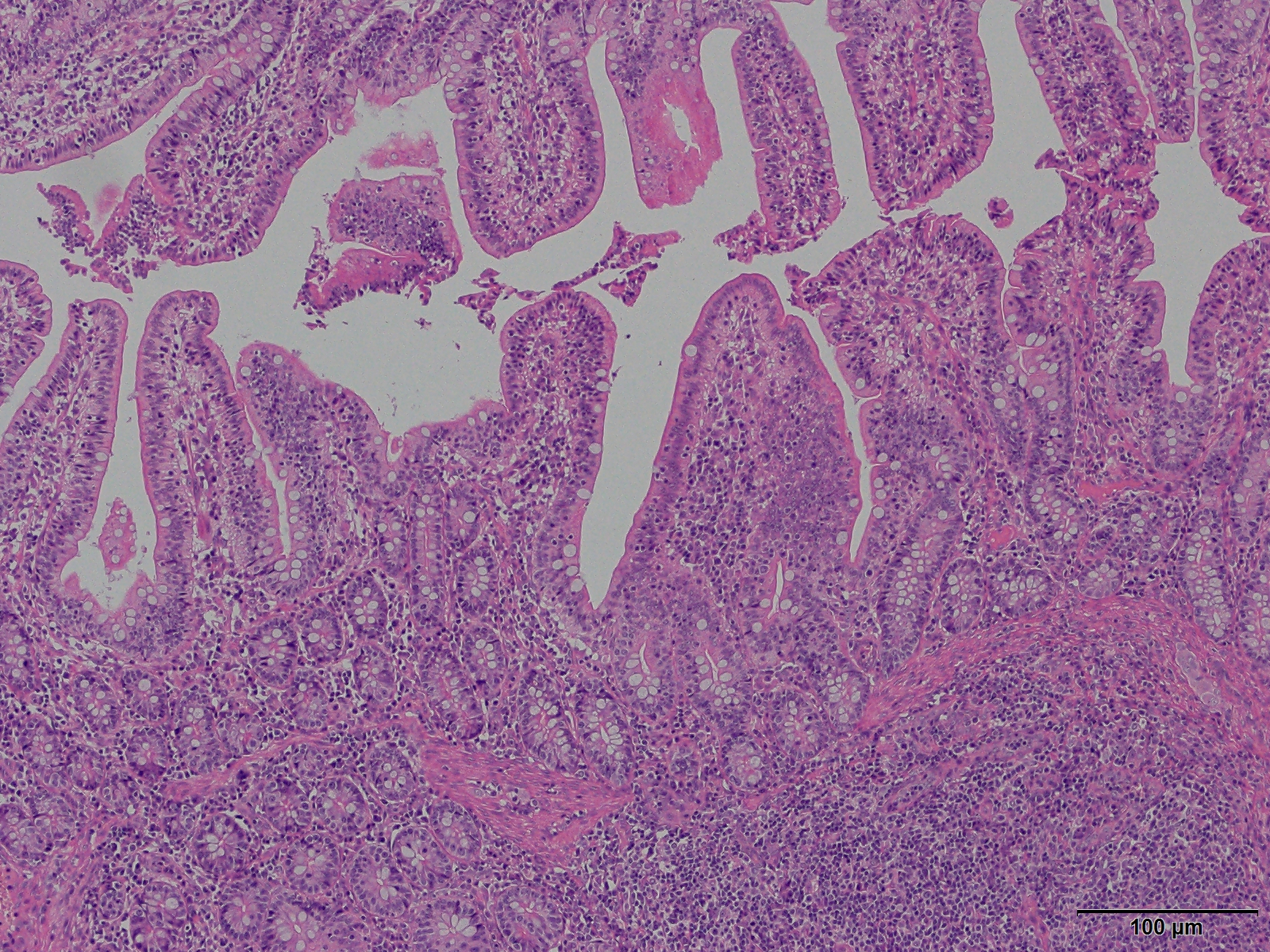

Supplement: Supplementary file 1 [file animals-16-01400-s001.zip › 3. Ileum/240 mg kg CEO group/Ileum-4-7.jpg]

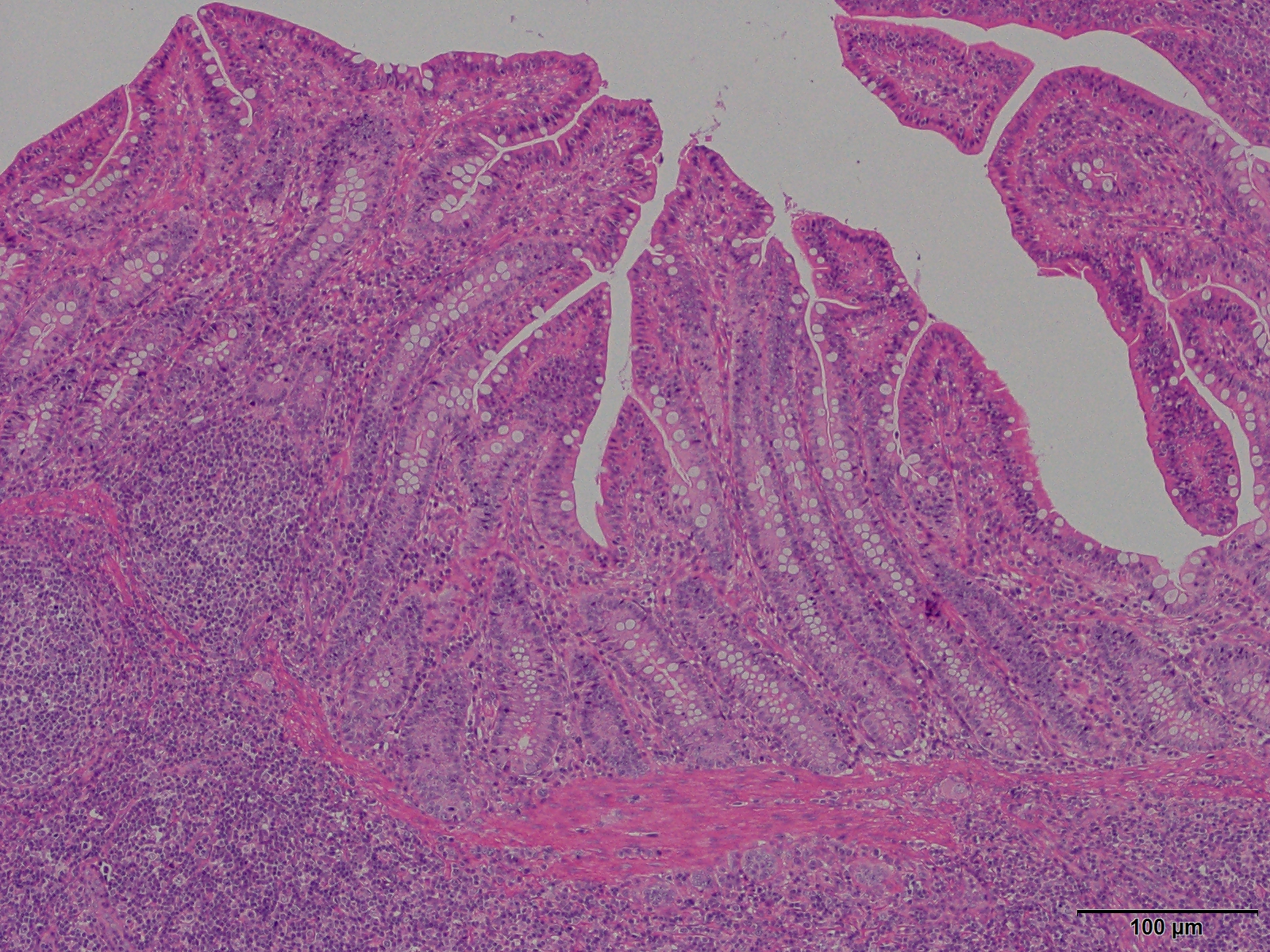

Supplement: Supplementary file 1 [file animals-16-01400-s001.zip › 3. Ileum/240 mg kg CEO group/Ileum-4-8.jpg]
